# Rational Design, Synthesis and Biological Evaluation of Novel Pyrazoline-Based Antiproliferative Agents in MCF-7 Cancer Cells

**DOI:** 10.3390/ph15101245

**Published:** 2022-10-10

**Authors:** Mariam M. Fakhry, Kazem Mahmoud, Mohamed S. Nafie, Ahmad O. Noor, Rawan H. Hareeri, Ismail Salama, Safaa M. Kishk

**Affiliations:** 1 Department of Pharmaceutical Medicinal Chemistry, Faculty of Pharmacy, Suez Canal University, Ismailia 41522, Egypt; 2 Department of Pharmaceutical Chemistry, Faculty of Pharmacy, Egyptian Russian University, Badr 11829, Egypt; 3 Department of Chemistry, Faculty of Science, Suez Canal University, Ismailia 41522, Egypt; 4 Department of Pharmacy Practice, Faculty of Pharmacy, King Abdulaziz University, Jeddah 21589, Saudi Arabia; 5 Department of Pharmacology and Toxicology, Faculty of Pharmacy, King Abdulaziz University, Jeddah 21589, Saudi Arabia

**Keywords:** breast cancer, HER2, EGFR, thiazolyl–pyrazoline, ADMET, docking, drug discovery, industrial development

## Abstract

Breast cancer is a disease in which cells in the breast divide continuously without control. There are great limitations in cancer chemotherapy. Hence, it is essential to search for new cancer therapeutics. Herein, a novel series of EGFR/HER2 dual inhibitors has been designed based on the hybridization of thiazole and pyrazoline fragments. The synthesized compounds were screened for their anti-proliferative activity against MCF-7 breast cancer cell line and MCF-10 normal breast cell line. Interestingly, synthesized compounds **6e** and **6k** showed very potent antiproliferative activity towards MCF-7 with IC_50_ values of 7.21 and 8.02 µM, respectively. Furthermore, enzymatic assay was performed against EGFR and HER2 to prove the dual inhibitory action. Compounds **6e** and **6k** showed potent inhibitory activity for EGFR with IC_50_ of 0.009 and 0.051 µM, respectively, and for HER2 with IC_50_ of 0.013 and 0.027 µM, respectively. Additionally, compounds **6e** and **6k** significantly stimulated apoptotic breast cancer cell death. Compound **6e** was further explored for its anticancer activity in vivo using a Xenograft model. Moreover, computational modeling studies, ADMET studies and toxicity prediction were performed to investigate their potential drug candidates.

## 1. Introduction

Cancer is considered one of the most challenging causes of death in the world, as it is estimated to have caused nearly 10 million deaths in 2020 according to WHO [1,2]. Cancer is not a single disease, but is rather a collective term that describes the distress of the fundamental regulatory genes that are responsible for controlling cell proliferation, differentiation, and survival [3]. The traditional treatment strategies were based on the unspecific death induction targeting the DNA synthesis process without discriminating between tumor cells and rapidly dividing normal cells, which lead to many side effects [4,5,6]. Currently, it is an essential demand to develop more safe and selective molecular targeted chemotherapeutic agents [7]. In this regard, one of the powerful approaches is to target transduction-related macromolecules, especially protein kinases [8,9,10].

**Figure 1:**
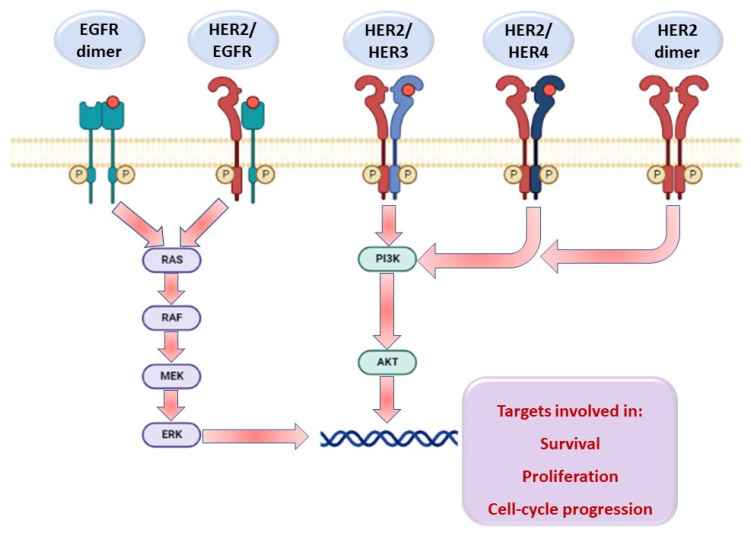
EGF receptors family dimerization mechanism in cell proliferation.

Protein kinases represent the fifth largest human protein family that exert their functions as key regulators of signal transduction mechanisms such as proliferation, metabolism, vascularization, and apoptosis [10,11,12,13]. According to the amino acid that they phosphorylate, protein kinases can be classified into three main classes: tyrosine kinases, Serine–threonine kinases, and Histidine kinases [14]. The epidermal growth factor (EGF) receptor family belongs to tyrosine kinases and is highly expressed or mutated in several cancers such as lung cancer, myeloid leukemia, hormone-dependent and hormone-independent breast cancer. Breast cancer is the leading cause of death among women, with nearly 2.26 million reported new cases worldwide in 2020 as stated in WHO [1]. The EGF receptor family embraces four members: EGFR (HER1/ErbB-1), HER2 (ErbB-2), HER3 (ErbB-3) and HER4 (ErbB-4). These proteins serve as cell surface receptors and consist of an extracellular module and an intracellular kinase domain, connected by a single-helix transmembrane segment and a juxta-membrane segment. EGF receptor family activation is dependent on dimerization, where ligand binding provokes homo- and/or heterodimerization and autophosphorylation on key cytoplasmic residues. The phosphorylated receptor enrolls adapter proteins, which activate complex downstream signaling cascades [15,16] (Figure 1). EGFR and HER2 overexpression in breast cancer is associated with resistance to chemotherapy and poor prognosis [17,18,19,20,21]. Therefore, the usage of dual EGFR/HER2 inhibitors/modulators in breast cancer treatment is an approach worth considering [22,23,24].

Pyrazolines are reported to display various pharmacological activities including anticancer activity [25,26,27,28,29,30,31], in addition to possessing cancer chemo-preventive properties for many pyrazoline-based cytotoxic agents [32,33,34]. For example, compounds I, II and III showed potent inhibitory activity in breast cancer cells MCF-7 with GI_50_ = 0.13 µM, IC_50_ = 0.42 µM and IC_50_ = 34.10 µM, respectively [35,36,37]. Moreover, using methoxy aryl derivatives is reported as increasing anti-proliferative activity, particularly against MCF-7 [38,39].

**Figure 2:**
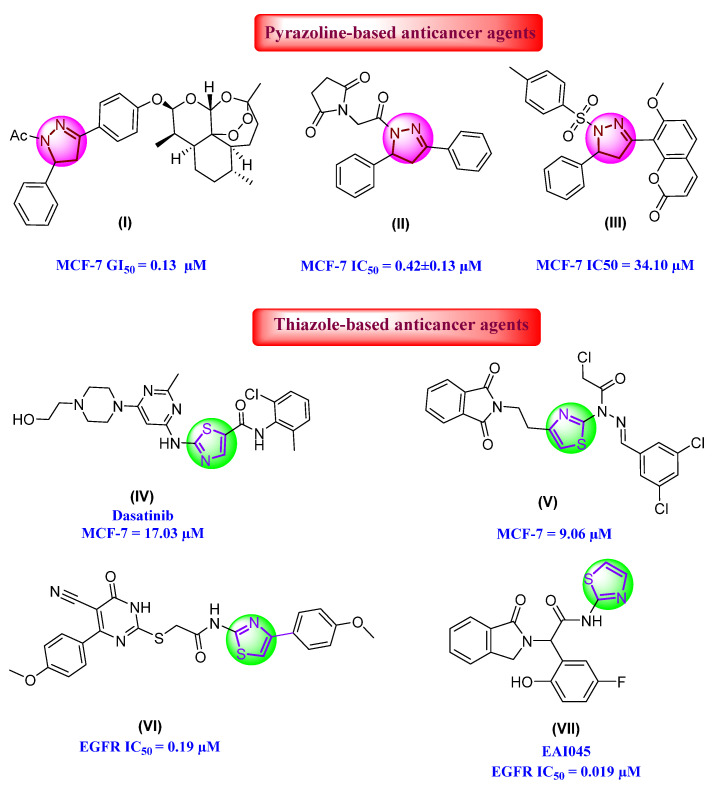
Some reported pyrazoline-based and thiazole-based anticancer agents.

Besides, thiazole-based compounds are intensively studied for their anticancer effects [40,41,42] through the inhibition of different molecular targets such as receptor tyrosine kinases (RTKs), non-receptor tyrosine kinases (nRTKs), phosphatidylinositol-3-kinases (PI3Ks), serine/threonine kinases (STKs), Bcl-2 family, histone deacetylases (HDACs), and tumor necrosis factor-alpha (TNF-α) [43,44,45]. Dasatinib (IV) was confirmed to possess potential tyrosine kinase inhibitory activity [46]. The study of hydrazinyl thiazole derivative (V) revealed high activity against MCF-7 (IC_50_ = 9.06 µM), with selective inhibition to EGFR [47]. In another study, a phenylthiazole derivative (VI) had an IC_50_ = 0.19 µM against EGFR [48]. EAI045 (VII) is a fourth-generation allosteric inhibitor of mutant EGFR (L858R) with high potency (IC_50_ = 0.019 µM) [49] (Figure 2).

### Rationale of the Work

**Figure 3:**
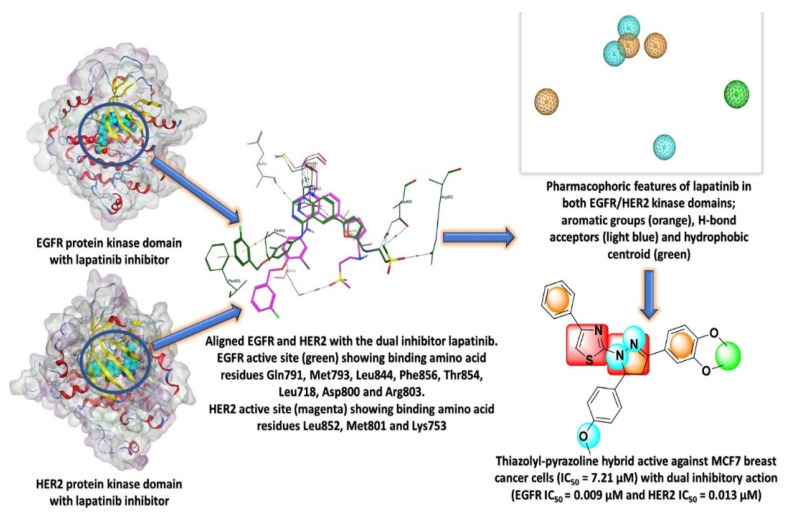
Designed strategy of the thiazolyl–pyrazoline hybrids as dual EGFR/HER2 inhibitors.

Molecular hybridization of both thiazole and pyrazoline scaffolds has been used in multi-targeting kinase inhibitors [50], topoisomerases, apoptosis, with significant antitumor activity against MCF-7 and A595 cell lines [27]. Based on the aforementioned protein kinase inhibitory activity of thiazole and pyrazoline nuclei, especially on EGFR and HER2, a strategy of hybridizing these two luminous moieties was designed to synthesize dual EGFR/HER2 hybrid modulators/inhibitors targeting breast cancer [51]. The new scaffold aimed to find novel lead structures with better activity and lower toxicity [52] (Figure 3).

In this study, a new series of thiazolyl–pyrazoline derivatives was designed, synthesized, and screened for their activity against MCF-7 and MCF-10, and their inhibitory activity was further explored as dual-EGFR/HER2 inhibitors. In addition, the promising compounds were investigated for apoptosis activity and in vivo studies. The molecular modelling studies were performed for the designed compounds to confirm their binding modes to EGFR and HER2 at the ATP binding site.

## 2. Results and Discussion

### 2.1. Chemistry

**Chart 1:**
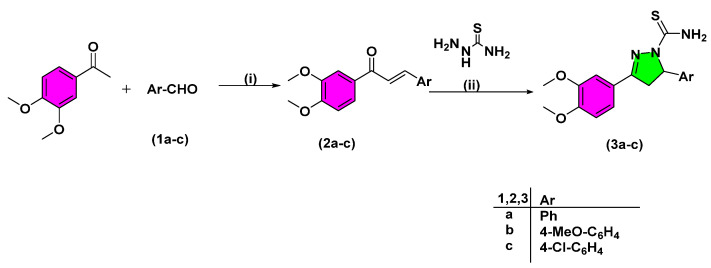
Synthetic pathway for obtaining compounds **3a**–**c**. Reagents and conditions: **i**. Ethanol, 30% NaOH, Stirring, 5 h; **ii**. Abs. EtOH, 1.8 eq NaOH, reflux, 8 h.

**Chart 2:**
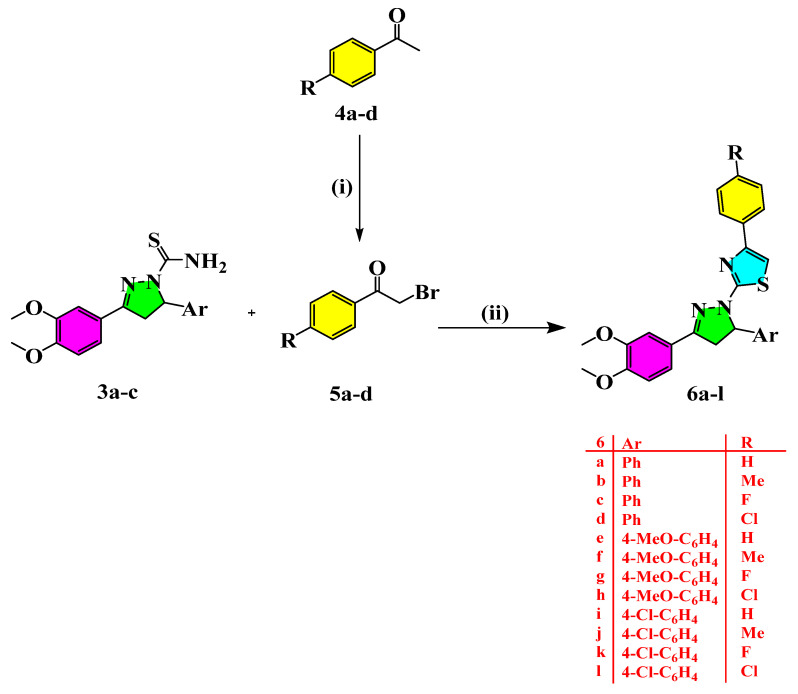
Synthetic pathway for obtaining compounds **6a**–**l**. *Reagents and conditions*: **i**. Br_2_/AcOH/stirring 4–5 h; **ii.** EtOH/DMF, reflux, 5 h.

**Chart 3:**
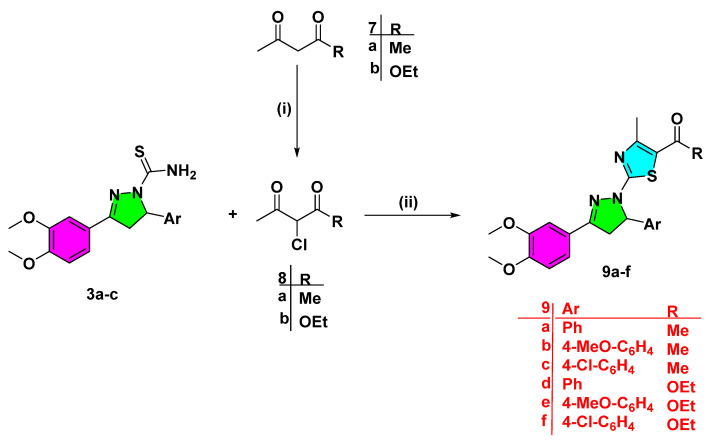
Synthetic pathway for obtaining compounds **9a**–**f**. *Reagents and conditions***: i.** Sulphuryl chloride, stirring, 5 h; **ii.** EtOH, DMF, reflux, 4 h.

The synthetic routes of the thiazolyl–pyrazoline derivatives **3a**–**c**, **6a**–**l** and **9a**–**f** are considered in Chart 1, Chart 2 and Chart 3. First, the 3-(4-aryl)-1-(3,4-dimethoxyphenyl)prop-2-en-1-ones **2a**–**c** were obtained through the Claisen–Schmidt reaction [53] by direct condensation of aromatic aldehydes **1a–c** with dimethoxy acetophenone in the presence of sodium hydroxide [54,55,56]. This step was followed by the reaction of compounds **2a**–**c** with thiosemicarbazide under a basic condition to give 3-(3,4-dimethoxyphenyl)-5-(4-aryl)-4,5-dihydro-1*H*-pyrazole-1-carbothioamides **3a**–**c**, respectively (Chart 1) [57,58]. This reaction proceeded through a condensation reaction followed by subsequent cyclization via Michael addition reaction. The IR spectra for compounds **3a**–**c** showed the characteristic NH_2_ band in the range 3422–3260 cm^−1^, in addition to a band at 1265–1248 cm^−1^ referring to C=S group. Additionally, ^1^H NMR of compounds **3a**–**c** displayed three doublets of doublet (dd) of pyrazoline ring due to ABX pattern. These three (dd) signals of H_A_, H_B_, and H_X_ appeared at *δ* 3.12–3.14, 3.8 and 5.8–5.9 ppm, respectively. Moreover, NH_2_ exchangeable proton signal of compounds **3a**–**c** appeared around *δ* 8.0 ppm. ^13^C NMR of compounds **3a**–**c** revealed the signals for CH_A_H_B_ and CH_X_ carbons of pyrazoline ring around *δ* 42.9 and 62.8 ppm, respectively, as well as C=S signals at *δ* 176.2 ppm.

Next, the reaction of carbothioamide compounds **3a**–**c** with an equimolar amount of phenacyl bromide derivatives **5a–d** [59] in absolute ethanol yielded the corresponding thiazolyl–pyrazoline derivatives **6a**–**l** in good yields (Chart 2) [60]. Compounds **5a–d** were prepared by bromination of corresponding acetophenone using bromine and acetic acid in diethyl ether as solvent. The IR spectra of compounds **6a**–**l** showed the disappearance of the NH_2_ characteristic band in the region 3422–3260 cm^−1^. ^1^H NMR spectra also revealed the absence of NH_2_ proton around *δ* 8.0 ppm. ^1^H NMR spectra of compounds **6a**–**l** showed the signals of aromatic protons at *δ* 7.8–6.9 ppm to be assignable to thiazolyl and phenyl protons. In addition, an extra signal at *δ* 2.3 ppm appeared due to the presence of CH_3_ in compounds **6b**, **6f**, and **6j**. The ABX pattern was confirmed by COSY spectra for compound **6k** to prove the formation of the pyrazoline ring of two non-equivalent protons at C4 (H_A_ and H_B_) and one proton at C5 (H_X_). ^13^C NMR spectra of compounds **6a**–**l** showed the signals of the aromatic carbons at 103.5–165 ppm. ^13^C NMR spectra of compounds **6g**, **6c**, and **6k** (4-FC_6_H_4_) showed the splitting of the three carbon atoms at *δ* 115.9–115.7, 128–127.8, 131.7–131.54 ppm due to their coupling with the fluorine atom. Also, ^13^C NMR spectra revealed the appearance of singlet signal at *δ* 21.2–21.3 ppm which belongs to CH_3_ (tolyl carbon) of compounds **6b**, **6f**, and **6j**. Mass spectra of compounds **6a–l** showed the molecular ions peak in accord with their molecular weight.

Furthermore, compounds **8a**–**b** were synthesized by treating pentane-2,4-dione or methyl 3-oxobutanoate with sulfuryl chloride (SO_2_Cl_2_) [61]. Treatment of 3-(3,4-dimethoxyphenyl)-5-aryl-4,5-dihydro-1*H*-pyrazole-1-carbothioamide derivatives **3a**–**c** with 3-chloropentane-2,4-dione **8a** or ethyl 2-chloro-3-oxobutanoate **8b** in refluxing ethanol produced the intended thiazolyl–pyrazoline derivatives **9a**–**f** (Chart 3) [62]. IR spectra of compounds **9a–c** showed a band at 1617–1613 cm^−1^ representing the C=O group. ^1^H NMR spectra of compounds **9a–c** elicited two singlet signals at *δ* 2.39 and 2.37 ppm belonging to two CH_3_ of methyl and acetyl group, respectively, whereas their ^13^C NMR displayed signals at *δ* 19.1, 30.1, and 189 were attributed to *SP^3^* carbons of two CH_3_ and C=O carbons, respectively.

IR spectra of compounds **9d**–**f** displayed a band at 1708–1695 cm^−1^ representing C=O. ^1^H NMR spectrum also showed a typical quartet–triplet pattern of the ethyl protons at *δ* 4.2 and 1.25 ppm, respectively. However, signals of thiazole CH_3_ appeared at *δ* 2.36–2.35 ppm. Furthermore, ^13^C NMR spectrum revealed the signals of ethyl carbons at *δ* 14.8 and 60.6 ppm, and signal at 165.1–165.2 ppm attributed C=O of ester.

### 2.2. Biological Evaluation

#### 2.2.1. Cytotoxicity

**Figure 4:**
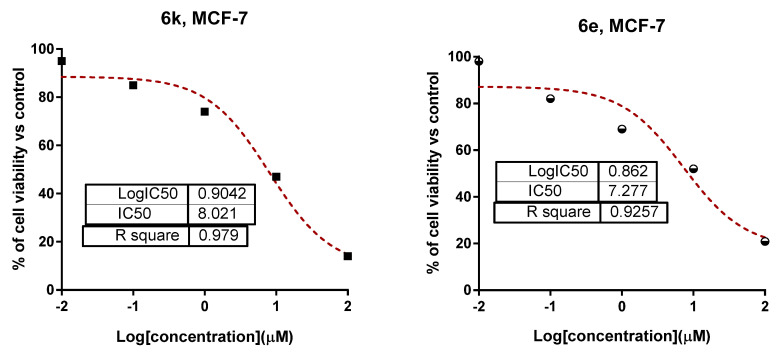
Dose–response nonlinear regression curve fitting the percentage of cell viability vs. log [conc. µM], R square = 1 using the GraphPad prism.

The MTT assay was used to test the activity and selectivity of all synthesized compounds for cytotoxic activity against MCF-7 cancer cells and MCF-10A normal breast cells. As seen in Table 1, compounds **3c**, **6b** and **6f** were found to be more cytotoxic against MCF-7, with IC_50_ ranges of 11.09, 13.36 and 11.05 µM, respectively. Interestingly, compounds **6e**, **9f** and **6k** exhibited remarkable cytotoxic activities, with IC_50_ values of 7.21, 8.35 and 8.02 µM, respectively, compared to Lapatinib (IC_50_ = 7.45 µM) with non-cytotoxic activity against the MCF-10A. Other compounds exhibited relatively moderate-to-weak cytotoxic activities. Hence, three compounds; **6e**, **9f**, and **6k**, were chosen to be studied for their potential molecular targets and ability to induce apoptosis in MCF-7 cells (Figure 4).

#### 2.2.2. Potential EGFR/HER2 Kinase Inhibitory Assay

To further elucidate the mechanisms behind the cytotoxic effects on MCF-7 cells, compounds **3c**, **6e**, **6k**, **6f** and **9f** were tested for their ability to inhibit EGFR and HER2. As seen in Table 2, the tested compounds exhibited promising dual EGFR/HER2 inhibition activities. Remarkably, compounds **6k** and **6e** had IC_50_ values of 0.014 and 0.009 µM against EGFR and IC_50_ values of 0.027 and 0.013 µM against HER2, respectively, in a comparable way to Lapatinib. Due to their potent cytotoxic and EGFR/HER2 inhibitory effects, compounds **6k** and **6e** merited investigation for their ability to induce apoptosis.

#### 2.2.3. Apoptotic Investigation

##### Annexin V/PI Staining with Cell Cycle Analysis

**Figure 5:**
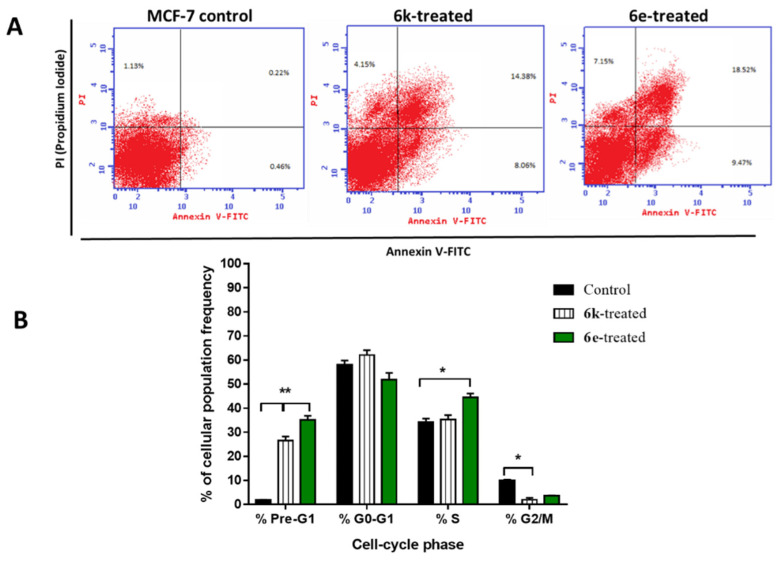
(**A**) Cryptographs of annexin-V/Propidium Iodide staining of untreated and **6k** and **6e**-treated MCf-7 cells with the IC_50_ values, 48 h, Q1-UL (necrosis, AV−/PI+), Q1-UR (late apoptotic cells, AV+/PI+), Q1-LL (normal cells, AV−/PI−), Q1-LR (early apoptotic cells, AV+/PI−), (**B**) Bar representation of the percentage of cell population at each cell cycle G0-G1, S, G2/M, and Pre-G1 using DNA content–flow cytometry-aided cell cycle analysis. The data demonstrated is the mean of 3-independent experimental runs (mean ± SD). ** *p* < 0.001 and * *p* < 0.05 compared to control using an unpaired *t* test by GraphPad prism.

Flow cytometric analysis of Annexin V/PI staining was used to examine apoptotic cell death in untreated and treated MCF-7 cells to determine the apoptotic activity of the two promising compounds, **6k** and **6e** (IC_50_ = 8.02, 7.21 µM, 48 h). As seen in Figure 5A, compounds **6k,** and **6e** significantly stimulated apoptotic breast cancer cell death with 22.44% (14.38% for late apoptosis, 8.06% for early apoptosis) and 27.99% (18.52% for late apoptosis, 9.47% early apoptosis), respectively, compared to 0.68% in the control.

Cytotoxic substances were tested using DNA flow cytometry to determine what percentage of cells were in each cell cycle following treatment. Figure 5B, the percentage of cells in the S phase increased from 34.18 to 44.52 % after treatment with compound **6e**, indicating that compound **6e** induced cell cycle arrest in the S phase, while the percentage of cells in the G2/M phase was reduced by 2.01% after treatment with compound **6k**, compared to the control (9.7%). This result indicates that compound **6k** induced cell cycle arrest in the G2/M-phase. By comparison, other phases were not significantly changed in either treatment.

##### RT-PCR

**Figure 6:**
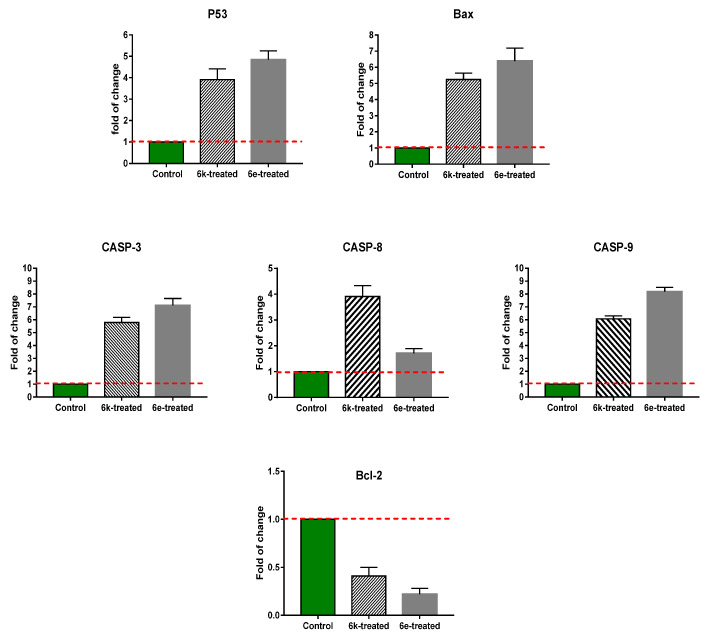
Quantitative RT-PCR results analysis of the apoptosis-related genes; P53, Bax, Caspases 3, 8, 9, and Bcl-2, respectively in MCF-7 cells treated with **6e** and **6k** with the IC_50_ values, 48 h. The data illustrated are the average of 3 independent experimental runs (mean ± SD). Data were normalized using β-actin as a housekeeping gene. Red dashed line represents the gene expression for untreated control.

Further validation of the apoptosis-inducing activities of both tested compounds **6k** and **6e** in MCF-7 cells, the gene expression levels of apoptosis-related genes in both untreated and treated MCF-7 cells through the RT-PCR. As seen in Figure 6, compounds **6k** and **6e** treatments increased P53 level by 3.91- and 4.8-fold, Bax level by 5.24- and 6.39-fold, caspase 3 level by 5.79- and 7.12-fold, caspase 8 level by 3.91- and 1.71-fold, caspase 9 level by 6.07- and 8.19-fold, while both compound treatments decreased Bcl-2 level “as the anti-apoptotic gene” by 0.41- and 0.32-fold compared to untreated control.

P53 is a tumor suppressor gene that is essential for apoptosis. The activation of caspases 3 and 9 and the decreased expression of the Bcl-2 gene may cause p53 apoptosis. The results showed the intrinsic apoptotic pathway through activation of P53, Bax, and caspases 3 and 9 levels. Further, the treatment-induced upregulation of caspase 8 gene promotes an extrinsic apoptotic pathway in MCF-7 cells. As a result, our RT-PCR findings corroborated those of previous studies, showing that the apoptotic pathway is highlighted by the upregulation of proapoptotic genes and the downregulation of anti-apoptotic genes (Table 3).

#### 2.2.4. In Vivo Studies

**Figure 7:**
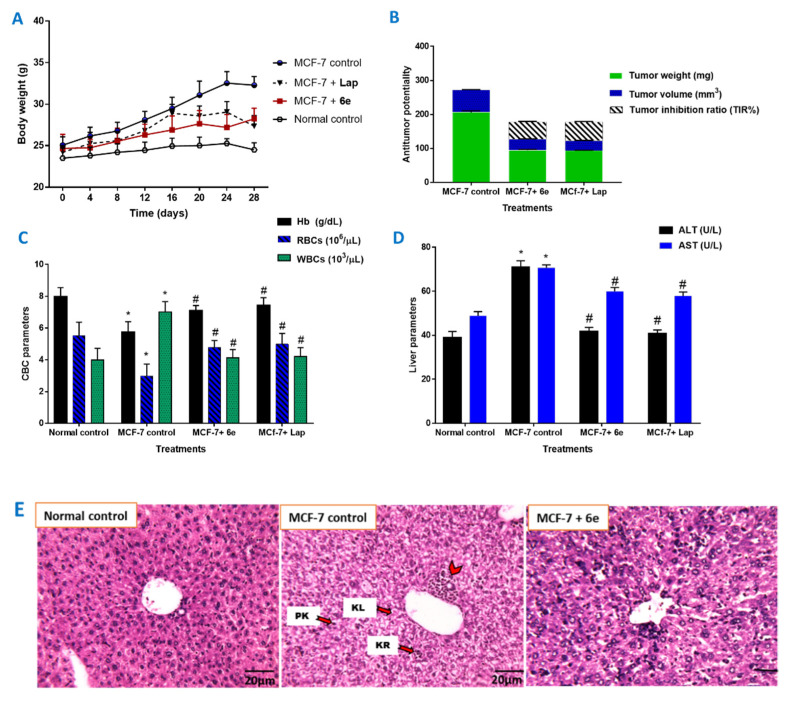
Assessment of in vivo results (Xenograft model) (**A**) bodyweight increase during experiment duration, (**B**) antitumor potentiality of solid mass volume, mass, and tumor inhibition ratio (TIR%), (**C**) Hematological assays of Hemoglobin, Red blood cells, and white blood cells, (**D**) biochemical assays of ALT, AST activities, and (**E**) histopathological examinations of liver tissues in different treated groups; Normal control, MCF-7 control, MCF-7 + **6e**, and MCF-7 + Lapatinib. (Arrowhead) inflammatory infiltration, (KL) karyolysis, (KR) Karyorrhexis, (PK) Pyknosis. Results are expressed as mean ± SD of three independent trials. * (*p* ≤ 0.05) significantly different between MCF-7 control and normal control, while # (*p* ≤ 0.05) is significantly different between treated groups compared to MCF-7 control.

For evaluation of the anticancer activity of compound **6e**, mice, MCF-7 cells (Xenograft model) were treated with compounds **6e** and lapatinib at a dose (10 mg/ kg BW, IP). As seen in Figure 7A, an increase in the mice weight of the MCF-7 control mice reached 32.28 g, compared to 24.5 g in normal mice, while treatment with compound **6e** and lapatinib reduced the mice weight to 28.23 g and 27.35 g, respectively. The reduction in the mice’s weight was consequently related to a decrease in the tumor mass; tumor volume decreased from 89.8 mg to 28.4 mm^3^, respectively, upon treatment with compound **6e**. Hence, compound **6e** treatment enhanced tumor inhibition ratio by 52.46% compared to 50.53% in Lapatinib treatment Figure 7B.

As seen in Figure 7C, the hemoglobin and red blood cell concentrations dropped dropped dramatically to 5.7 (g/dL) and 2.9 (10^6^/L), respectively. Conversely, white blood cell count was elevated to 7.0 (10^3^/L) from normal control levels. Routine consequences of tumor proliferation are reduced levels of RBC, hemoglobin, and increased WBC counts [63,64]. CBC was nearly maintained at normal levels following treatment with compound **6e**, where Hb (7.0 g/dL) and RBC’s count (4.8 10^6^/μL) were raised, and WBCs count was lowered (4.12 10^3^/μL). While Lapatinib-treatment increased Hb to 7.43 g/dL, RBC’s count to 4.99 10^6^/μL and decreased WBC’s count to 4.21 10^3^/μL compared to MCF-7 group.

As seen in Figure 7D, liver enzymes of ALT, AST were significantly elevated to 70.9, 70.65 (U/L), respectively in MCF-7 control mice compared to normal mice at 38.9 and 48.9 (U/L), respectively, caused by tumor inoculation-related hepatocellular damage, while liver enzymes were markedly mitigated by treatment with compound **6e**. Liver enzymes were reduced to 41.8, 59.9 U/L, respectively, and this significantly reduced the toxicity to the liver caused by the cancer. While Lapatinib treatment decreased ALT to 40.8 U/L, AST fell to 57.7 U/L compared to the MCF-7 group

In agreement with the amelioration of biochemical parameters, histopathological examinations Figure 7E in liver sections in **6e**-treated mice showed normal nuclei morphology in most of the cells with slight hydrophobic degeneration compared to MCF-7 control mice, which showed chronic inflammation (red arrow), and hepatocytes showing hydropic degeneration (arrowheads).

Taken together, amelioration in hematological, and biochemical results of compound **6e** treatment agreed with the in vitro ones of cytotoxicity, EGFR/HER2 targeting, and apoptosis induction.

### 2.3. Computational Studies

#### 2.3.1. Molecular Docking

**Figure 8:**
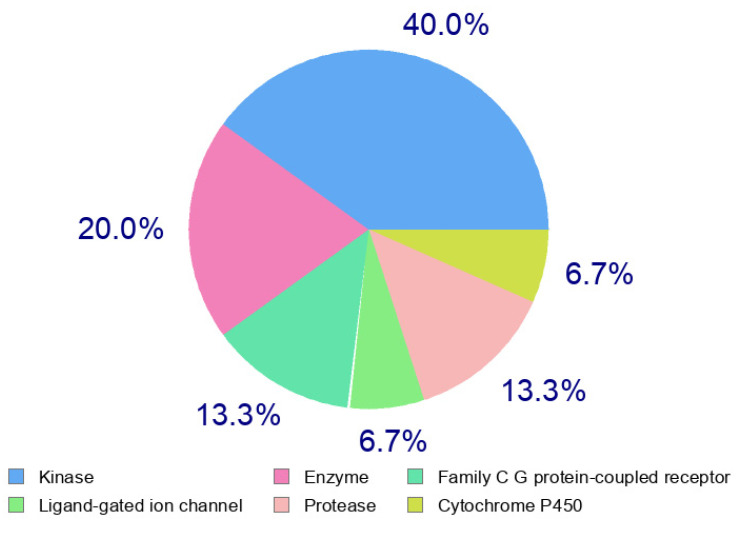
Target prediction for the proposed compounds using Swiss Target Prediction online tool.

To discover the most appropriate drug target for the proposed compounds, SwissTargetPrediction [65] was used. The studied compounds revealed that kinase proteins were the first class to be targeted, with a probability of 40% (Figure 8).

**Figure 9:**
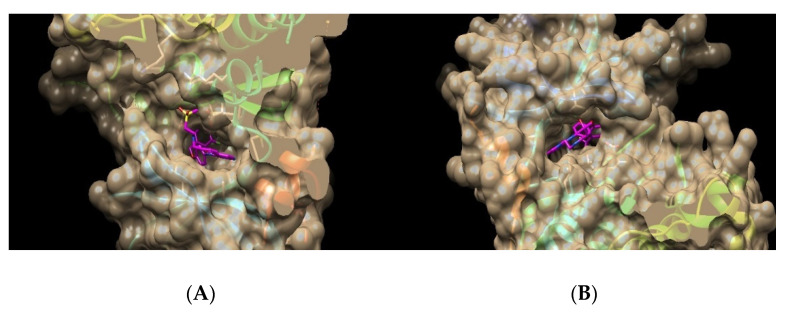
Co-crystallized ligands interactions: (**A**) Lapatinib in EGFR active binding site, (**B**) TAK285 interactions in HER2 active binding site.

Molecular docking was carried out to estimate the interaction of the designed hybrid compounds with EGFR/HER2 kinase domains to rationalize their biological activity, and to reveal their probable binding pattern. Crystal structures were downloaded (PDB codes: 1XKK [66] and 3RCD [67]) for EGFR and HER2, respectively. Initially, autodocking of the co-crystallized ligands in EGFR and HER2 active sites was performed to validate the docking method. In the EGFR active site, the docking poses of lapatinib formed the key interactions with the active site through H-bond formation with amino acid residues Met793, Asp800, and Leu788 at distance of 3.17, 3.3, and 3.19 Å, respectively. In addition, arene H interactions with Leu844 and Leu718 were formed as illustrated in Figure 9A. As for HER2, the docking pose of TAK-285 produced the key interactions with the active site; it interacts through an H bond with amino acid residues Asp863, Gly727, and 2 H bonds with Met801 at distance of 3.22, 3.18, 3.02, 3.82 Å, respectively. Besides, π–H interactions with Phe864, Lys753, Leu852, and Leu800 were formed as seen in Figure 9B. The method validation was demonstrated by the resulted small RMSD values between the docked and the co-crystallized ligand poses in EGFR (0.917 Å, Figure S1) and HER2 (0.625 Å, Figure S2).

**Figure 10:**
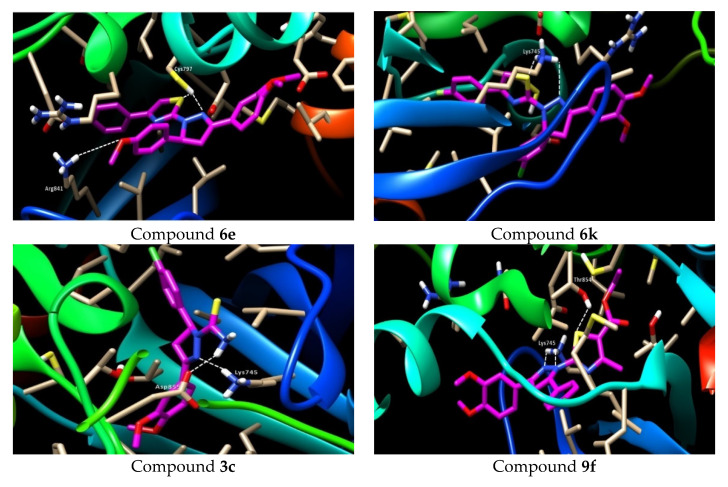
Binding interactions of the promising derivatives (**6e**, **6k**, **3c**, and **9f**) in the EGFR receptor (1XKK) showing ligands (magenta) and H bonds (white-dashed).

**Figure 11:**
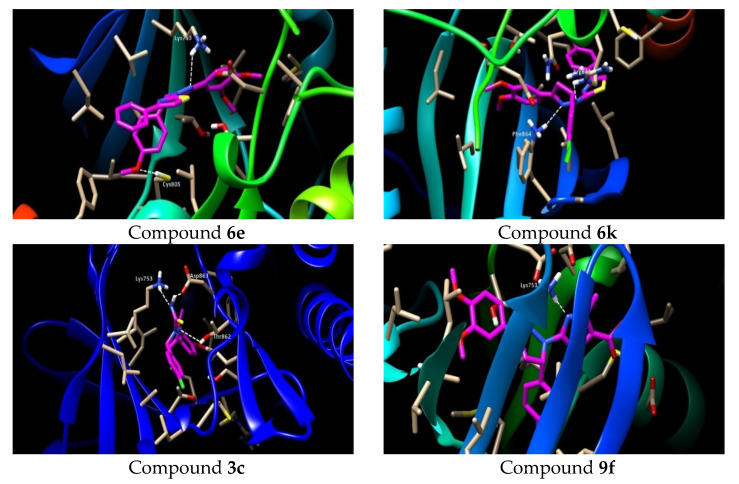
Binding interactions of the promising derivatives (**6e**, **6k**, **3c**, and **9f**) in the HER2 receptor (3RCD) showing ligands (magenta) and H bonds (white-dashed).

The newly synthesized thiazole–pyrazoline hybrids showed comparable binding patterns in both tyrosine kinases. Binding features of compounds **6e**, **6k**, **3c**, and **9f** were studied in more detail and compared to the docked co-crystallized inhibitors of either EGFR (Figure 10) and HER2 (Figure 11). From binding modes analyses, we can conclude that:

(1) Compound **6e** inside the binding site of the EGFR receptor (binding free energy = −13.05 kcal/mol) formed H bonds with Met793, Pro749, Arg841, Cys797, Gly796, and Leu792 at distances of 4.08, 2.69, 2.45, 2.03, 3.36 and 4.23 Å, respectively. Besides, π–H bonds with Lys745 and Val726 were formed. For HER2, compound **6e** (binding free energy = −11.92 kcal/mol) showed H bonds with Cys805, Lys753, Thr862, Val734 and Asp863 at distance of 2.09, 2.31, 4.3, 3.99 and 3.32 Å, respectively. In addition, a π–H bond with Val734 was formed.
(2) Compound **6k** inside the binding site of EGFR receptor (binding free energy = −13.04 kcal/mol) achieved an H bond with Met793, Asp855, and Lys745 at a distance of 2.85, 3.05, 2.45 Å, respectively. Π–H bond with Val726, Leu718, Arg841, and Gly719 were formed. Furthermore, inside the binding site of HER2 receptor, compound **6k** (binding free energy = −13.11 kcal/mol) established H bonds with Met801, Arg849, Phe864, Val734, and Asp863 at distance of 2.76, 2.13, 3.54, 4.26 and 3.1 Å, respectively. Besides π–H bonds with Cys805, Lys753 and Leu852 were noticed.
(3) Compound **3c** inside the binding site of EGFR receptor (binding free energy = −13.36 kcal/mol) achieved H bonds with Met793, Asp855, Lys745, and Gly796 at distance of 3.04, 2.99, 3.36, and 3.56 Å, in addition to the formation of π–H bonds with Leu718 and Phe856. Moreover, regarding the binding pocket of Her2 receptor (binding free energy = −11.3 kcal/mol), compound **3c** formed H bonds with Lys753, Asp863, and Thr862 at a distance of 3.06, 2.77, and 4.23 Å, respectively and one π–H bond with Leu785.
(4) For compound **9f** inside the EGFR receptor binding site (binding free energy = −13.93 kcal/mol), H bonds with Met793, Asp855, Thr854, Lys745, Val726, and Met1002 were formed at distance of 3.05, 3.2, 2.18, 2.61, 3.55, and 3.8 Å, respectively, in addition to π–H bonds with Lys745 and Val726. On the other hand, compound **9f** binding within the binding pocket of HER2 receptor (binding free energy = −11.85 kcal/mol) showed H bonds with Met801, Lys753, and Leu852 at distance of 3.01, 2.69, and 4.09 Å, respectively, besides one π–H bond with Val734.

#### 2.3.2. In Silico Physicochemical Descriptors, Pharmacokinetic Properties and Bioactivity Prediction

Different parameters were measured to explore drug-likeness properties (Table S1) of the target thiazolyl–pyrazoline compounds, all of which obey Veber’s rule and Lipinski’s rule and may meet the criteria for orally active drugs, except for **6l** which has 2 violations of the Lipinski rule. All compounds were predicted to have a clogP value in the range of 2.00–6.00, TPSA ≤ 110.72 Å^2^, rotatable bonds (RB) ≤ 9, H-bond acceptor moieties (HBA) ≤ 7, H-bond donor moieties (HBD) ≤ 1 and molar refractivity (MR) ≤ 147.86. Compound solubility was in the range of poorly soluble to moderately soluble, except for compounds **3a** and **3b** which were soluble. Bioavailability radar (Figures S3 and S4) indicated that molecules **3a**–**c** and **9a**–**e** are expected to be orally bioavailable.

In silico ADME prediction of pharmacokinetic properties was investigated for the target compounds (Table S2). All compounds showed very high HIA values, ranging from 97.5–99.6%, indicating very high GI absorption. All compounds exerted a strong bound effect on plasma protein (binding values of 87.45–93.09%). All compounds are not blood-brain barrier permeant and have skin *p* values from −2.35 to −3.43. On inspecting the boiled-egg model (Figure S5), all compounds were found to be in the white region indicating their proper GI absorption.

An in silico phase I metabolism study indicated that all compounds could inhibit CYP2C9, and that all compounds have CYP3A4-inhibiting activity except 6l. None of the thiazolyl–pyrazoline hybrids could inhibit CYP1A2.

Toxic properties such as irritant, mutagenic, and reproductive effects using were predicted Osiris server (Table S3). All compounds have no mutagenic, irritant, or reproductive fragments except for **3a**, **3b**, and **3c**, which have reproductive effects due to the 1-ethyl hydrazine-1-carbothioamide fragment.

#### 2.3.3. Structure–Activity Relationship (SAR) Study

**Figure 12:**
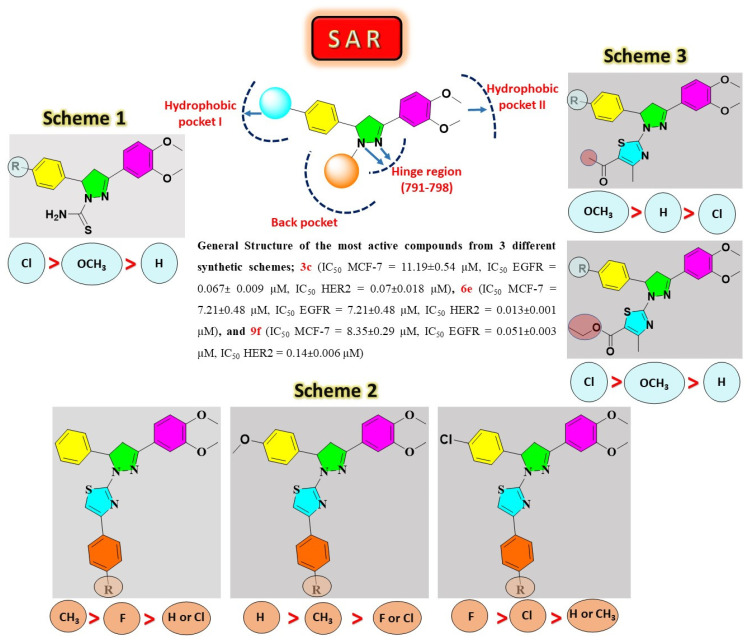
SAR of the newly synthesized compounds **3a**–**c** (Scheme 1), **6a**–**l** (Scheme 2) and **9a**–**f** (Scheme 3).

SAR of the newly synthesized compounds **3a**–**c** (Chart 1), **6a**–**l** (Chart 2) and **9a**–**f** (Chart 3) was determined depending on the biological results as well as the docking binding free energy values. By superimposition of the most active compounds; **3c** (IC_50_ MCF-7 = 11.19 ± 0.54 µM, IC_50_ EGFR = 0.067 ± 0.009 µM, IC_50_ HER2 = 0.07 ± 0.018 µM), **6e** (IC_50_ MCF-7 = 7.21 ± 0.48 µM, IC_50_ EGFR = 7.21 ± 0.48 µM, IC_50_ HER2 = 0.013 ± 0.001 µM), and **9f** (IC_50_ MCF-7 = 8.35 ± 0.29 µM, IC_50_ EGFR = 0.051 ± 0.003 µM, IC_50_ HER2 = 0.14 ± 0.006 µM), we conclude that all of them formed the necessary interactions in the Hinge region of the kinase domain due to the presence of the pyrazoline moiety in their structures. 3,4-dimethoxyphenyl moiety increases the activity due to the interactions formed in the Hydrophobic II pocket. Different substituents, oriented towards the Back pocket, showed different activities depending on the other moieties in the structure that can embed in the Hydrophobic I pocket (Figure 12).

## 3. Materials and Methods

### 3.1. Chemistry

NMR spectra were recorded by a Bruker spectrometer. ^1^H NMR spectra were run at 400 MHz, and ^13^C NMR spectra were run at 100 MHz in deuterated dimethylsulfoxide (DMSO-d_6_). Chemical shifts (*δ_H_* and *δ_C_*) were reported relative to the solvent (DMSO-d_6_). Infrared spectra were recorded on a Shimadzu FT-IR 8400S spectrophotometer as KBr disks at the faculty of science, Sohag University. Elemental analysis was performed using FLASH 2000 CHNS/O analyzer, at the Regional Center for Microbiology and Biotechnology, Al-Azhar University. The reaction progress was monitored using thin-layer chromatography (TLC)-precoated silica gel G plates, and the formation of products was visualized by irradiation with UV light (254 nm). Melting points were determined using Stuart Scientific apparatus and were uncorrected. All the chemicals were purchased from Alpha Aesar and Sigma-Aldrich (Figures S6–S79).

#### 3.1.1. Synthesis of 3-(4-Aryl)-1-(3,4-dimethoxyphenyl)prop-2-en-1-one (**2a**–**c**)

A solution of dimethoxy acetophenone (1.08 g, 6.0 mmol) in absolute ethanol (30 mL) was stirred with an equimolar weight of the appropriate aromatic aldehyde 1a-c (6.0 mmol), NaOH (2 g, 50 mmol) in water (15 mL) was added within 30 min. The reaction mixture was stirred for 5 h at room temperature. The reaction progress was monitored using TLC and methylene chloride/methanol (9:1) system until the completion of the reaction. Then, the resulting precipitate was filtered and washed with ethanol, and then recrystallized from absolute ethanol to give a yield of 95–98%.

#### 3.1.2. Synthesis of 3-(3,4-Dimethoxyphenyl)-5-(4-aryl)-4,5-dihydro-1H-pyrazole-1-carbothioamide (**3a**–**c**)

A mixture of 3-(4-aryl)-1-(3,4-dimethoxyphenyl)prop-2-en-1-one (2a-c) (20 mmol), thiosemicarbazide (1.8 g, 20 mmol) and NaOH (36 mmol) in ethanol (25 mL) was refluxed for 12 h. The formed precipitate was filtered on hot, washed with ethanol and then recrystallized from EtOH/DMF to furnish carbothioamides **3a**–**c**, respectively.

##### Synthesis of 3-(3,4-Dimethoxyphenyl)-5-phenyl-4,5-dihydro-1H-pyrazole-1-carbothioamide (**3a**)

White powder, 79% yield; m.p. 188–190 °C; IR (KBr) *v*max cm^−1^ 3422, 32874 (NH_2_), 3070 (C–H Str. of alkene), 2958 (C–H Str. of alkane), 1607 (C=N), 1596 (C=C); ^1^H NMR (DMSO-d_6_) *δ* 8.01 (br. s, 2H, NH_2_), 7.64 (s, 1H, Ar), 7.33–7.22 (m, 4H, Ar), 7.14 (d, *J* = 7.2 Hz, 2H, Ar), 6.98 (d, *J* = 8.4 Hz, 1H, Ar), 5.93 (dd, *J* = 11.2, 2.4 Hz, 1H, H5 of pyrazoline), 3.83 (dd, *J* = 18, 11.2 Hz, 1H, H_b_4 of pyrazoline), 3.84 (s, 3H, 3-OCH_3_), 3.79 (s, 3H, 4-OCH_3_), 3.13 (dd, *J* = 18, 2.4 Hz, 1H, H_a_4 of pyrazoline); ^13^C NMR (DMSO-d_6_) *δ* 42.94 (C4 pyrazoline), 56.07 (4-OCH_3_), 56.14 (3-OCH_3_), 63.26 (C5 pyrazoline), 109.91 (Ar-C), 111.67 (Ar-C), 121.61 (Ar-C), 123.92 (Ar-C), 125.75 (2x C Ar), 127.35 (Ar-C), 128.95 (2x C Ar), 143.53 (Ar-C), 149.34 (Ar-C), 151.58 (Ar-C), 155.42 (Ar-C), 176.2 (CS). MS (*m*/*z*) (%) 341.27 (M^+^); Anal. Calcd. For: C_18_H_19_N_3_O_2_S (341.43): C, 63.32; H, 5.61; N, 12.31; S, 9.39; Found: C, 63.59; H, 5.78; N, 12.52, S, 9.48.

##### Synthesis of 3-(3,4-Dimethoxyphenyl)-5-(4-methoxyphenyl)-4,5-dihydro-1H-pyrazole-1-carbothioamide (**3b**)

White powder, 80% yield; m.p. 221–223 °C; IR (KBr) *ν*max/cm^−1^ 3395, 3259 (NH_2_), 2961 (C–H Str. of alkane), 1603 (C=N), 1569 (C=C); ^1^H NMR (DMSO-d_6_) *δ* 7.98 (br. s, 2H, NH_2_ exchangeable D_2_O), 7.65 (s, 1H, Ar), 7.25 (d, *J* = 8.0 Hz, 1H, Ar), 7.07 (d, *J* = 8.3 Hz, 2H, Ar), 6.98 (d, *J* = 8.3 Hz, 1H, Ar), 6.87 (d, *J* = 8.1 Hz, 2H, Ar), 5.88 (dd, *J* = 11.2, 3.3 Hz, 1H, H5 pyrazoline), 3.78 (dd, *J* = 17.9, 11.2 Hz, 1H, H_b_4 pyrazoline), 3.85 (s, 3H, 3-OCH_3_), 3.80 (s, 3H, 4-OCH_3_), 3.67 (s, 3H, 4-OCH_3_), 3.12 (dd, *J* = 17.9, 3.3 Hz, 1H, H_a_4 pyrazoline); ^13^C NMR (DMSO-d_6_) *δ* 42.91 (C4 pyrazoline), 55.49 (4-OCH_3_), 56.05 (4-OCH_3_), 56.13 (3-OCH_3_), 62.76 (C5 pyrazoline), 109.87 (Ar-C), 111.65 (Ar-C), 114.26 (2x C Ar), 121.59 (Ar-C), 123.98 (Ar-C), 127.10 (2x C Ar), 135.57 (Ar-C), 149.35 (Ar-C), 151.57 (Ar-C), 155.47 (Ar-C), 158.65 (Ar-C), 176.2 (CS). MS (*m*/*z*) (%) 370.3 [M − 1]^−^; Anal. Calcd. For: C_19_H_21_N_3_O_3_S (371.5): C, 61.44; H, 5.7; N, 11.31; S, 8.63; Found: C, 61.72; H, 5.86; N, 11.58; S, 8.75.

##### Synthesis of 5-(4-Chlorophenyl)-3-(3,4-dimethoxyphenyl)-4,5-dihydro-1H-pyrazole-1-carbothioamide (**3c**)

Yellowish white powder, 60% yield; m.p. 230–232 °C; IR (KBr) νmax/cm^−1^ 3393, 3265 (NH_2_), 3010 (C–H Str. of alkene), 2936 (C–H Str. of alkane), 1604 ( C=N), 1572 (C=C); ^1^H NMR (DMSO-d_6_) *δ* 8.04 (br. s, 2H, NH_2_), 7.64 (d, *J* = 2 Hz,1H, Ar), 7.37 (d, *J* = 8.4 Hz, 2H, Ar), 7.25 (dd, *J* = 8.4, 2 Hz, 1H, Ar), 7.16 (d, *J* = 8.4 Hz, 2H, Ar) 6.98 (d, *J* = 8.4 Hz, 1H, Ar), 5.92 (dd, *J* = 11.2, 3.5 Hz, 1H, H5 pyrazoline), 3.75 (dd, *J* = 18, 11.2 Hz, 1H, H_b_4 pyrazoline), 3.84 (s, 3H, 3-OCH_3_), 3.79 (s, 3H, 4-OCH_3_), 3.14 (dd, *J* = 18, 3.5 Hz, 1H, H_a_4 pyrazoline); ^13^C NMR (DMSO-d_6_) *δ* 42.73 (C4 pyrazoline), 56.07 (4-OCH_3_), 56.14 (3-OCH_3_), 62.72 (C5 pyrazoline), 109.93 (Ar-C), 111.65 (Ar-C), 121.65 (Ar-C), 123.81 (Ar-C), 127.79 (2x C Ar), 128.92 (2x C Ar), 131.86 (Ar-C), 142.51 (Ar-C), 149.33 (Ar-C), 151.62 (Ar-C), 155.37 (Ar-C), 176.17 (CS). MS (APCI^+^) *m*/*z* (%) 376.5 [M + 1]^+^; Anal. Calcd. For: C_18_H_18_N_3_O_2_SCl (375.87): C, 57.52; H, 4.83; N, 11.18; S, 8.53; Found: C, 57.65; H, 4.97; N, 11.45; S, 8.70.

#### 3.1.3. Synthesis of 2-(3-(3,4-Dimethoxyphenyl)-5-(aryl)-4,5-dihydro-1H-pyrazol-1-yl)-4-(aryl^1^) Thiazole

The appropriate amount of phenacyl bromide **5a**–**d** (0.6 mmol) was added to a solution of pyrazoline derivatives **3a**–**c** (0.6 mmol) in absolute ethanol (15 mL), before it was refluxed at 90 °C for 5 h. After cooling, the obtained solid was filtered off, washed with ethanol, and recrystallized from EtOH/DMF to give compounds **6a**–**l**.

##### Synthesis of 2-(3-(3,4-Dimethoxyphenyl)-5-phenyl-4,5-dihydro-1H-pyrazol-1-yl)-4-phenylthiazole (**6a**)

Yellowish white powder, 55% yield; m.p. 202–205 °C; IR (KBr) νmax/cm^−1^ 3108 (C–H Str. of alkene), 2959 (C–H Str. of alkane), 1600 (C=N), 1543, 1543 (Ar C=C); ^1^H NMR (DMSO-d_6_) *δ* 7.71 (d, *J* = 7.2 Hz, 2H, Ar), 7.43–7.23 (m, 11H, Ar), 7.05 (d, *J* = 8.5 Hz, 1H, Ar), 5.67 (dd, *J* = 12, 6.4 Hz, 1H, H5 pyrazoline), 4.05 (dd, *J* = 18, 12 Hz, 1H, H_b_4 pyrazoline), 3.84 (s, 3H, Hs of 3-OCH_3_), 3.82 (s, 3H, 4-OCH_3_), 3.36 (dd, *J* = 18, 6.4 Hz, 1H, H_a_4 pyrazoline); ^13^C NMR (DMSO-d_6_) *δ* 43.87 (C4 pyrazoline), 56.02 (4-OCH_3_), 56.1 (3-OCH_3_), 64.56 (C5 pyrazoline), 104.53 (Ar-C), 109.51 (Ar-C), 112.10 (Ar-C), 120.63 (Ar-C), 124.06 (Ar-C), 125.93 (2x C Ar), 127 (2x C Ar), 127.95 (2x C Ar), 128.95 (2x C Ar), 129.03 (2x C Ar), 134.96 (Ar-C), 142.49 (Ar-C), 149.29 (Ar-C), 150.87 (Ar-C), 151.09 (Ar-C), 153.39 (Ar-C), 164.90 (Ar-C). MS (ESI^+^) *m*/*z* (%) 442.5[M + H]^+^; Anal. Calcd. For: C_26_H_23_N_3_O_2_S (441.55): C, 70.73; H, 5.25; N, 9.52; S, 7.26; Found: C, 70.95; H, 5.41; N, 9.80; S, 7.43.

##### Synthesis of 2-(3-(3,4-Dimethoxyphenyl)-5-phenyl-4,5-dihydro-1H-pyrazol-1-yl)-4-(p-tolyl)thiazole (**6b**)

White powder, 77.6% yield; m.p. 210–215 °C; IR (KBr) νmax/cm^−1^ 2923 (C–H Str. of alkane), 1599 (C=N), 1549, 1500 (C=C). ^1^H NMR (DMSO-d_6_) *δ* 7.61 (d, *J* = 8 Hz, 2H, Ar), 7.43–7.25 (m, 7H, Ar), 7.21 (s, 1H, H of thiazole), 7.14 (d, *J* = 7.8 Hz, 2H, Ar), 7.03 (d, *J* = 8.4 Hz, 1H, Ar), 5.64 (dd, *J* = 14.8, 6.4 Hz, 1H, H5 pyrazoline), 4.03 (dd, *J* = 18.6, 14.8 Hz, 1H, H_b_4 pyrazoline), 3.84 (s, 3H, 3-OCH_3_), 3.81 (s, 3H, 4-OCH_3_), 3.30 (dd, *J* = 18.6, 6.4 Hz, 1H, H_a_4 pyrazoline), 2.28 (s, 3H, 4-CH_3_); ^13^C NMR (DMSO-d_6_) *δ* 21.24 (4-CH_3_), 43.81 (C4 pyrazoline), 55.99 (4-OCH_3_), 56.07 (3-OCH_3_), 64.57 (C5 pyrazoline), 103.59 (Ar-C), 109.47 (Ar-C), 112.04 (Ar-C), 120.59 (Ar-C), 124.08 (Ar-C), 125.90 (2 x C Ar), 127.02 (2 x C Ar), 127.91 (Ar-C), 128.99 (2 x C Ar), 129.50 (2 x C Ar), 132.36 (Ar-C), 137.21 (Ar-C), 142.50 (Ar-C), 149.29 (Ar-C), 150.98 (Ar-C), 151.08 (Ar-C), 153.24 (Ar-C), 164.84 (Ar-C); MS *m*/*z* (%) 455.27 (M^+^); Anal. Calcd. For: C_27_H_25_N_3_O_2_S (455.58): C, 71.18; H, 5.53; N, 9.22; S, 7.04; Found: C, 71.42; H, 5.67; N, 9.49; S, 7.18.

##### Synthesis of 2-(3-(3,4-Dimethoxyphenyl)-5-phenyl-4,5-dihydro-1H-pyrazol-1-yl)-4-(4-fluorophenyl)thiazole (**6c**)

White powder, 76% yield; m.p. 135–140 °C; IR (KBr) νmax/cm^−1^ 1601(C=N), 1551, 1501(C=C); ^1^H NMR (DMSO-d_6_) *δ* 7.75 (td, *J =* 6, 2.8 Hz, 2H, 4-FC_6_H_4_), 7.41–7.25 (m, 8H, Ar), 7.18 (t, *J =* 8.9 Hz, 2H, 4- FC_6_H_4_), 7.04 (d, *J* = 8.8 Hz, 1H, Ar), 5.65 (dd, *J* = 11.8, 6.6 Hz, 1H, H5 pyrazoline), 4.04 (dd, *J* = 17.8, 11.8 Hz, 1H, H_b_4 pyrazoline), 3.84 (s, 3H, 3-OCH_3_), 3.82 (s, 3H, 4-OCH_3_), 3.30 (dd, *J* = 17.8, 6.6 Hz, 1H, H_a_4 pyrazoline); ^13^C NMR (DMSO-d_6_) *δ* 43.89 (C4 pyrazoline), 56.02 (4-OCH_3_), 56.10 (3-OCH_3_), 64.54 (C5 pyrazoline), 104.26 (Ar-C), 109.52 (Ar-C), 112.08 (Ar-C), 115.68 (Ar-C), 115.89 (Ar-C), 120.64 (Ar-C), 124.03 (Ar-C), 126.98 (2 x C Ar), 127.85 (Ar-C), 127.94 (Ar-C), 129.04 (2 x C Ar), 131.61 (Ar-C), 142.45 (Ar-C), 149.29 (Ar-C), 149.83 (Ar-C), 151.11 (Ar-C), 153.47 (Ar-C), 160.80 (Ar-C), 163.23 (Ar-C), 164.99 (Ar-C). MS (ESI^+^) *m*/*z* (%) 460.2 [M + 1]^+^; Anal. Calcd. For: C_26_H_22_N_3_O_2_SF (459.54): C, 67.96; H, 4.83; N, 9.14; S, 6.98; Found: C, 67.78; H, 5.01; N, 9.42; S, 6.85.

##### Synthesis of 4-(4-Chlorophenyl)-2-(3-(3,4-dimethoxyphenyl)-5-phenyl-4,5-dihydro-1H-pyrazol-1-yl)thiazole (**6d**)

White powder, 81.3% yield; m.p. 195–198 °C; IR (KBr) νmax/cm^−1^ 2935 (C–H Str. of alkane), 1600 (C=N), 1549 (C=C); ^1^H NMR (DMSO-d_6_) *δ* 7.72 (d, *J* = 8.4 Hz, 2H, Ar), 7.41–7.25 (m, 10H, Ar), 7.04 (d, *J* = 8.4 Hz, 1H, Ar), 5.66 (dd, *J* = 12, 6.4 Hz, 1H, H5 pyrazoline), 4.04 (dd, *J* = 17.6, 12 Hz, 1H, H_b_4 pyrazoline), 3.84 (s, 3H, 4-OCH_3_), 3.82 (s, 3H, 3-OCH_3_), 3.35 (dd, *J* = 17.6, 6.4 Hz, 1H, H_a_4 pyrazoline); ^13^C NMR (DMSO-d_6_) *δ* 43.43 (C4 pyrazoline), 55.55 (-OCH_3_), 55.64 (-OCH_3_), 64.03 (C5 pyrazoline), 104.88 (Ar-C), 109.07 (Ar-C), 111.61 (Ar-C), 120.19 (Ar-C), 123.54 (Ar-C), 126.52(2 x C Ar), 127.15(2 x C Ar), 127.49 (Ar-C), 128.51(2 x C Ar), 128.59 (2 x C Ar), 131.88 (Ar-C), 133.37 (Ar-C), 141.93 (Ar-C), 148.83 (Ar-C), 149.17 (Ar-C), 150.67 (Ar-C), 153.11 (Ar-C), 164.53 (Ar-C). MS (APCI^+^) *m*/*z* (%) 476.6 (M + 1)^+^; Anal. Calcd. For: C_26_H_22_N_3_O_2_SCl (476): C, 65.61; H, 4.66; N, 8.83; S, 6.74; Found: C, 65.87; H, 4.80; N, 9.09; S, 6.85.

##### Synthesis of 2-(3-(3,4-Dimethoxyphenyl)-5-(4-methoxyphenyl)-4,5-dihydro-1H-pyrazol-1-yl)-4-phenylthiazole (**6e**)

Off-white powder, 36.6% yield; m.p. 168–170 °C; IR (KBr) νmax/cm^−1^ 1603, 1535; ^1^H NMR (DMSO-d_6_) *δ* 7.75 (d, *J* = 7.6 Hz, 2H, Ar), 7.39–7.24 (m, 8H, Ar), 7.05 (d, *J* = 8 Hz, 1H, Ar), 6.93 (d, *J* = 8.4 Hz, 2H, Ar), 5.62 (dd, *J* = 12, 6 Hz, 1H, H5 pyrazoline), 4.00 (dd, *J* = 17.6, 12 Hz, 1H, H_b_4 pyrazoline), 3.85 (s, 3H, 3-OCH_3_), 3.83 (s, 3H, 4-OCH_3_), 3.72 (s, 3H, 4-OCH_3_), 3.34 (dd, *J* = 17.6, 6 Hz, 1H, H_a_4 pyrazoline); ^13^C NMR (DMSO-d_6_) *δ* 43.70 (C4 pyrazoline), 55.51 (4-OCH_3_), 56.01 (4-OCH_3_), 56.09 (3-OCH_3_), 64.03 (C5 pyrazoline), 104.41 (Ar-C), 109.48 (Ar-C), 112.07 (Ar-C), 114.34 (2 x C Ar), 120.59 (Ar-C), 124.16 (Ar-C), 125.97 (2 x C Ar), 127.94 (Ar-C), 128.37 (2 x C Ar), 128.96 (2 x C Ar), 134.38 (Ar-C), 135.03 (Ar-C), 149.31 (Ar-C), 150.91 (Ar-C), 151.08 (Ar-C), 153.32 (Ar-C), 159.06 (Ar-C), 164.87 (Ar-C). MS *m*/*z* (%) 471.45 (M+); Anal. Calcd. For: C_27_H_25_N_3_O_3_S (471.58): C, 68.77; H, 5.34; N, 8.91; S, 6.80; Found: C, 69.04; H, 5.60; N, 9.17; S, 6.93.

##### Synthesis of 2-(3-(3,4-Dimethoxyphenyl)-5-(4-methoxyphenyl)-4,5-dihydro-1H-pyrazol-1-yl)-4-(p-tolyl)thiazole (**6f**)

Brown powder, 45% yield; m.p. 150–152 °C; IR (KBr) νmax/cm^−1^ 2932 (C–H Str. of alkane), 1605, 1549; ^1^H NMR (DMSO-d_6_) *δ* 7.64 (d, *J* = 8.4 Hz, 2H, Ar), 7.37–7.28 (m, 4H, Ar), 7.2 (s, 1H, thiazole), 7.16 (d, *J* = 8.2 Hz, 3H, Ar), 7.03 (d, *J* = 8.4 Hz, 1H, Ar), 6.93 (d, *J* = 8.8 Hz, 2H, Ar), 5.6 (dd, *J* = 12, 6.4 Hz, 1H, H5 pyrazoline), 3.96 (dd, *J* = 18, 12 Hz, 1H, H_b_4 pyrazoline), 3.84 (s, 3H, 3-OCH_3_), 3.82 (s, 3H, 4-OCH_3_), 3.71 (s, 3H, 4-OCH_3_), 3.34 (dd, *J* = 18, 6.4 Hz, 1H, H_a_4 pyrazoline), 2.296 (s, 3H, CH_3_); ^13^C NMR (DMSO-d_6_) *δ* 21.25 (CH_3_), 43.65 (C4 pyrazoline), 55.51 (4-OCH_3_), 56.00 (4-OCH_3_), 56.08 (3-OCH_3_), 64.02 (C5 pyrazoline), 103.47 (Ar-C), 109.45 (Ar-C), 112.06 (Ar-C), 114.31 (2 x C Ar), 120.57 (Ar-C), 124.17 (Ar-C), 125.92 (2 x C Ar), 128.39 (2 x C Ar), 129.52 (2 x C Ar), 132.39 (Ar-C), 134.38 (Ar-C), 137.21 (Ar-C), 149.30 (Ar-C), 150.98 (Ar-C), 151.06 (Ar-C), 153.23 (Ar-C), 159.05 (Ar-C), 164.79 (Ar-C). MS (ESI^+^) *m*/*z* (%) 486.7 [M + H]^+^; Anal. Calcd. For: C_28_H_27_N_3_O_3_S (485.6): C, 69.26; H, 5.6; N, 8.65; S, 6.6; Found: C, 69.47; H, 5.78; N, 8.81; S, 6.72.

##### Synthesis of 2-(3-(3,4-Dimethoxyphenyl)-5-(4-methoxyphenyl)-4,5-dihydro-1H-pyrazol-1-yl)-4-(4-fluorophenyl)thiazole (**6g**)

Brown powder, 58% yield; m.p. 140–143 °C; IR (KBr) νmax/cm^−1^ 1602, 1550; ^1^H NMR (DMSO-d_6_) *δ* 7.79 (td, *J =* 5.6, 3.2 Hz, 2H, 4-FC_6_H_4_), 7.38 (d, *J =* 1.6 Hz, 1H, Ar), 7.33 (d, *J =* 8,8 Hz, 2H, Ar), 7.29 (dd, *J =* 8, 2 Hz, 1H, Ar), 7.25 (s, 1H, thiazole), 7.19 (t, *J =* 9.2 Hz, 2H, 4-FC_6_H_4_), 7.02 (d, *J =* 8.4 Hz, 1H, Ar), 6.91 (d, *J =* 8.4 Hz, 2H, Ar), 5.6 (dd, *J* = 11.6, 6.4 Hz, 1H, H5 pyrazoline), 3.96 (dd, *J* = 17.6, 11.6 Hz, 1H, H_b_4 pyrazoline), 3.84 (s, 3H, 3-OCH_3_), 3.81 (s, 3H, 4-OCH_3_), 3.71 (s, 3H, 4-OCH_3_), 3.34 (dd, *J* = 17.6, 6.4 Hz, 1H, H_a_4 pyrazoline); ^13^C NMR (DMSO-d_6_) *δ* 43.69 (C4 pyrazoline), 55.47 (4-OCH_3_), 55.97 (3-OCH_3_), 56.04 (4-OCH_3_), 64.04 (C5 pyrazoline), 104.11 (Ar-C), 109.46 (Ar-C), 112 (Ar-C), 114.33 (2 x C Ar), 115.67 (Ar-C), 115.89 (Ar-C), 120.59 (Ar-C), 124.15 (Ar-C), 127.88 (Ar-C), 127.96 (Ar-C), 128.36 (2 x C Ar), 131.66 (Ar-C), 131.69 (Ar-C), 134.32 (Ar-C), 149.32 (Ar-C), 149.9 (Ar-C), 151.11 (Ar-C), 153.36 (Ar-C), 159.07 (Ar-C), 160.83 (Ar-C), 163.26 (Ar-C), 164.97 (Ar-C). MS (APCI^+^) *m*/*z* (%) 490.63 [M + H]^+^; Anal. Calcd. For: C_27_H_24_N_3_O_3_SF (489.57): C, 66.24; H, 4.94; N, 8.58; S, 6.55; Found: C, 66.51; H, 5.11; N, 8.75; S, 6.68.

##### Synthesis of 4-(4-Chlorophenyl)-2-(3-(3,4-dimethoxyphenyl)-5-(4-methoxyphenyl)-4,5-dihydro-1H-pyrazol-1-yl)thiazole (**6h**)

White powder, 55% yield; m.p. 149–151 °C; IR (KBr) *ν*max/cm^−1^ 1608 (C=N), 1552 (C=C); ^1^H NMR (DMSO-d_6_) *δ* 7.76 (d, *J* = 8.5 Hz, 2H, Ar), 7.42 (d, *J* = 8.7 Hz, 2H, Ar) 7.37–7.28 (m, 5H, Ar), 7.05 (d, *J* = 8.5 Hz, 1H, Ar), 6.92 (d, *J* = 8.7 Hz, 2H, Ar), 5.62 (dd, *J* = 11.8, 6.4 Hz, 1H, H5 pyrazoline), 4.00 (dd, *J* = 18, 11.8 Hz, 1H, H_b_4 pyrazoline), 3.85 (s, 3H, 3-OCH_3_), 3.83 (s, 3H, 4-OCH_3_), 3.72 (s, 3H, 4-OCH_3_), 3.35 (dd, *J* = 18, 6.4 Hz, 1H, H_a_4 pyrazoline); ^13^C NMR (DMSO-d_6_) *δ* 43.70 (C4 pyrazoline), 55.52 (-OCH_3_), 56.01 (-OCH_3_), 56.09 (-OCH_3_), 63.98 (C5 pyrazoline), 105.21 (Ar-C), 109.49 (Ar-C), 112.07 (Ar-C), 114.35 (2 x C Ar), 120.63 (Ar-C), 124.09 (Ar-C), 127.64 (2 x C Ar), 128.38 (2 x C Ar), 128.99 (2 x C Ar), 132.34 (Ar-C), 133.87 (Ar-C), 134.24 (Ar-C), 149.30 (Ar-C), 149.66 (Ar-C), 151.11 (Ar-C), 153.53 (Ar-C), 159.07 (Ar-C), 164.95 (Ar-C). MS (ESI^-^) *m*/*z* (%) 505.2 [M-H]^-^, 508.5 [M + 2]^+^; Anal. Calcd. For: C_27_H_24_N_3_O_3_SCl (506): C, 64.09; H, 4.78; N, 8.3; S, 6.34; Found: C, 64.23; H, 4.93; N, 8.47; S, 6.50.

##### Synthesis of 2-(5-(4-Chlorophenyl)-3-(3,4-dimethoxyphenyl)-4,5-dihydro-1H-pyrazol-1-yl)-4-phenylthiazole (**6i**)

Brown powder, 66% yield; m.p. 208–210 °C; IR (KBr) νmax/cm^−1^ 1599 (C = N), 1544 (C = C); ^1^H NMR (DMSO-d_6_) *δ* 7.72 (d, *J* = 7.3 Hz, 2H, Ar), 7.44–7.46 (m, 4H, Ar), 7.38–7.24 (m, 6H, Ar), 7.06 (d, *J* = 7.3 Hz, 1H, Ar), 5.67 (dd, *J* = 11.9, 6.5 Hz, 1H, H5 pyrazoline), 4.04 (dd, *J* = 18, 11.9 Hz, 1H, H_b_4 pyrazoline), 3.85 (s, 3H, 3-OCH_3_), 3.83 (s, 3H, 4-OCH_3_), 3.37 (dd, *J* = 18, 6.5 Hz, 1H, H_a_4 pyrazoline); ^13^C NMR (DMSO-d_6_) *δ* 43.67 (C4 pyrazoline), 56.01 (4-OCH_3_), 56.09 (3-OCH_3_), 63.97 (C5 pyrazoline), 104.68 (Ar-C), 109.52 (Ar-C), 112.06 (Ar-C), 120.68 (Ar-C), 123.95 (Ar-C), 125.93 (Ar-C), 128 (3 x C Ar), 129.(4 x C Ar), 129.03 (Ar-C), 132.45 (Ar-C), 134.91 (Ar-C), 141.42 (Ar-C), 149.29 (Ar-C), 150.88 (Ar-C), 151.15 (Ar-C), 153.45 (Ar-C), 164.90 (Ar-C); MS (APCI^+^) *m*/*z* (%) 476.4 [M^+^], 478.4 [M + 2]^+^; Anal. Calcd. For: C_26_H_22_N_3_O_2_S CL (476): C, 65.61; H, 4.66; N, 8.83; S, 6.74; Found: C, 65.43; H, 4.85; N, 9.12; S, 6.78.

##### Synthesis of 2-(5-(4-Chlorophenyl)-3-(3,4-dimethoxyphenyl)-4,5-dihydro-1H-pyrazol-1-yl)-4-(p-tolyl)thiazole (**6j**)

Brown powder, 60% yield; m.p. 189–191 °C; IR (KBr) νmax/cm^−1^ 1601 (C=N), 1547 (C=C); ^1^H NMR (DMSO-d_6_) *δ* 7.60 (d, *J* = 8.1 Hz, 2H, Ar), 7.44–7.46 (m, 4H, Ar) 7.36 (d, *J* = 1.9 Hz, 1H, Ar), 7.31 (dd, *J* = 8.4, 1.9 Hz, 1H, Ar), 7.23 (s, 1H, thiazole), 7.16 (d, *J* = 8.1 Hz, 2H, Ar), 7.05 (d, *J* = 8.4 Hz, 1H, Ar), 5.67 (dd, *J* = 11.8, 6.4 Hz, 1H, H5 pyrazoline), 4.03 (dd, *J* = 18, 11.8 Hz, 1H, H_b_4 pyrazoline), 3.84 (s, 3H, 3-OCH_3_), 3.82 (s, 3H, Hs of 4-OCH_3_), 3.34 (dd, *J* = 18, 6.4 Hz, 1H, H_a_4 pyrazoline), 2.29 (s, 3H, CH_3_); ^13^C NMR (DMSO-d_6_) *δ* 21.26 (CH_3_), 43.65 (C4 pyrazoline), 56.02 (4-OCH_3_), 56.1 (3-OCH_3_), 63.97 (C5 pyrazoline), 103.75 (Ar-C), 109.54 (Ar-C), 112.08 (Ar-C), 120.65 (Ar-C), 123.99 (Ar-C), 125.88 (2 x C Ar), 128.98 (2 x C Ar), 129.05 (2 x C Ar), 129.54 (2 x C Ar), 132.29 (Ar-C), 132.42 (Ar-C), 137.25 (Ar-C), 141.46 (Ar-C), 149.30 (Ar-C), 150.93 (Ar-C), 151.14 (Ar-C), 153.37 (Ar-C), 164.80 (Ar-C). MS *m*/*z* (%) 491.4 [M + H]^+^; Anal. Calcd. For: C_27_H_24_N_3_O_2_S Cl (490): C,66.18; H, 4.94; N, 8.58; S, 6.54; Found: C, 66.46; H, 5.11; N, 8.79; S, 6.61.

##### Synthesis of 2-(5-(4-Chlorophenyl)-3-(3,4-dimethoxyphenyl)-4,5-dihydro-1H-pyrazol-1-yl)-4-(4-fluorophenyl)thiazole (**6k**)

Yellow powder, 68% yield; m.p. 203–205 °C; IR (KBr) νmax/cm^−1^ 3107(C–H Str. of alkene), 1638.46, 1601, 1543.18; ^1^H NMR (DMSO-d_6_) *δ* 7.74 (dt, *J =* 6*,* 2.8 Hz, 2H, 4-FC_6_H_4_), 7.44 (s, 4H, 4- ClC_6_H_4_), 7.36–7.32 (m, 2H, Ar), 7.30 (s, 1H, thiazole), 7.19 (t, *J* = 8.9 Hz, 2H, 4- FC_6_H_4_), 7.05 (d, *J* = 8.4 Hz, 1H, Ar), 5.67 (dd, *J* = 12, 6.4 Hz, 1H, H5 pyrazoline), 4.04 (dd, *J* = 18, 12 Hz, 1H, H_b_4 pyrazoline), 3.84 (s, 3H, 3-OCH_3_), 3.82 (s, 3H, 4-OCH_3_), 3.36 (dd, *J* = 18, 6.4 Hz, 1H, H_a_4 pyrazoline); ^13^C NMR (DMSO-d_6_) *δ* 43.70 (C4 pyrazoline), 56.03 (-OCH_3_), 56.11 (-OCH_3_), 63.94 (C5 pyrazoline), 104.41 (Ar-C), 109.59 (Ar-C), 112.09 (Ar-C), 115.72 (Ar-C), 115.94 (Ar-C), 120.69 (Ar-C), 123.94 (Ar-C), 127.85 (Ar-C), 127.93 (Ar-C), 129.02 (4 x C Ar), 131.54 (Ar-C), 131.57 (Ar-C), 132.46 (Ar-C), 141.40 (Ar-C), 149.31 (Ar-C), 149.84 (Ar-C), 151.19 (Ar-C), 153.55 (Ar-C), 160.83 (Ar-C), 163.26 (Ar-C), 164.99 (Ar-C). MS (APCI^-^) *m*/*z* (%) 494.2 [M^+^], 495.6 [M + 2]^+^; Anal. Calcd. For: C_26_H_21_N_3_O_2_SFCl (494): C, 63.22; H, 4.29; N, 8.51; S, 6.49; Found: C, 63.51; H, 4.45; N, 8.75; S, 6.42.

##### Synthesis of 4-(4-Chlorophenyl)-2-(5-(4-chlorophenyl)-3-(3,4-dimethoxyphenyl)-4,5-dihydro-1H-pyrazol-1-yl)thiazole (**6l**)

White powder, 74% yield; m.p. 195–197 °C; IR (KBr) νmax/cm^−1^ 2966 (C–H Str. of alkane), 1600 (C=N), 1545 (C=C); ^1^HNMR (DMSO-d_6_) *δ* 7.73 (d, *J* = 8.5 Hz, 2H, Ar), 7.36–7.43 (m, 8H, Ar), 7.31 (d, *J* = 11.9, 6.4 Hz, 1H, Ar), 7.04 (d, 1H, Ar), 5.67 (dd, *J* = 11.9, 6.4 Hz, 1H, H5 pyrazoline), 4.04 (dd, *J* = 18, 11.9 Hz, 1H, H_b_4 pyrazoline), 3.84 (s, 3H, 3-OCH_3_), 3.82 (s, 3H, 4-OCH_3_), 3.36 (dd, *J* = 18, 6.4 Hz, 1H, H_a_4 pyrazoline); ^13^C NMR (DMSO-d_6_) *δ* 43.67 (C4 pyrazoline), 56.01 (4-OCH_3_), 56.09 (3-OCH_3_), 63.90 (C5 pyrazoline), 105.46 (Ar-C), 109.54 (Ar-C), 112.05 (Ar-C), 120.71 (Ar-C), 123.89 (Ar-C), 127.61 (2 x C Ar), 129.02 (6 x C Ar), 132.41 (Ar-C), 132.49 (Ar-C),133.76 (Ar-C), 141.30 (Ar-C), 149.29 (Ar-C), 149.64 (Ar-C), 151.19 (Ar-C), 153.63 (Ar-C),164.99 (Ar-C). MS (ESI^-^) *m*/*z* (%) 512.2 (M^+^ + 2); Anal. Calcd. For: C_26_H_21_N_3_O_2_S Cl_2_ (510.43): C, 61.18; H, 4.15; N, 8.23; S, 6.28; Found: C, 61.40; H, 4.29; N, 8.51; S, 6.37.

##### Synthesis of 1-(2-(3-(3,4-Dimethoxyphenyl)-5-aryl-4,5-dihydro-1H-pyrazol-1-yl)-4-methylthiazol-5-yl)ethan-1-one (**9a**–**f**)

To a solution of 3-(3,4-dimethoxyphenyl)-5-aryl-4,5-dihydro-1*H*-pyrazole-1-carbothioamide derivatives **3a**, **c** (0.6 mmol) in absolute ethanol (10 mL), 3-chloropentane-2,4-dione (0.7 mmol) was added then refluxed at 80 °C for 4 h. Direct hot filtration proceeded and the obtained solid was washed with hot ethanol and recrystallized from EtOH/DMF to give the aimed compounds **9a**–**f**.

##### Synthesis of 1-(2-(3-(3,4-Dimethoxyphenyl)-5-phenyl-4,5-dihydro-1H-pyrazol-1-yl)-4-methylthiazol-5-yl)ethan-1-one (**9a**)

Greenish white powder, 63% yield; m.p 235–238 °C; IR (KBr) νmax/cm^−1^ 3003 (C–H Str. of alkene), 2928 (C–H Str. of alkane), 1613 (C=O), 1542(C = C); ^1^H NMR (DMSO-d_6_) *δ* 7.32–7.24 (m, 7H, Ar), 7.05 (d, *J* = 8.4 Hz,1H, Ar), 5.75 (dd, *J* = 12, 4.8 Hz, 1H, H5 pyrazoline), 4.04 (dd, *J* = 18, 12 Hz, 1H, H_b_4 pyrazoline), 3.838 (s, 3H, 3-OCH_3_), 3.82 (s, 3H, 4-OCH_3_), 3.36 (dd, *J* = 18, 6.4 Hz, 1H, H_a_4 pyrazoline), 2.39 (s, 3H, CH_3_), 2.37 (s, 3H, COCH_3_); ^13^C NMR (DMSO-d_6_) *δ* 19.11 (CH_3_), 30.02 (CH_3_), 44.15 (C4 pyrazoline), 56.04 (4-OCH_3_), 56.14 (3-OCH_3_), 63.32 (C5 pyrazoline), 109.61 (Ar-C), 112.04 (Ar-C), 121.23 (Ar-C), 123.41 (Ar-C), 123.97 (Ar-C), 126.20 (2 x C Ar), 128.06 (Ar-C), 129.28 (2 x C Ar), 141.79 (Ar-C), 149.31 (Ar-C), 151.6 (Ar-C), 156.23 (Ar-C), 157.78 (Ar-C), 165.11 (Ar-C), 189.48.(C= O); MS (ESI^-^) *m*/*z* (%) 420.2 [M-1]^-^; Anal. Calcd. For: C_23_H_23_N_3_O_3_S (421.52): C, 65.54; H, 5.5; N, 9.97; S, 7.61; Found: C, 65.78; H, 5.63; N, 10.15; S, 7.68.

##### Synthesis of 1-(2-(3-(3,4-Dimethoxyphenyl)-5-(4-methoxyphenyl)-4,5-dihydro-1H-pyrazol-1-yl)-4-methylthiazol-5-yl)ethan-1-one (**9b**)

Greenish yellow powder, 75% yield; m.p. 213–215 °C; IR (KBr) νmax/cm^−1^ 1613 (C=O), 1535 (C=C), 1510; ^1^H NMR (DMSO-d_6_) *δ* 7.368 (d, *J* = 2 Hz, 1H, Ar), 7.31 (dd, *J* = 8.4, 1.6 Hz, 1H, Ar), 7.17 (dd, *J* = 6.8, 2 Hz, 2H, Ar), 7.05 (dd, *J* = 8.4, 2 Hz, 1H, Ar), 6.8 (dd, *J* = 6.8, 2 Hz, 2H, Ar), 5.68 (dd, *J* = 11.6, 4.4 Hz, 1H, H5 pyrazoline), 3.99 (dd, *J* = 18, 11.6 Hz, 1H, H_b_4 pyrazoline), 3.839 (s, 3H, 3-OCH_3_), 3.82 (s, 3H, 4-OCH_3_), 3.72 (s, 3H, 4-OCH_3_), 3.33 (dd, *J* = 18, 4.4 Hz, 1H, H_a_4 pyrazoline), 2.39 (s, 3H, 4-CH_3_), 2.38 (s, 3H, COCH_3_); ^13^C NMR (DMSO-d_6_) *δ* 19.14 (CH_3_), 30.01 (CH_3_), 44.02 (C4 pyrazoline), 55.55 (OCH_3_), 56.03 (OCH_3_), 56.13 (OCH_3_), 62.90 (C5 pyrazoline), 109.56 (Ar-C), 112.02 (Ar-C), 114.57 (2 x C Ar), 121.20 (Ar-C), 123.50 (Ar-C), 123.86 (Ar-C), 127.62 (2 x C Ar), 133.72 (Ar-C), 149.32 (Ar-C), 151.58 (Ar-C), 156.20 (Ar-C), 157.78 (Ar-C), 159.12 (Ar-C), 165.08 (Ar-C), 189.37 (C=O); MS *m*/*z* (%) 451.39 [M^+^]; Anal. Calcd. For: C_24_H_25_N_3_O_4_S (451.54): C, 63.84; H, 5.58; N, 9.31; S, 7.1; Found: C, 64.09; H, 5.67; N, 9.48; S, 7.28.

##### Synthesis of 1-(2-(5-(4-Chlorophenyl)-3-(3,4-dimethoxyphenyl)-4,5-dihydro-1H-pyrazol-1-yl)-4-methylthiazol-5-yl)ethan-1-one (**9c**)

Greenish yellow powder, 65% yield; m.p. 215–218 °C; IR (KBr) νmax/cm^−1^ 3003 (C–H Str. of alkene), 2960 (C–H Str. of alkane), 1617 (C=O), 1536, 1509; ^1^H NMR (DMSO-d_6_) *δ* 7.45–7.40 (m, 2H, Ar), 7.36 (d, *J* = 2.0 Hz, 1H, Ar), 7.32 (dd, *J* = 8.3, 2.0 Hz, 1H, Ar), 7.30–7.25 (m, 2H, Ar), 7.05 (d, *J* = 8.4 Hz, 1H, Ar), 5.75 (dd, *J* = 12, 4.8 Hz, 1H, H5 pyrazoline), 4.04 (dd, *J =* 18, 12 Hz, 1H, H_b_4 pyrazoline), 3.835 (s, 3H, 3-OCH_3_), 3.82 (s, 3H, 4-OCH_3_), 3.3 (dd, *J* = 18, 4.8 Hz, 1H, H_a_4 pyrazoline), 2.39 (s, 3H, CH_3_), 2.38 (s, 3H, COCH_3_); ^13^C NMR (DMSO-d_6_) *δ* 19.14 (CH_3_), 29.98 (CH_3_), 43.93 (C4 pyrazoline), 56.00 (OCH_3_), 56.11 (OCH_3_), 62.74 (C5 pyrazoline), 109.61 (Ar-C), 112.00 (Ar-C), 121.26 (Ar-C), 123.31 (Ar-C), 124.12 (Ar-C), 128.31 (2 x C Ar), 129.23 (2 x C Ar), 132.59 (Ar-C), 140.72 (Ar-C), 149.29 (Ar-C), 151.62 (Ar-C), 156.14 (Ar-C), 157.68 (Ar-C), 165.04 (Ar-C), 189.46 (C=O); MS (APCI^+^) *m*/*z* (%) 456.5 [M^+^], 458.7 [M + 2]^+^; Anal. Calcd. For: C_23_H_22_N_3_O_3_SCL (456): C, 60.59; H, 4.86; N, 9.22; S, 7.03; Found: C, 60.81; H, 5.08; N, 9.41; S, 7.14.

##### Synthesis of Ethyl 2-(3-(3,4-Dimethoxyphenyl)-5-phenyl-4,5-dihydro-1H-pyrazol-1-yl)-4-methylthiazole-5-carboxylate (**9d**)

Greenish yellow powder, 65% yield; m.p. 215–218 °C; IR (KBr) νmax/cm^−1^ 1695 (C=O), 1637, 1507; ^1^H NMR (DMSO-d_6_) *δ* 7.38–7.24 (m, 7H, Ar), 7.04 (d, *J* = 8.4 Hz, 1H, Ar), 5.72 (dd, *J* = 11.6, 4.8 Hz, 1H, H5 pyrazoline), 4.19 (q, *J* = 7.0 Hz, 2H, CH_2_), 4.05 (dd, *J =* 18, 11.6 Hz, 1H, H_b_4 pyrazoline), 3.84 (s, 3H, 3-OCH_3_), 3.82 (s, 3H, 4- OCH_3_), 3.3 (dd, *J* = 18, 4.8 Hz, 1H, H_a_4 of pyrazoline), 2.35 (s, 3H, CH_3_), 1.25 (t, *J =* 7.1 Hz, 3H, -CH_2_-CH_3_); ^13^C NMR (DMSO-d_6_) *δ* 14.79 (-CH_2_-CH_3_), 17.94 (CH_3_), 44.2 (C4 pyrazoline), 56.05 (OCH3), 56.13 (OCH3), 60.60 (CH_2_CH_3_), 63.40 (C5 pyrazoline), 109.61 (Ar-C), 110.61 (Ar-C), 112.03 (Ar-C), 121.20 (Ar-C), 123.43 (Ar-C), 126.21 (2 x C Ar), 128.04 (Ar-C), 129.27 (2 x C Ar), 141.81 (Ar-C), 149.31 (Ar-C), 151.56 (Ar-C), 155.94 (Ar-C), 159.73 (Ar-C), 162.34 (C=O), 165.21(Ar-C); MS (ESI^−^) *m*/*z* (%) 450.4 [M-H]^−^; Anal. Calcd. For: C_24_H_25_N_3_O_4_S (451.54): C, 63.84; H, 5.58; N, 9.3; S, 7.1; Found: C, 64.07; H, 5.69; N, 9.53; S, 7.23.

##### Synthesis of Ethyl 2-(3-(3,4-Dimethoxyphenyl)-5-(4-methoxyphenyl)-4,5-dihydro-1H-pyrazol-1-yl)-4-methylthiazole-5-carboxylate (**9e**)

Yellow powder, 77.5% yield; m.p. 169–171 °C; IR (KBr) νmax/cm^−1^ 1698 (C=O), 1601 (C=N), 1545; ^1^H NMR (DMSO-d_6_) *δ* 7.37 (d, *J =* 2 Hz, 1H, Ar), 7.32 (dd, *J* = 8, 2 Hz, 1H, Ar), 7.18 (dd, *J* = 6.8, 2 Hz, 2H, Ar), 7.04 (d, *J* = 8.5 Hz, 1H, Ar), 6.90 (d, *J* = 6.8, 2 Hz, 2H, Ar) 5.66 (dd, *J* = 11.6, 4.8 Hz, 1H, H5 pyrazoline), 4.19 (q, *J* = 7.0 Hz, 2H, CH_2_), 4.01 (dd, *J =* 18, 11.6 Hz, 1H, H_b_4 pyrazoline), 3.84 (s, 3H, 3-OCH_3_), 3.82 (s, 3H, 4-OCH_3_), 3.72 (s, 3H, 4- OCH_3_), 3.32 (dd, *J* = 18, 4.8 Hz, 1H, H_a_4 pyrazoline), 2.36 (s, 3H, 4-CH_3_), 1.25 (t, *J =* 7.1 Hz, 3H, CH_2_-CH_3_); ^13^CNMR (DMSO-d_6_) *δ* 14.78 (CH_2_-CH_3_), 17.92 (CH_3_), 44.10 (C4 pyrazoline), 55.55 (OCH3), 56.05(OCH_3_), 56.12 (OCH_3_), 60.59 (CH_2_CH_3_), 63.01 (C5 pyrazoline), 109.58 (Ar-C), 110.39 (Ar-C), 112.02 (Ar-C), 114.57 (2 x C Ar), 121.20 (Ar-C), 123.50 (Ar-C), 127.64 (2 x C Ar), 133.67 (Ar-C), 149.31 (Ar-C), 151.55 (Ar-C), 156.02 (Ar-C), 159.11 (Ar-C), 159.61 (Ar-C), 162.33 (C=O), 165.11 (Ar-C); MS (ESI^+^) *m*/*z* (%) 482.6 [M + 1]^+^; Anal. Calcd. For: C_25_H_27_N_3_O_5_S (481.57): C, 62.35; H, 5.65; N, 8.73; S, 6.66; Found: C, 62.51; H, 5.83; N, 8.97; S, 6.81.

##### Synthesis of Ethyl 2-(5-(4-Chlorophenyl)-3-(3,4-dimethoxyphenyl)-4,5-dihydro-1H-pyrazol-1-yl)-4-methylthiazole-5-carboxylate (**9f**)

Yellow powder, 61% yield; m.p. 197–200 °C; IR (KBr) *ν*max/cm^−1^ 2992 (C–H Str. of alkane), 1708 (C=O), 1602, 1571; ^1^H NMR (DMSO-d_6_) *δ* 7.42 (d, *J* = 8.4 Hz, 2H, Ar), 7.35 (d, *J =* 1.6 Hz, 1H, Ar), 7.27–7.32 (m, 3H, Ar), 7.04 (d, *J* = 8.4 Hz, 1H, Ar), 5.72 (dd, *J* = 11.6, 4.8 Hz, 1H, H5 pyrazoline), 4.19 (q, *J* = 7.0 Hz, 2H, CH_2_), 4.04 (dd, *J =* 18, 11.6 Hz, 1H, H_b_4 pyrazoline), 3.832 (s, 3H, 3-OCH_3_), 3.817 (s, 3H, 4-OCH_3_), 3.34 (dd, *J* = 18, 4.8 Hz, 1H, H_a_4 pyrazoline), 2.35 (s, 3H, 4-CH_3_), 1.25 (t, *J =* 7.1 Hz, 3H, -CH_2_CH_3_); ^13^C NMR (DMSO-d_6_) *δ* 14.78 (CH_2_CH_3_), 17.93 (CH_3_), 43.99 (C4 pyrazoline), 56.05 (OCH_3_), 56.13 (OCH_3_), 60.63 (CH_2_CH_3_), 62.82 (C5 pyrazoline), 109.62 (Ar-C), 110.69 (Ar-C), 112.03 (Ar-C), 121.24 (Ar-C), 123.34 (Ar-C), 128.31 (2 x C Ar), 129.24 (2 x C Ar), 132.50 (Ar-C), 140.74 (Ar-C), 149.30 (Ar-C), 151.60 (Ar-C), 155.95 (Ar-C), 159.67 (Ar-C), 162.31 (C= O), 165.15 (Ar-C). MS *m*/*z* (%) 486.26 [M^+^], 487.91 [M + 1]; Anal. Calcd. For: C_24_H_24_N_3_O_4_SCl (486): C, 59.32; H, 4.98; N, 8.65; S, 6.6; Found: C, 59.58; H, 5.06; N, 8.89; S, 6.72.

### 3.2. Biological Evaluations

#### 3.2.1. Cytotoxicity

Both breast cancer (MCF-7) and normal breast (MCF-10A) cells were purchased from National Research Institute, Egypt, and maintained in complete media of RPMI-1640 medium L-Glutamine (Lonza Verviers SPRL, Belgium, cat#12-604F). Cells were incubated following standard tissue culture work. Cell viability was measured 48 h later, utilizing MTT colorimetric assay (Promega, Madison, WI, USA) [63]. Absorbance was consequently assessed (at 570 nm) using an ELISA microplate reader (BIO-RAD, model iMark, Tokyo, Japan). Using GraphPad Prism 7, the IC_50_ values were determined, and the viability at each concentration was determined in comparison to the control [68,69].

#### 3.2.2. EGFR/HER2 Kinase Inhibitory Assay

The EGFR Kinase Assay Kit (BPS Bioscience kit, Cat#40321) and the HER2 Kinase Assay Kit (BPS Bioscience kit, Cat#40721) were used to evaluate the potential inhibitory activities against EGFR and HER2, respectively. The inhibitory potency of compounds **3c**, **6b**, **6e**, **6k**, and **9f** against EGFR and HER2 kinases was determined using kinase inhibitory assays. Compounds’ autophosphorylation percentage inhibition was computed according to the following equation: $100-\left[(\frac{A\;control}{A\;treated}-Control\right)]$, then IC_50_ values were calculated using GraphPad prism software [70].

#### 3.2.3. Investigation of Apoptosis

##### Annexin V/PI Staining and Cell Cycle Analysis

MCF-7 breast cancer cells were seeded into 6-well culture plates (3–5 × 10^5^ cells/well) and incubated for 24 h. Subsequently, compounds 6k and 6e (at their IC_50_ values) were used to treat the seeded cells for 48 h. After that, the collection of the media supernatants and the cells was performed before undergoing rinsing with ice-cold PBS. The cells were suspended in 100 µL of annexin-binding buffer solution “25 mM CaCl_2_, 1.4 M NaCl, and 0.1 M Hepes/NaOH, pH 7.4” and incubated with “Annexin V-FITC solution (1:100) and propidium iodide (PI)” at a concentration equal 10 µg/mL in the dark for 30 min. Stained cells were then obtained by a Cytoflex FACS machine, and data analysis was performed using cytExpert software [71,72,73].

##### Real-Time Polymerase Chain Reaction for the Selected Genes

Furthermore, to explore the apoptotic pathway, gene expression of proapoptotic genes (P53, Bax, Caspapses-3,8,9) and antiapoptotic gene (Bcl-2) was evaluated using routine RT-PCR in the untreated and treated MCF-7 cells. Ct values were calculated to determine the relative genes expression in all samples by normalization to β-actin (housekeeping gene) [71,74].

#### 3.2.4. In Vivo Study

##### Ethics Statement

The experimental protocol was approved by the Research Ethics Committee at Suez Canal University (Approval number REC-10-2021; October 2021), Chemistry Department, Faculty of Science, Suez Canal University.

##### Animal, Tumor Inoculation Experiment Design

In vivo experiments for lethal dose (LD_50_), experimental design, tumor mass, blood parameters, histopathological examinations were accomplished as previously described [71]. After inoculating the tumor cells, masses of the solid tumor started to appear ten days later. Seven administrations of **6e** and Lapatinib were completed, each at a dose of 10 mg/Kg BW/IP. Solid tumor masses’ weight and volume were measured at the end of the experiment. Animals from each group were sacrificed, and their blood was drawn to measure hemoglobin (Hb), red blood cell (RBC), white blood cell (WBC), and liver enzymes (ALT, AST, albumin, and total protein) levels.

### 3.3. Computational Studies

#### 3.3.1. Molecular Docking

Two PDB codes for the molecular targets in the proposed antiproliferative mechanism were selected. Both 1XKK and 3RCD were chosen as crystal structures for EGFR and HER2 receptors, respectively. Docking program used was AutoDock Vina [75,76], following multiple ligands’ docking protocol regarding water molecule deletion, as well as both ligand and receptor preparation [77]. Grid box size was prepared based on the active residues of the kinase domains of the receptors. After docking simulation, poses with the highest negative binding free energy (kcal/mol) were selected as the best poses for corresponding ligand binding. Visualization of the 3D receptor binding site, disposition of the original (co-crystallized) ligand, and the main ligand receptor interaction in terms of hydrogen bonding with the key amino acid residues was performed using Chimera software [78].

#### 3.3.2. In Silico Physicochemical Descriptors, Pharmacokinetic Properties, and Bioactivity Prediction

In silico estimation of physicochemical properties, pharmacokinetics and drug-likeness was performed using SwissADME web tool [65] and PreADMET server [79]. An osiris server [80] was used for toxicity prediction by measuring irritant, mutagenic, tumorigenic, and reproductive effects.

## 4. Conclusions

Herein, a novel series of pyrazoline derivatives **3a**–**c**, and thiazolyl-pyrazolines **6a**–**l** and **9a**–**f** were designed and synthesized as dual EGFR/HER2 inhibitors. All compounds were assessed for their anti-antiproliferative activities towards MCF-7 and MCF-10 (cancer and normal breast cell line). The results revealed that compounds 3c, 6b, 6e, 6f, 6k, and 9f displayed potent and selective anticancer activity against MCF-7 cell lines compared to lapatinib and were safe for the normal breast cell. Moreover, the highly potent compounds were investigated for their inhibitory properties against EGFR and HER2 compared to lapatinib. Interestingly, compound 6e showed potent EGFR and HER2 inhibitory effects (IC_50_ = 0.009 and 0.013 µM, respectively), superior to the reference drug lapatinib (IC_50_ = 0.006 and 0.017 µM, respectively). Moreover, compounds 6e and 6k also significantly induced apoptosis in the targeted breast cell line with 27.99% and 22.44%, respectively, compared to 0.68% for the control. Furthermore, the in vivo study for compound 6e showed a tumor inhibition ratio by 52.46% compared to 50.53% in lapatinib treatment. Molecular docking results revealed that compounds 6e and 6k had good binding affinity to the ATP binding site of EGFR with H bonds formed with the crucial amino acid residue Met793. Besides, compound 6e also anchored well in the HER2 binding site and participating in the crucial interaction with Thr862. According to the in vitro, in vivo, and in silico studies, compound 6e stands out as a potential drug candidate for the development of future anticancer agents, having a dual inhibitory activity against EGFR and HER2 receptors.

## Figures and Tables

**Table 1:** Cytotoxic IC_50_ values of the tested compounds against MCF-7 and MCF-10A lines using the MTT assay.

Compounds	IC_50_ ± SD * (µM)		Compounds	IC_50_ ± SD * (µM)	
	MCF-7	MCF-10A		MCF-7	MCF-10A
**3a**	NA	36.5 ± 1.15	**6i**	45.36 ± 1.87	15.82 ± 1.08
**3b**	23.6 ± 0.72	34.8 ± 1.35	**6j**	NA	≥50
**3c**	11.19 ± 0.54	≥50	**6k**	8.02 ± 0.98	≥50
**6a**	NA	≥50	**6l**	17.58 ± 0.97	35.7 ± 1.85
**6b**	13.36 ± 0.98	≥50	**9a**	34.25 ± 1.68	18.9 ± 1.09
**6c**	18.69 ± 0.84	≥50	**9b**	31.69 ± 1.96	26.34 ± 1.07
**6d**	NA	45.2 ± 1.24	**9c**	NA	≥50
**6e**	7.21 ± 0.48	≥50	**9d**	NA	24.3 ± 1.39
**6f**	11.05 ± 0.85	35.4 ± 1.84	**9e**	18.95 ± 1.03	43.6 ± 1.85
**6g**	15.36 ± 0.79	24.5 ± 1.01	**9f**	8.35 ± 0.29	≥50
**6h**	35.25 ± 1.27	26.5 ± 1.59	**Lapatinib**	7.45	≥50

* Values are expressed as mean ± SD of three independent triplets (n = 3). **NA**: Non-Active.

**Table 2:** IC_50_ values of EGFR and HER2 kinase activities of the tested compounds.

Compound	IC_50_ [µM] *	
	EGFR Kinase	HER2 Kinase
**3c**	0.067± 0.009	0.07 ± 0.018
**6f**	0.23 ± 0.024	0.12 ± 0.025
**6e**	0.009 ± 0.001	0.013 ± 0.001
**6k**	0.014 ± 0.001	0.027 ± 0.002
**9f**	0.051 ± 0.003	0.14 ± 0.006
**Lapatinib**	0.006 ± 0.001	0.017 ± 0.002

* Values are expressed as the average of three independent replicates (mean ± SD). IC_50_ values were calculated using a sigmoidal non-linear regression curve fit of percentage inhibition against five concentrations of each compound.

**Table 3:** Fold change of apoptosis-related genes in untreated and treated MCF-7 cells.

Sample	Fold Change = 2^−ΔΔCT^					
	Proapoptotic Gene					Anti-Apoptotic Gene
	P53	Bax	Casp-3	Casp-8	Casp-9	Bcl-2
**6k**- treated	3.91 ± 0.50	5.24 ± 0.4	5.79 ± 0.4	3.91 ± 0.42	6.07 ± 0.23	0.41 ± 0.09
**6e**- treated	4.85 ± 0.4	6.39 ± 0.8	7.12 ± 0.53	1.71 ± 0.18	8.19 ± 0.32	0.22 ± 0.06
Untreated	1					

Values are expressed as mean ± SD of three independent replicates. Data were normalized using β-actin as housekeeping gene.

## Data Availability

Data is contained within the article and supplementary material.

## Supplementary Materials

The following supporting information can be downloaded at: https://www.mdpi.com/article/10.3390/ph15101245/s1, Figure S1: Redocking validation for EGFR; RMSD = 0.6 and S-score = −11.83, Figure S2: Redocking validation for HER2; RMSD = 0.6 and S-score = −10.11, Figure S3: bioavalibility radar for Lapatinib, Figure S4: (**A**) bioavailibility radar for **6e**; (**B**) bioavailibility radar for **6k**, Figure S5: Boiled egg model as indication of bioavalibility, Figure S6: ^1^H NMR of compound **3a**, Figure S7: ^13^C NMR of compound **3a**, Figure S8: Mass of compound **3a**, Figure S9: IR of compound **3a**, Figure S10: ^1^H NMR of compound **3b**, Figure S6: ^1^H NMR (D_2_O) of compound **3b**, Figure S7: ^13^C NMR of compound **3b**, Figure S8: Mass of compound **3b**, Figure S9: IR of compound **3b**, Figure S10: ^1^H NMR of compound **3c**, Figure S11: ^13^C NMR of compound **3C**, Figure S12: Mass of compound **3C**, Figure S13: IR of compound **3C**, Figure S14: ^1^H NMR of compound **6a**, Figure S15: ^13^C NMR of compound **6a**, Figure S16: Mass of compound **6a**, Figure S17: IR of compound **6a**, Figure S18: ^1^H NMR of compound **6b**, Figure S19: ^13^C NMR of compound **6b**, Figure S20: Mass of compound **6b**, Figure S21: IR of compound **6b**, Figure S22: ^1^H NMR of compound **6C**, Figure S23: ^13^C NMR of compound **6C**, Figure S24: Mass of compound **6C**, Figure S25: ^1^H NMR of compound **6d**, Figure S26: ^13^C NMR of compound **6d**, Figure S27: Mass of compound **6d**, Figure S28: IR of compound **6d**, Figure S29: ^1^H NMR of compound **6e**, Figure S30: ^13^C NMR of compound **6e**, Figure S31: Mass of compound **6e**, Figure S32: IR of compound **6e**, Figure S33: ^1^H NMR of compound **6f**, Figure S34: ^13^C NMR of compound **6f**, Figure S35: Mass of compound **6f**, Figure S36: ^1^H NMR of compound **6g**, Figure S37: ^13^CNMR of compound **6g**, Figure S38: Mass of compound **6g**, Figure S39: ^1^H NMR of compound **6h**, Figure S40: ^13^C NMR of compound **6h**, Figure S41: Mass of compound **6h**, Figure S42: IR of compound **6h**, Figure S43: ^1^H NMR of compound **6i**, Figure S44: ^13^C NMR of compound **6i**, Figure S45: Mass of compound **6i**, Figure S46: IR of compound **6i**, Figure S47: ^1^H NMR of compound **6j**, Figure S48: ^13^C NMR of compound **6j**, Figure S49: Mass of compound **6j**, Figure S50: ^1^H NMR of compound **6k**, Figure S51: ^13^C NMR of compound **6k**, Figure S52: ^1^H−^1^H Cosy of compound **6k**, Figure S53: Mass of compound **6K**, Figure S54: ^1^H NMR of compound **6l**, Figure S55: ^13^C NMR of compound **6l**, Figure S56: Mass of compound **6l**, Figure S57: IR of compound **6l**, Figure S58: ^1^H NMR of compound **9a**, Figure S59: ^13^C NMR of compound **9a**, Figure S60: Mass of compound **9a**, Figure S61: ^1^H NMR of compound **9b**, Figure S62: ^13^C NMR of compound **9b**, Figure S63: Mass of compound **9b**, Figure S64: ^1^H NMR of compound **9c**, Figure S65: ^13^C NMR of compound **9c**, Figure S66: Mass of compound **9c**, Figure S67: IR of compound **9c**, Figure S68: ^1^H NMR of compound **9d**, Figure S69: ^13^C NHR of compound **9d**, Figure S70: Mass of compound **9d**, Figure S71: ^1^H NMR of compound **9e**, Figure S72: ^13^C NMR of compound **9e**, Figure S73: Mass of compound **9e**, Figure S74: IR of compound **9e**, Figure S75: ^1^H NMR of compound **9f**, Figure S76: ^13^C NMR of compound **9f**, Figure S77: Mass of compound **9f**, Figure S78: IR of compound **9f**, Figure S79: Elemental analysis (CHN) for **3a**–**c**, **6a**–**l**, **9a**–**f**.
