# Supplementary material for: Rational Design, Synthesis and Biological Evaluation of Novel Pyrazoline-Based Antiproliferative Agents in MCF-7 Cancer Cells
Source: Pharmaceuticals (Basel). 2022 Oct 10;15(10):1245. doi: 10.3390/ph15101245 (PMC9607164; doi:10.3390/ph15101245)
Supplement: Supplementary file 1 [file pharmaceuticals-15-01245-s001.zip › pharmaceuticals-1954618-supplementary.pdf]

Rational Design, Synthesis and Biological Evaluation of Novel Pyrazoline-Based Antiproliferative Agents in MCF-7 Cancer Cells

Ismail Salama [1,*], Safaa M. Kishk [1,*], Mariam M. Fakhry [2], Kazem Mahmoud [2], Mohamed S. Nafie [3], Ahmad O. Noor [4] and Rawan H. Hareeri [5]

[1] Department of Pharmaceutical Medicinal Chemistry, Faculty of Pharmacy, Suez Canal University,
Ismailia 41522, Egypt

[2] Department of Pharmaceutical Chemistry, Faculty of Pharmacy, Egyptian Russian University,
Badr 11829, Egypt

[3] Department of Chemistry, Faculty of Science, Suez Canal University, Ismailia 41522, Egypt
[4] Department of Pharmacy Practice, Faculty of Pharmacy, King Abdulaziz University, Jeddah 21589, Saudi Arabia
[5] Department of Pharmacology and Toxicology, Faculty of Pharmacy, King Abdulaziz University,
Jeddah 21589, Saudi Arabia

* Correspondence: ismail_mohamed@pharm.suez.edu.eg (I.S.); safaa_keshk@pharm.suez.edu.eg (S.M.K.)

Supplementary content

Figure S1: Redocking validation for EGFR; RMSD = 0.6 and S-score = -11. 83 1
Figure S2: Redocking validation for HER2; RMSD= 0.6 and S-score = -10.11 1
Figure S3: bioavalibility radar for Lapatinib 2
Figure S4: A) bioavailibility radar for 6e; B) bioavailibility radar for 6k 2
Figure S5: Boiled egg model as indication of bioavalibility........ 3
Figure S6: ^{1}H NMR of compound 3a........ 4
Figure S7: ^{13}C NMR of compound 3a........ 5
Figure S8: Mass of compound 3a 6
Figure S9: IR of compound 3a........ 6
Figure S10: ^{1}H NMR of compound 3b 7
Figure S11: ^{1}H NMR (D_2O) of compound 3b........ 8
Figure S12: ^{13}C NMR of compound 3b 9
Figure S13: Mass of compound 3b 10
Figure S14: IR of compound 3b........ 10
Figure S15: ^{1}H NMR of compound 3c........ 11
Figure S16: ^{13}C NMR of compound 3C........ 12
Figure S17: Mass of compound 3C........ 13
Figure S18: IR of compound 3C 13
Figure S19: ^{1}H NMR of compound 6a........ 14
Figure S20: ^{13}C NMR of compound 6a........ 15
Figure S21: Mass of compound 6a 16
Figure S22: IR of compound 6a........ 16
Figure S23: ^{1}H NMR of compound 6b 17
Figure S24: ^{13}C NMR of compound 6b 18
Figure S25: Mass of compound 6b 19
Figure S26: IR of compound 6b........ 19
Figure S27: ^{1}H NMR of compound 6C........ 20
Figure S28: ^{13}C NMR of compound 6C........ 21
Figure S29: Mass of compound 6C........ 22
Figure S30: ^{1}H NMR of compound 6d 23
Figure S31: ^{13}C NMR of compound 6d 24
Figure S32: Mass of compound 6d 25
Figure S33: IR of compound 6d........ 25
Figure S34: ^{1}H NMR of compound 6e........ 26
Figure S35: ^{13}C NMR of compound 6e........ 27
Figure S36: Mass of compound 6e 28
Figure S37: IR of compound 6e........ 28
Figure S38: ^{1}H NMR of compound 6f........ 29
Figure S39: ^{13}C NMR of compound 6f........ 30
Figure S40: Mass of compound 6f........ 31

Figure S41: ^{1}H NMR of compound 6g 32
Figure S42: ^{13}CNMR of compound 6g 33
Figure S43: Mass of compound 6g 34
Figure S44: ^{1}H NMR of compound 6h 35
Figure S45: ^{13}C NMR of compound 6h 36
Figure S46: Mass of compound 6h 37
Figure S47: IR of compound 6h 37
Figure S48: ^{1}H NMR of compound 6i 38
Figure S49: ^{13}C NMR of compound 6i 39
Figure S50: Mass of compound 6i 40
Figure S51: IR of compound 6i 40
Figure S52: ^{1}H NMR of compound 6j 41
Figure S53: ^{13}C NMR of compound 6j 42
Figure S54: Mass of compound 6j 43
Figure S55: ^{1}H NMR of compound 6k 44
Figure S56: ^{13}C NMR of compound 6k 45
Figure S57: ^{1}H-^{1}H Cosy of compound 6k 46
Figure S58: Mass of compound 6K 47
Figure S59: ^{1}H NMR of compound 6l 48
Figure S60: ^{13}C NMR of compound 6l 49
Figure S61: Mass of compound 6l 50
Figure S62: IR of compound 6l 50
Figure S63: ^{1}H NMR of compound 9a 51
Figure S64: ^{13}C NMR of compound 9a 52
Figure S65: Mass of compound 9a 53
Figure S66: ^{1}H NMR of compound 9b 54
Figure S67: ^{13}C NMR of compound 9b 55
Figure S68: Mass of compound 9b 56
Figure S69: ^{1}H NMR of compound 9c 57
Figure S70: ^{13}C NMR of compound 9c 58
Figure S71: Mass of compound 9c 59
Figure S72: IR of compound 9c 59
Figure S73: ^{1}H NMR of compound 9d 60
Figure S74: ^{13}C NHR of compound 9d 61
Figure S75: Mass of compound 9d 62
Figure S76: ^{1}H NMR of compound 9e 63
Figure S77: ^{13}C NMR of compound 9e 64
Figure S78: Mass of compound 9e 65
Figure S79: IR of compound 9e 65
Figure S80: ^{1}H NMR of compound 9f 66
Figure S81: ^{13}C NMR of compound 9f 67
Figure S82: Mass of compound 9f 68
Figure S83: IR of compound 9f 68
Figure S84: Elemental analysis (CHN) for 3a-c, 6a-l, 9a-f 70

Table S1: Predicted properties and Lipinski's parameters for target compounds and lapatinib as a reference........ 71
Table S2: Predicted *in silico* ADME properties for target compounds........ 71
Table S3: Toxicity prediction using Osiris server........ 72

Docking validation

1. **Validation for 1xkk**

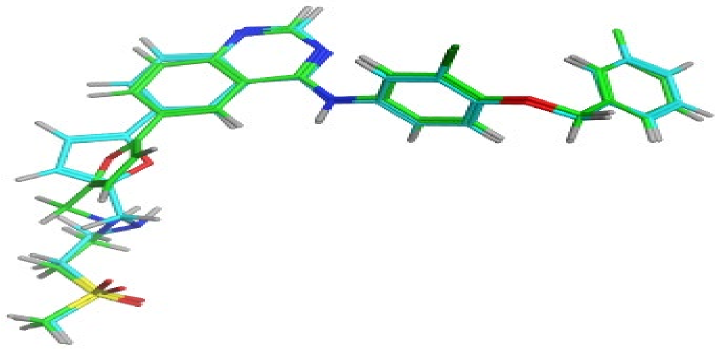

Figure S1: Redocking validation for EGFR; RMSD = 0.6 and S-score = -11. 83

2. **Validation for 3rcd**

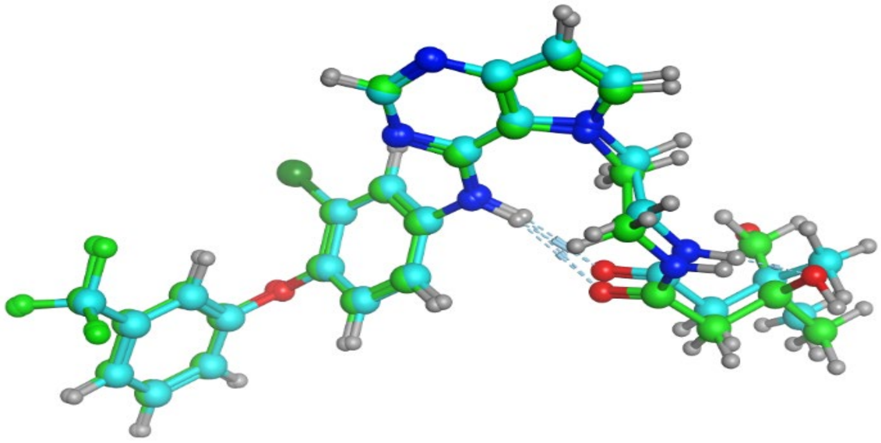

Figure S2: Redocking validation for HER2; RMSD= 0.6 and S-score = -10.11

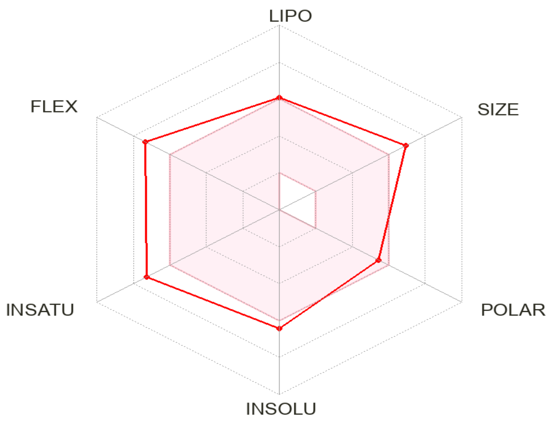

Figure S3: bioavalibility radar for Lapatinib

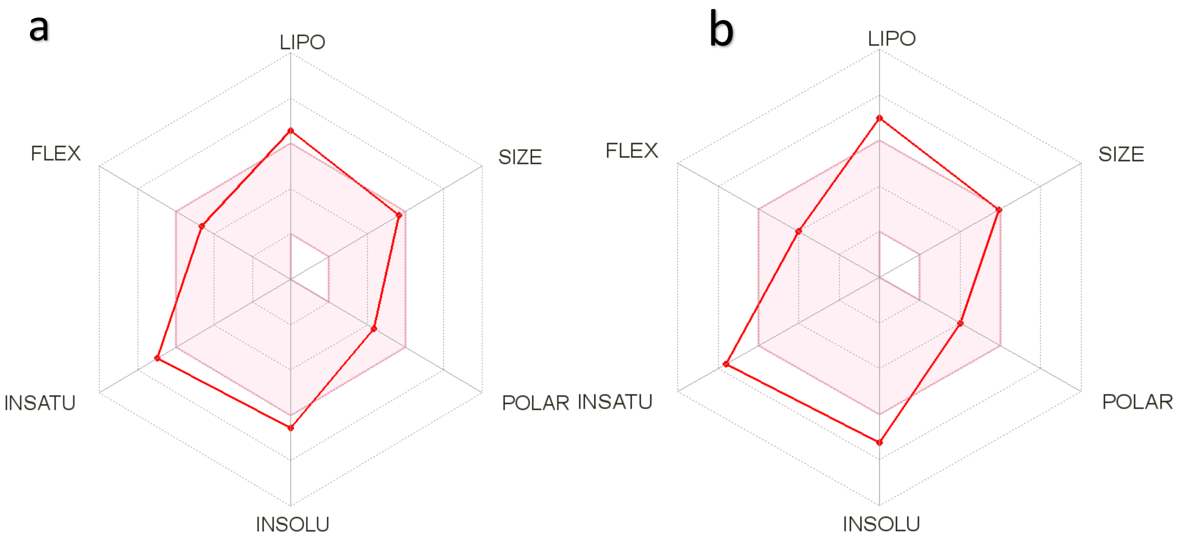

Figure S4: A) bioavailibility radar for 6e; B) bioavailibility radar for 6k

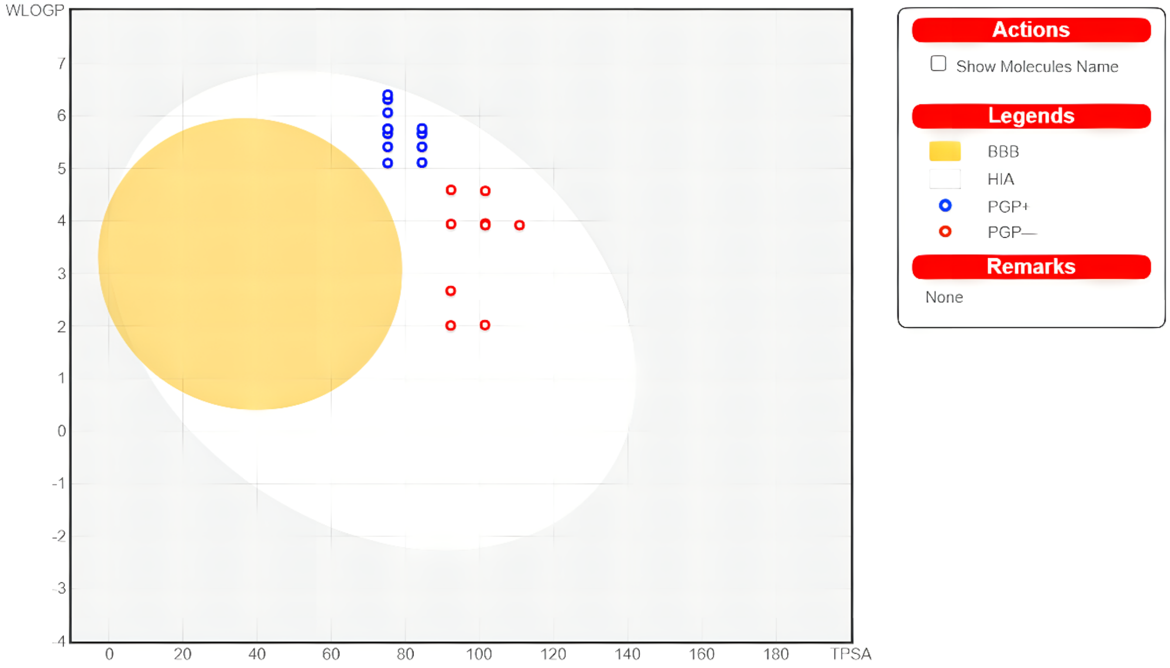

Figure S5: Boiled egg model as indication of bioavalibility

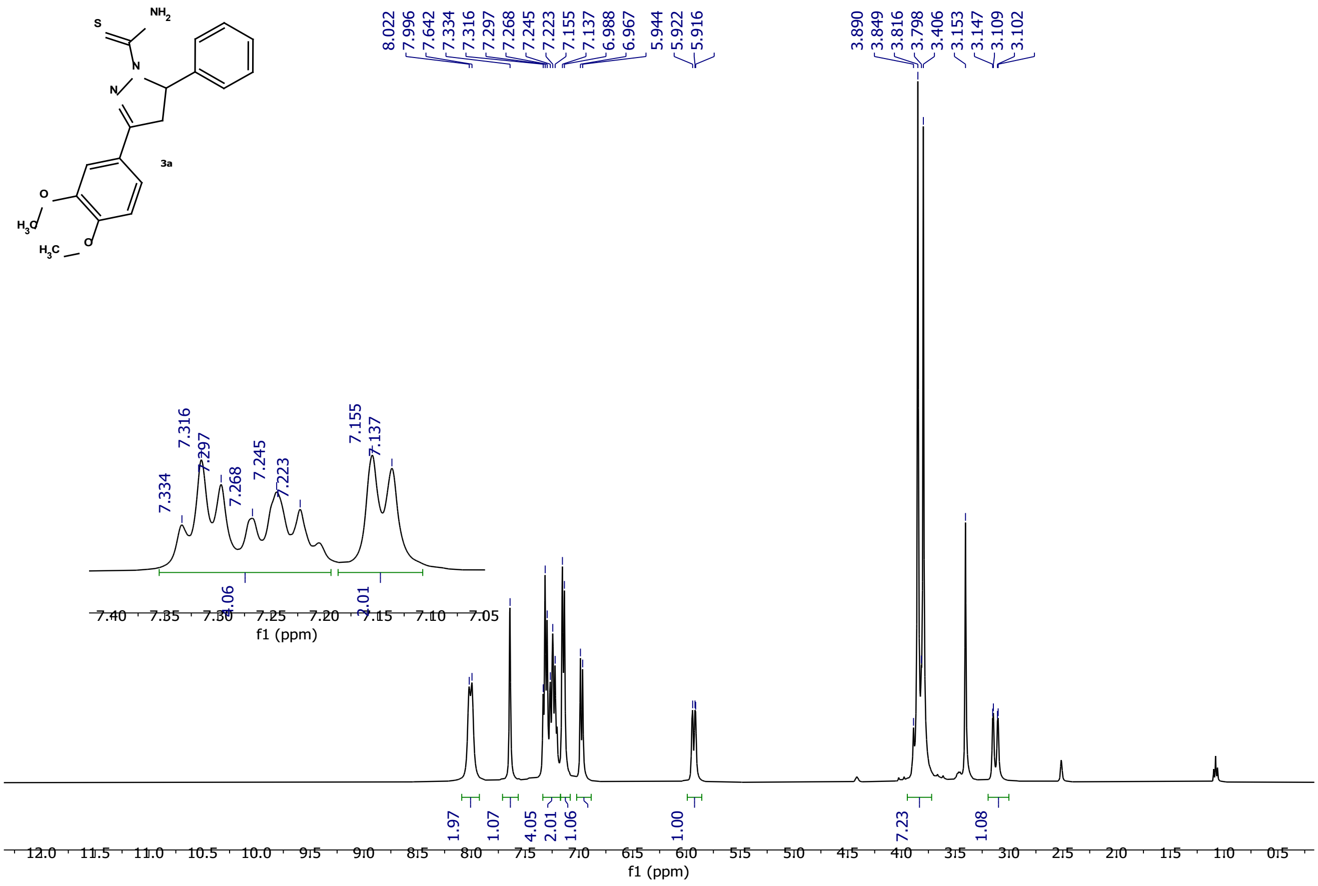

Figure S6: ^{1}H NMR of compound 3a

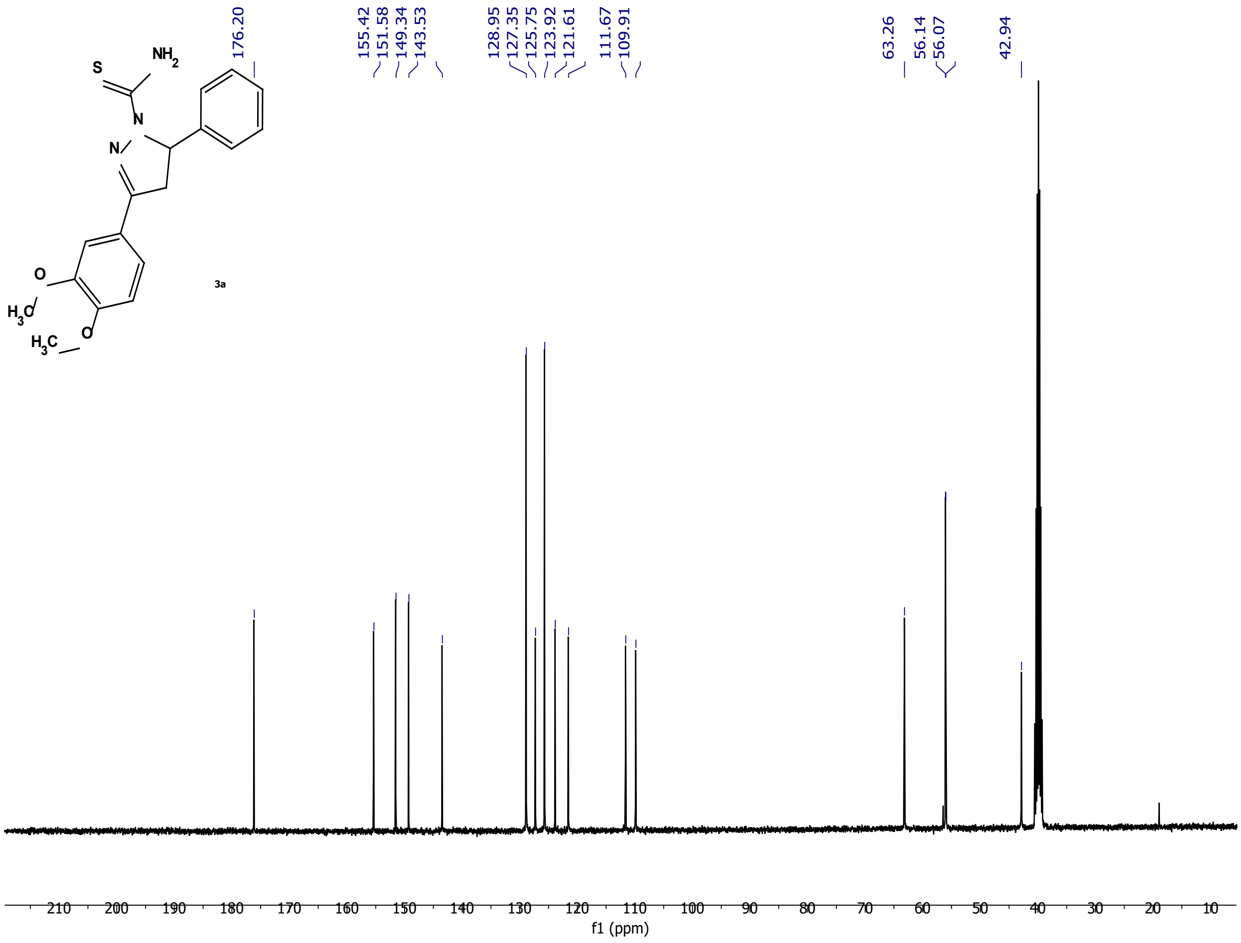

Figure S7: ^{13}C NMR of compound 3a

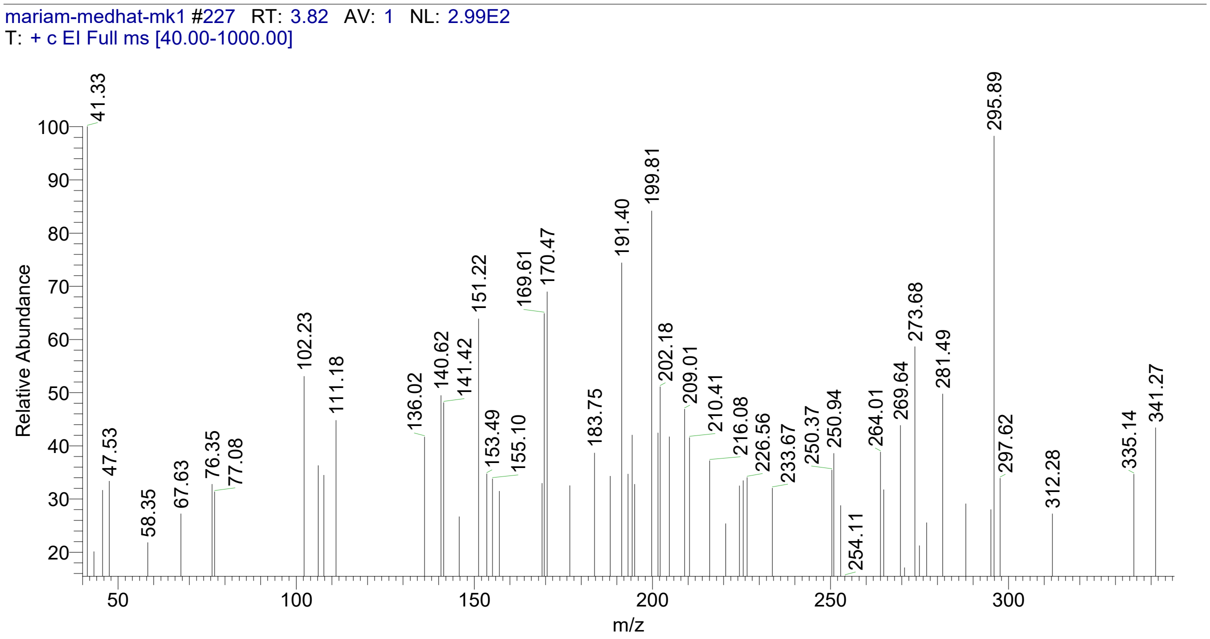

Figure S8: Mass of compound 3a

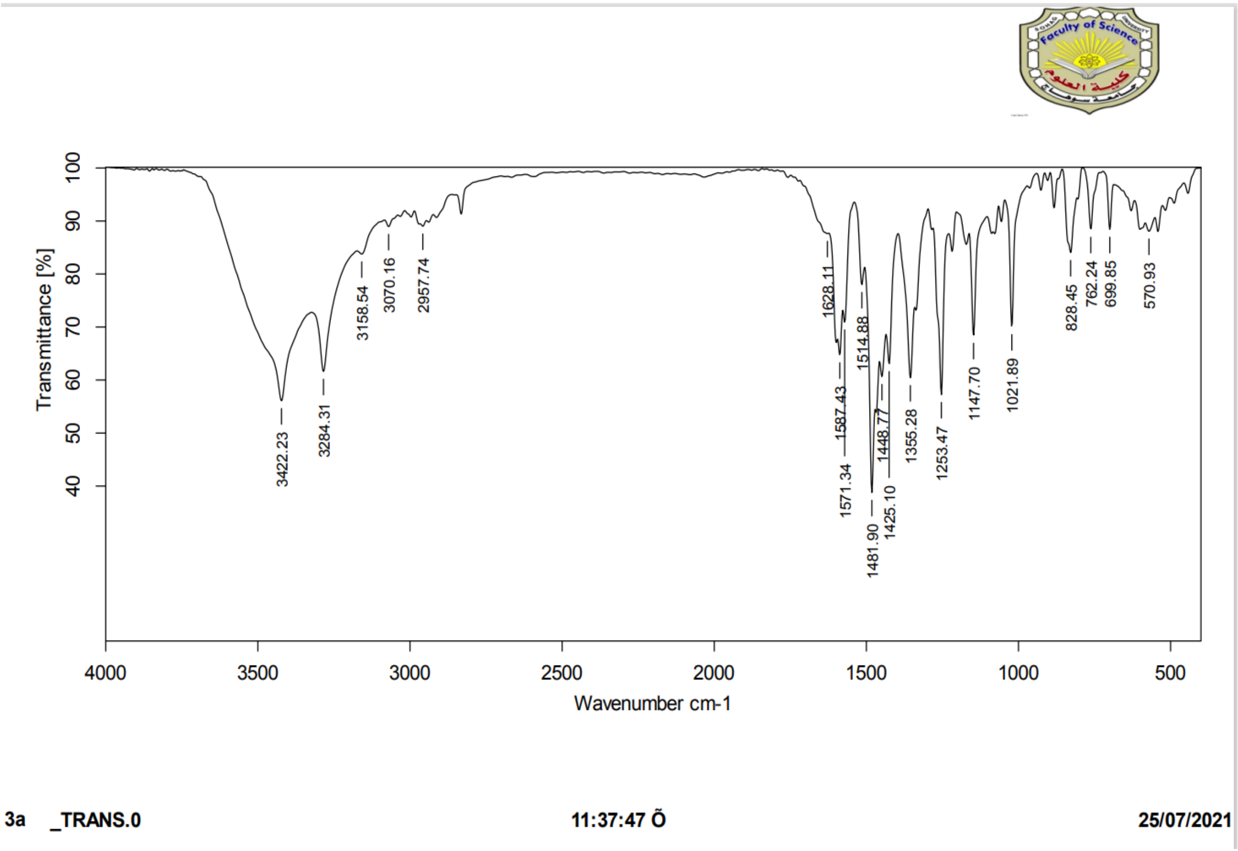

Figure S9: IR of compound 3a

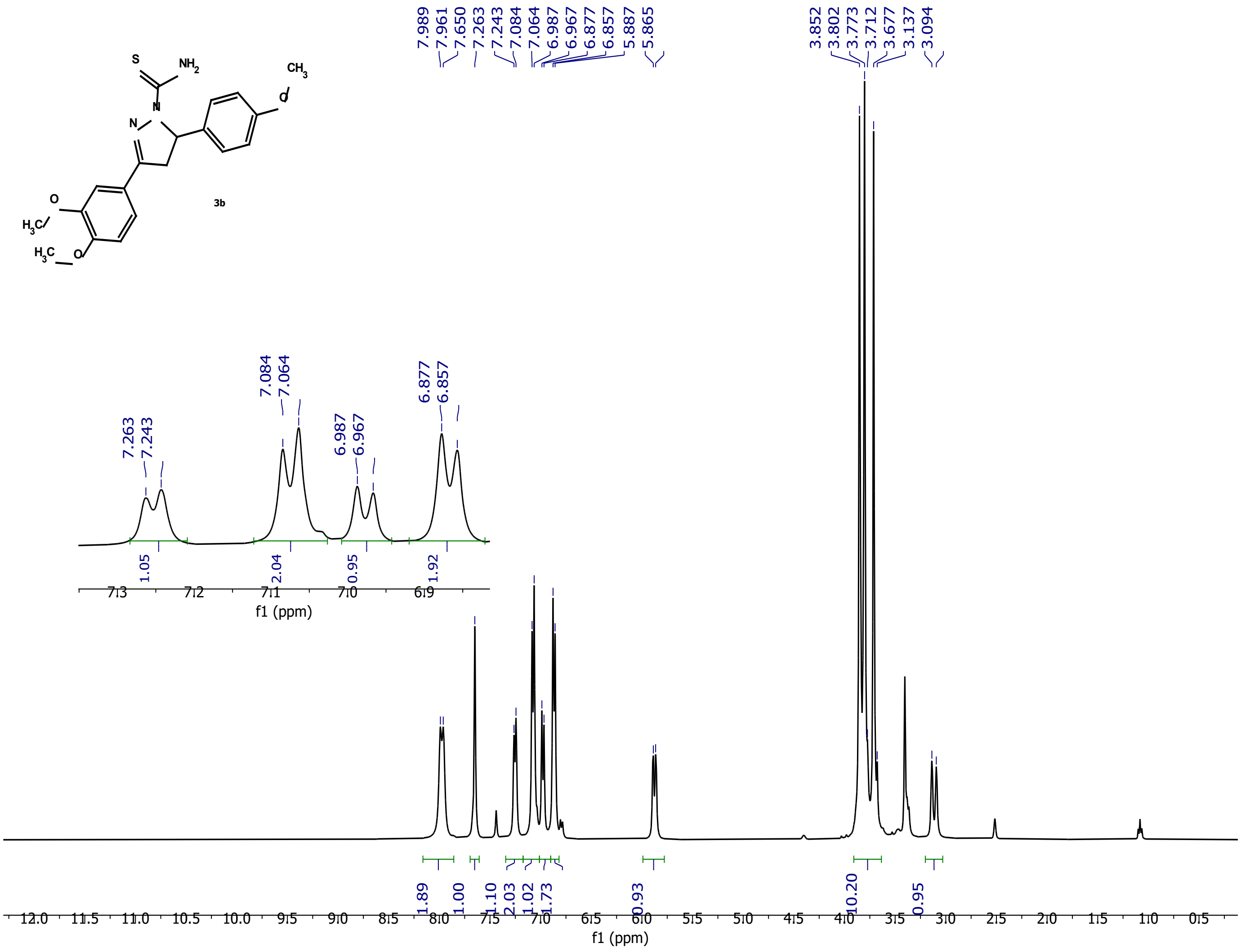

Figure S10: ^{1}H NMR of compound 3b

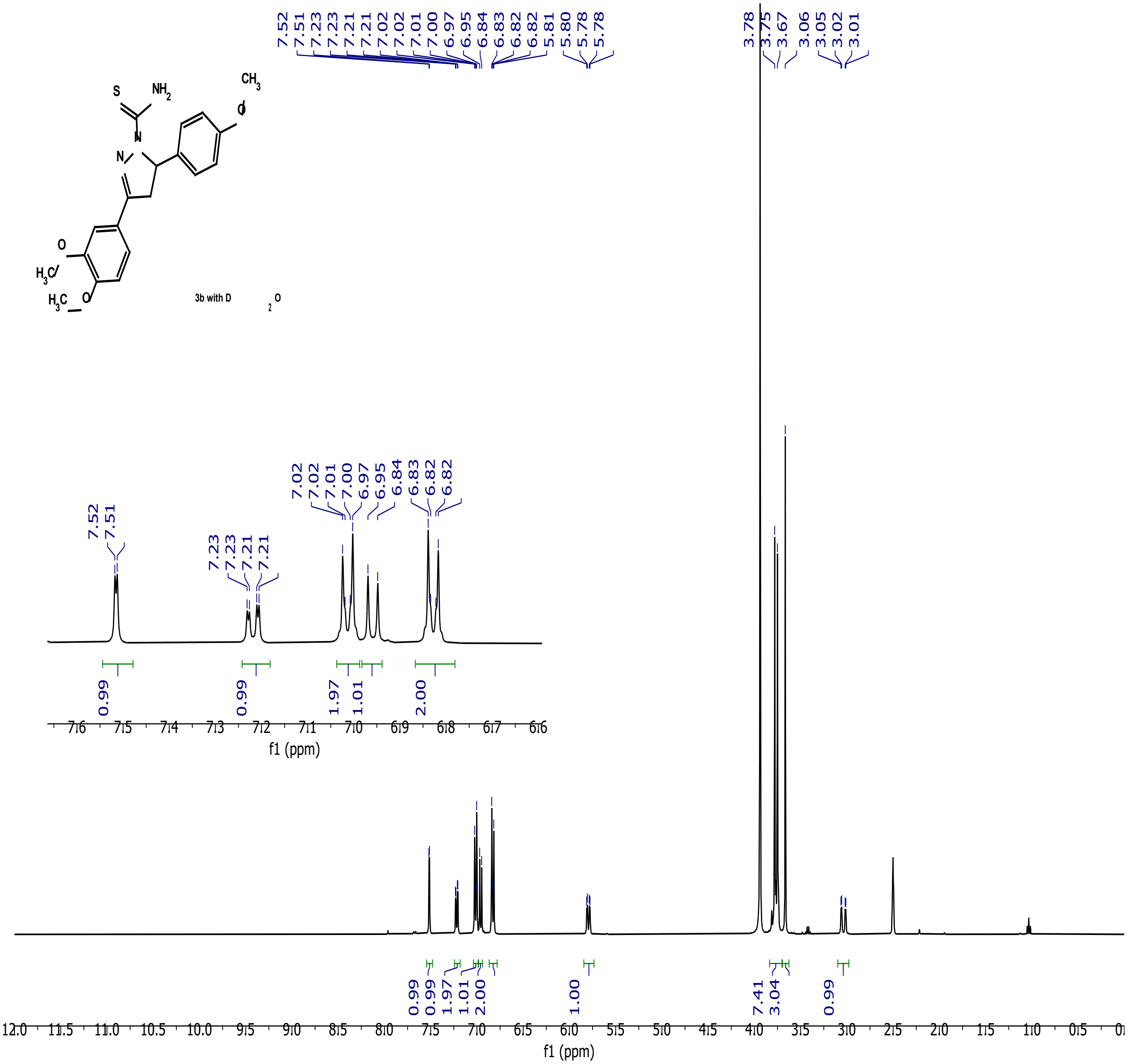

Figure S11: ^{1}H NMR (D_2O) of compound 3b

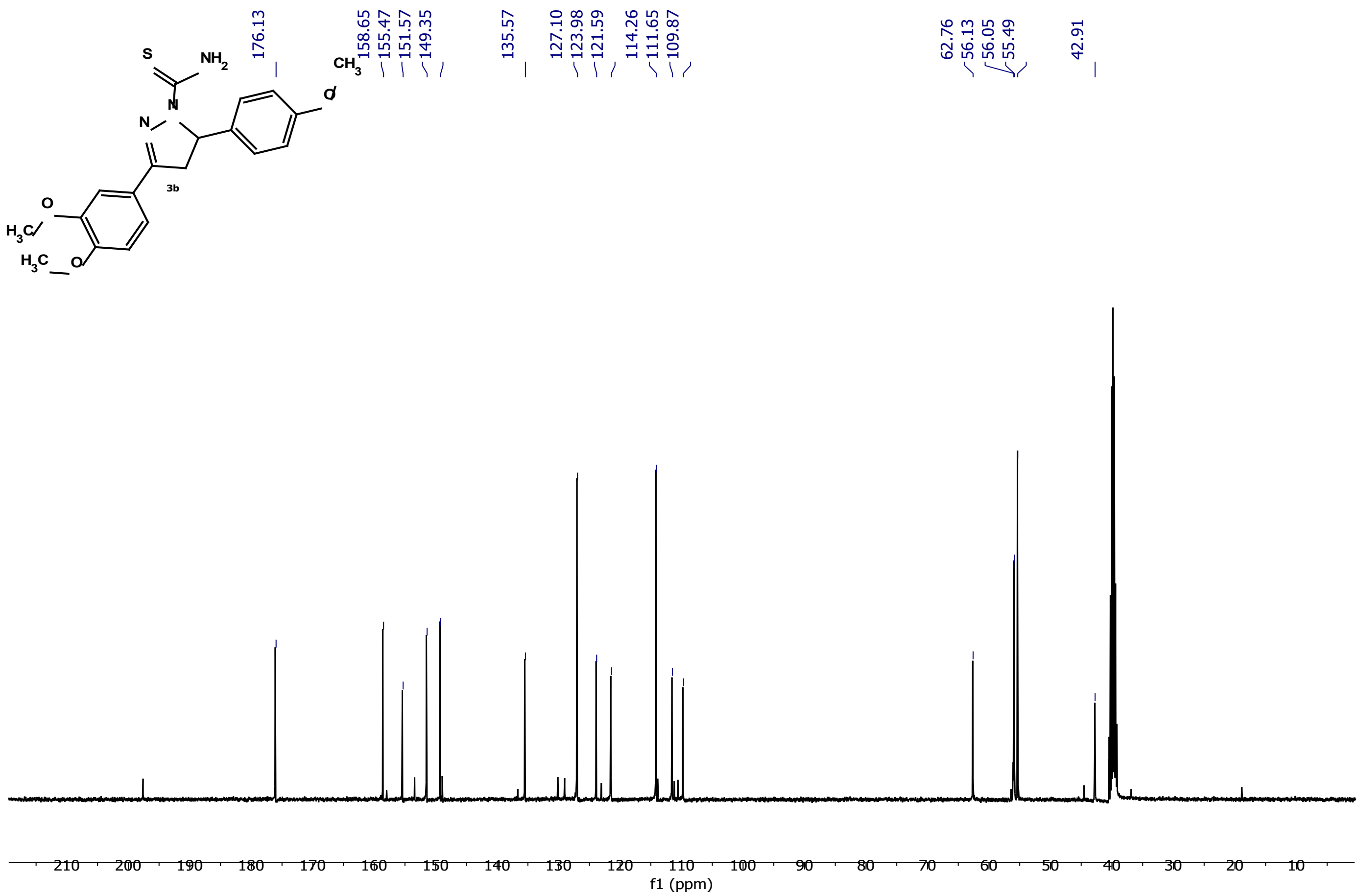

Figure S12: ^{13}C NMR of compound 3b

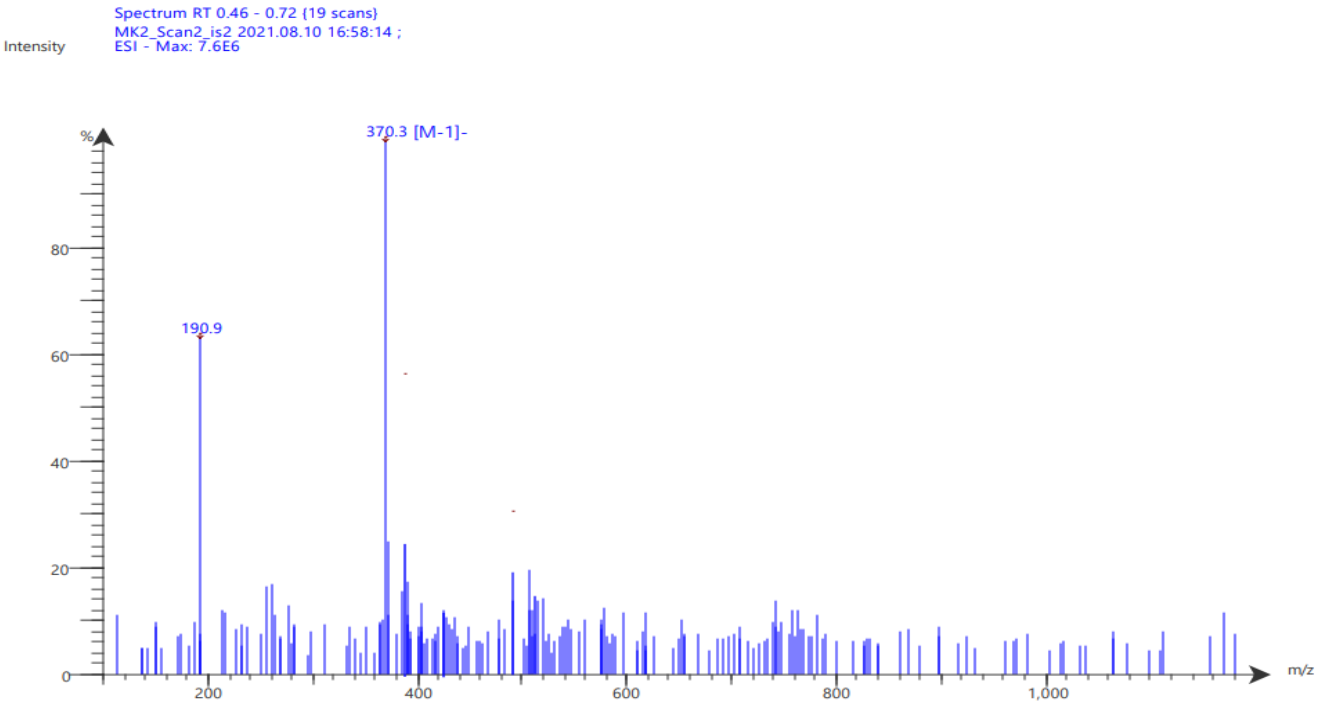

Figure S13: Mass of compound 3b

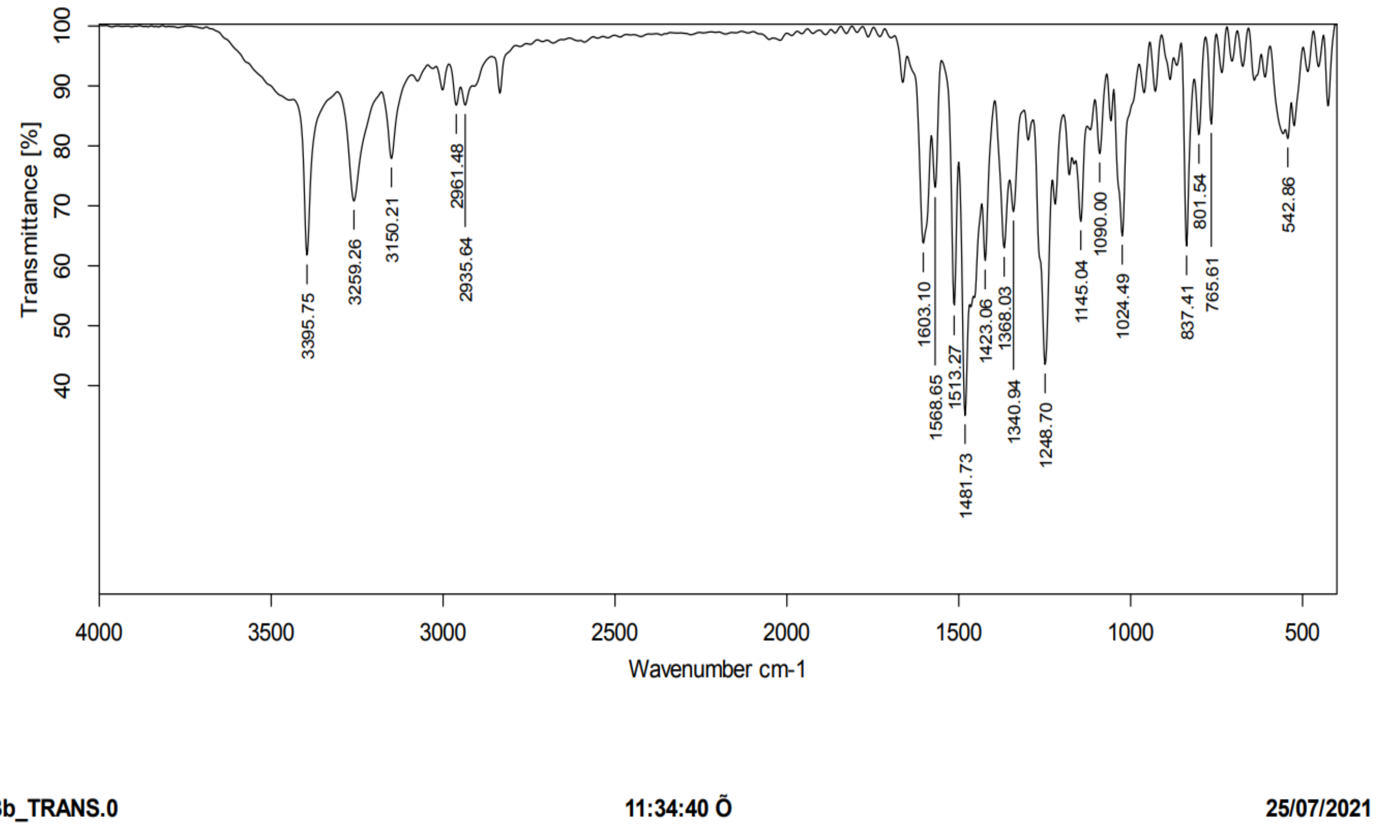

Figure S14: IR of compound 3b

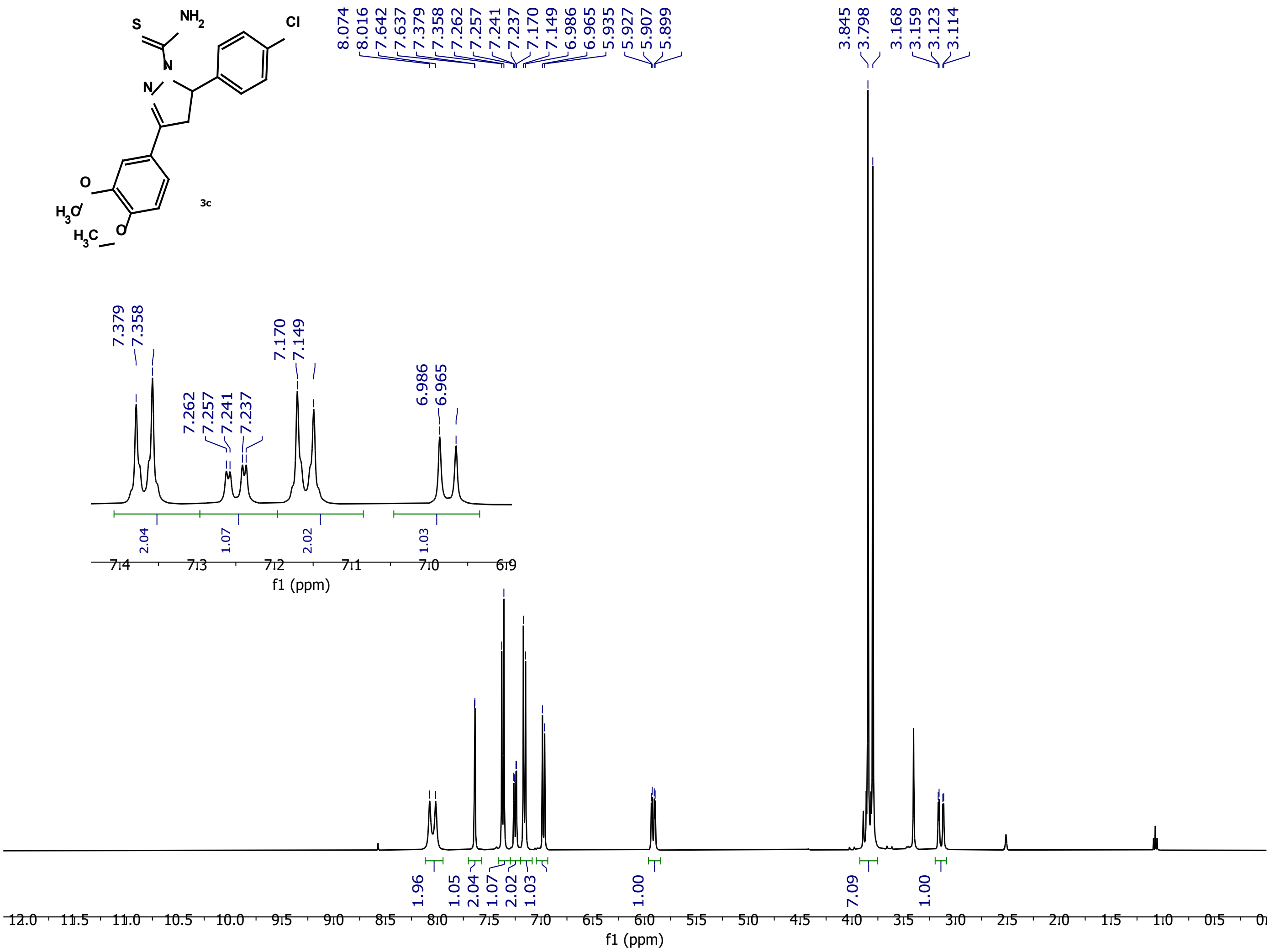

Figure S15: ^{1}H NMR of compound 3c

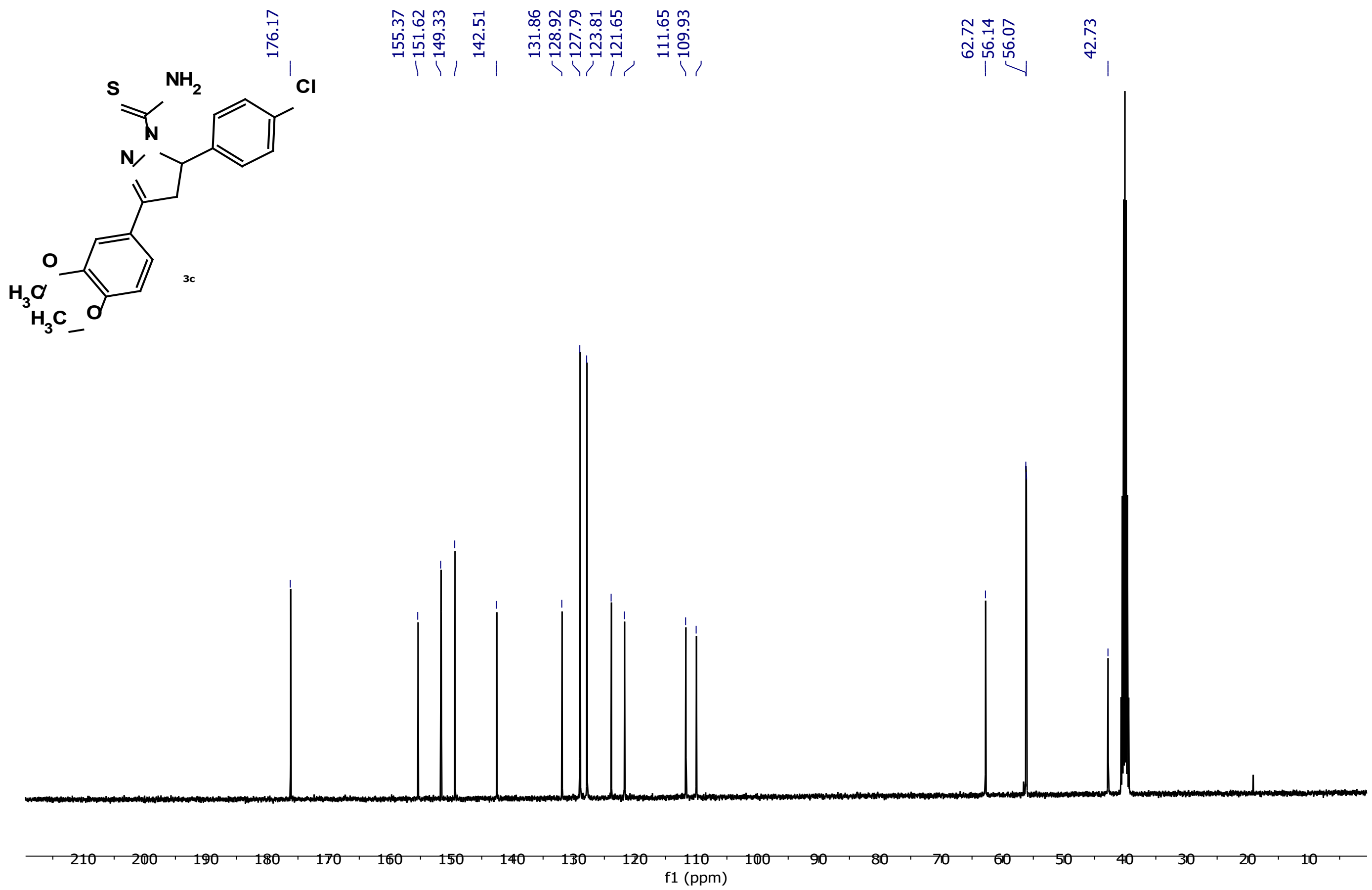

Figure S16: ^{13}C NMR of compound 3C

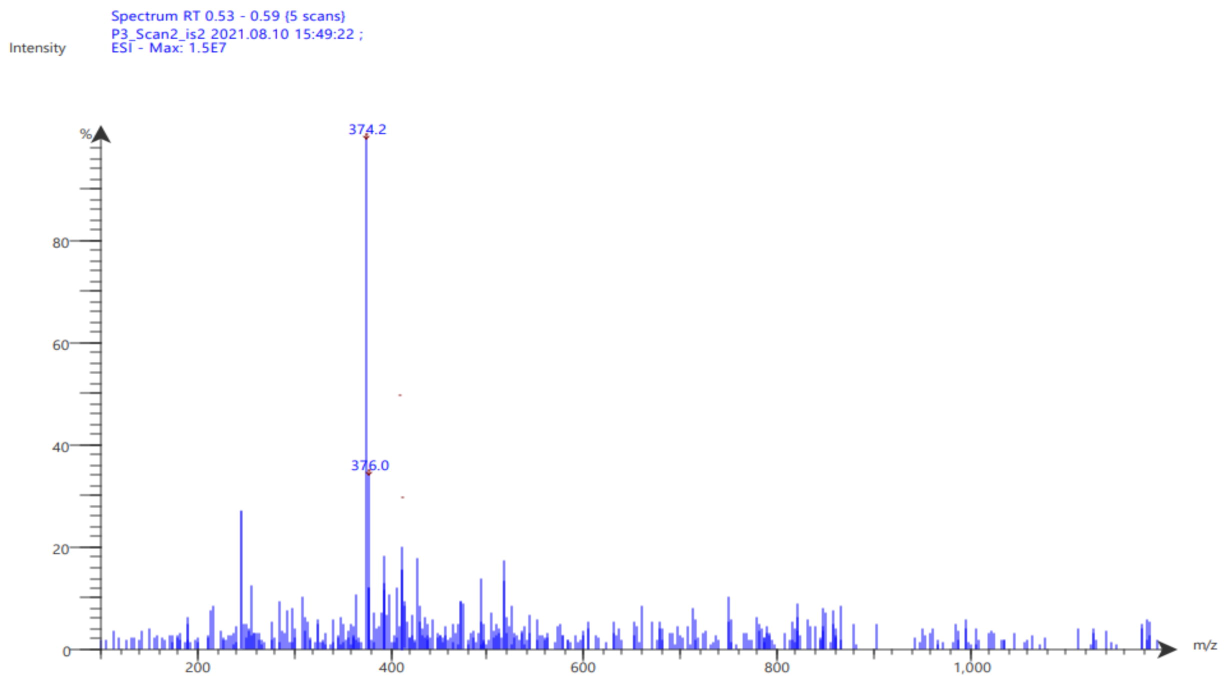

Figure S17: Mass of compound 3C

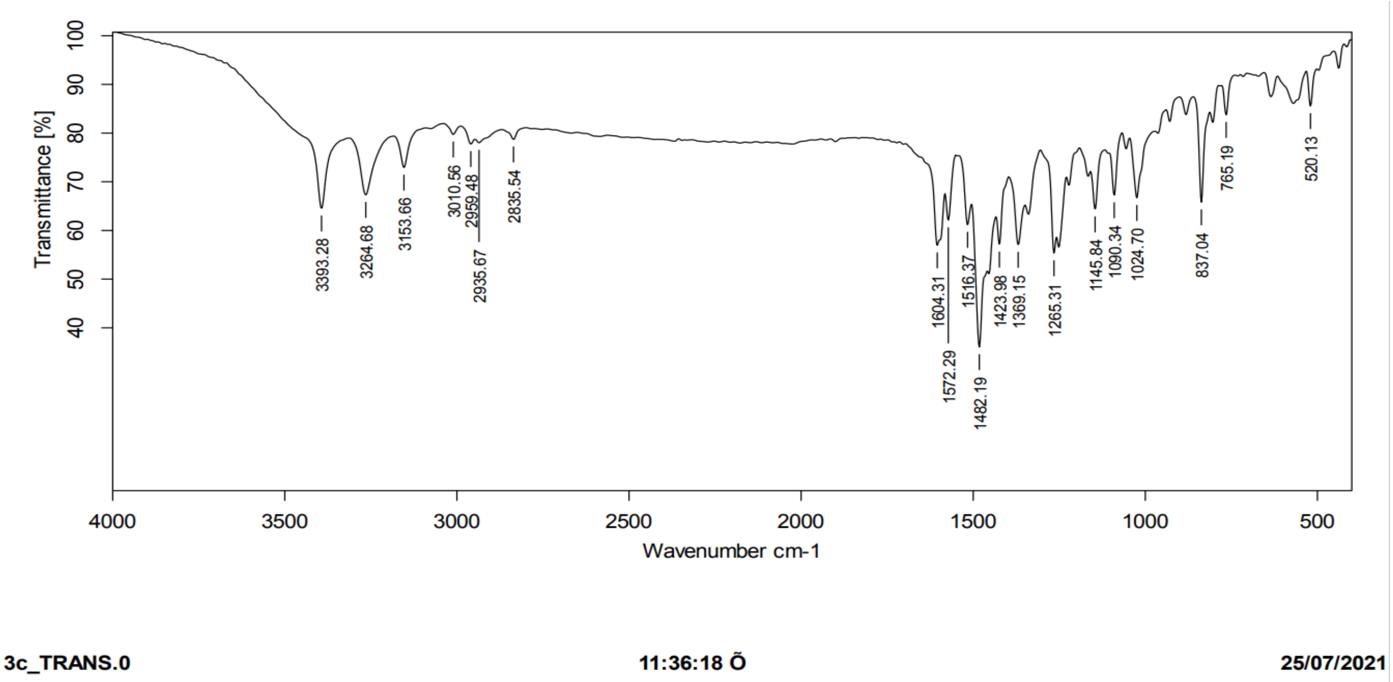

Figure S18: IR of compound 3C

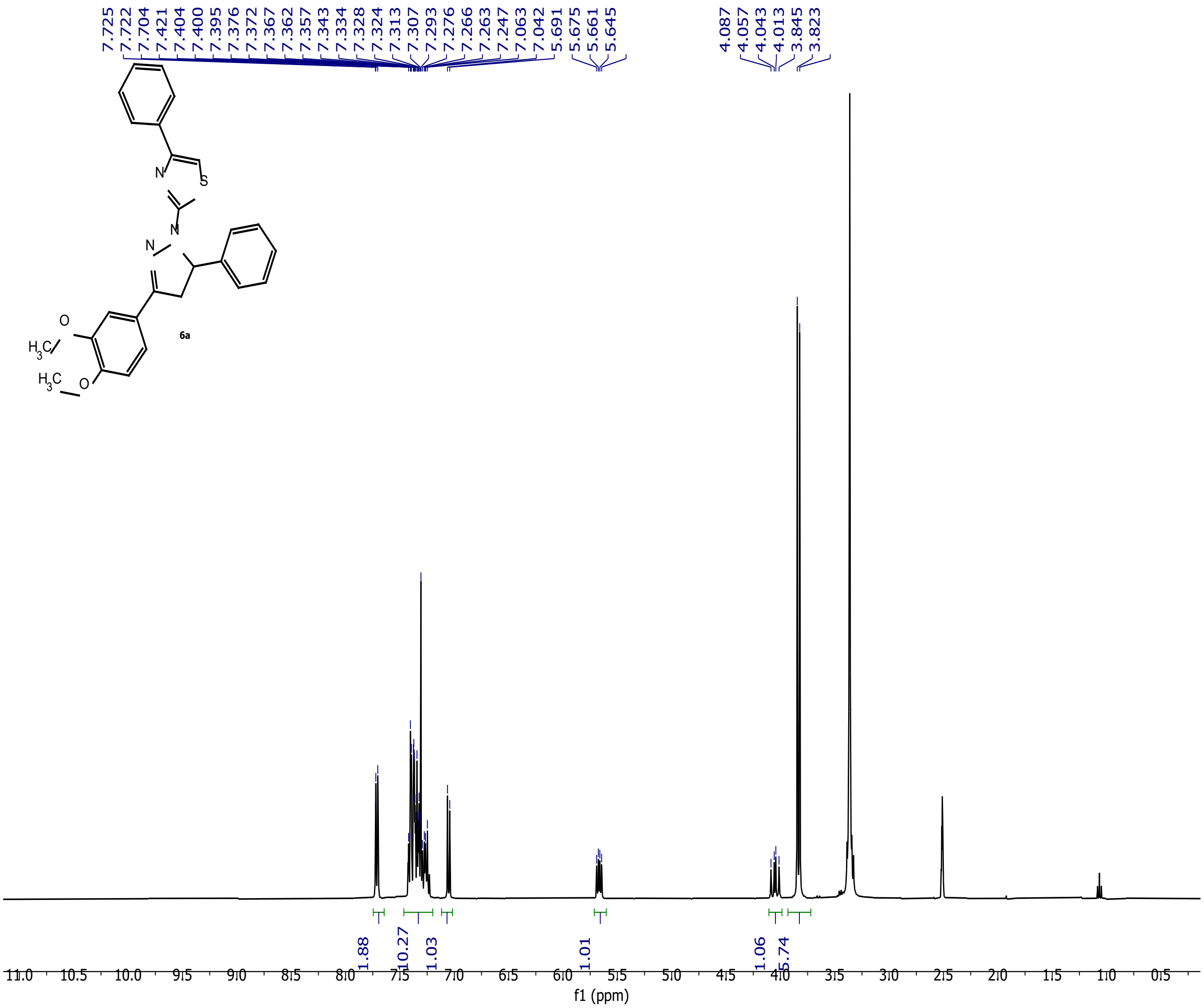

Figure S19: ^{1}H NMR of compound 6a

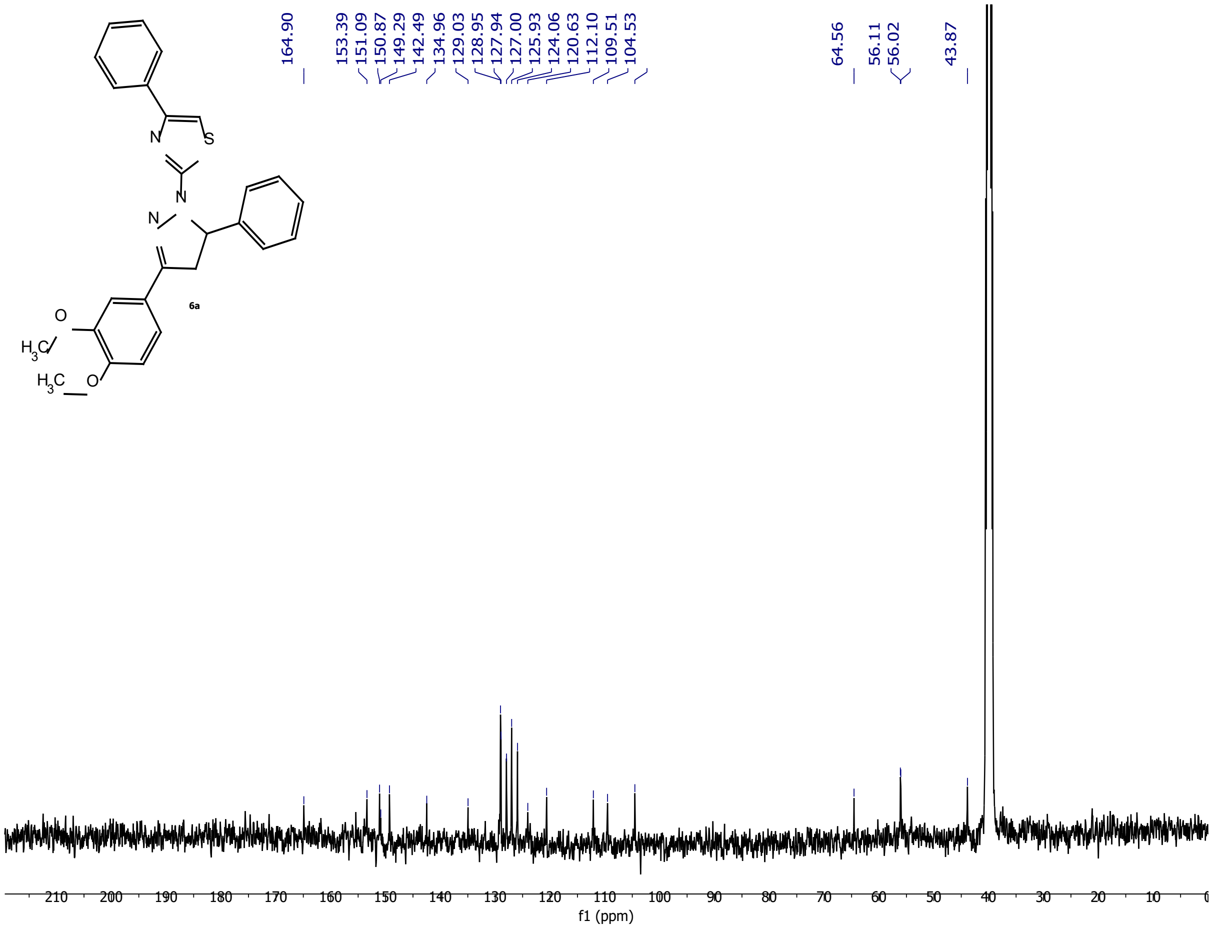

Figure S20: ^{13}C NMR of compound 6a

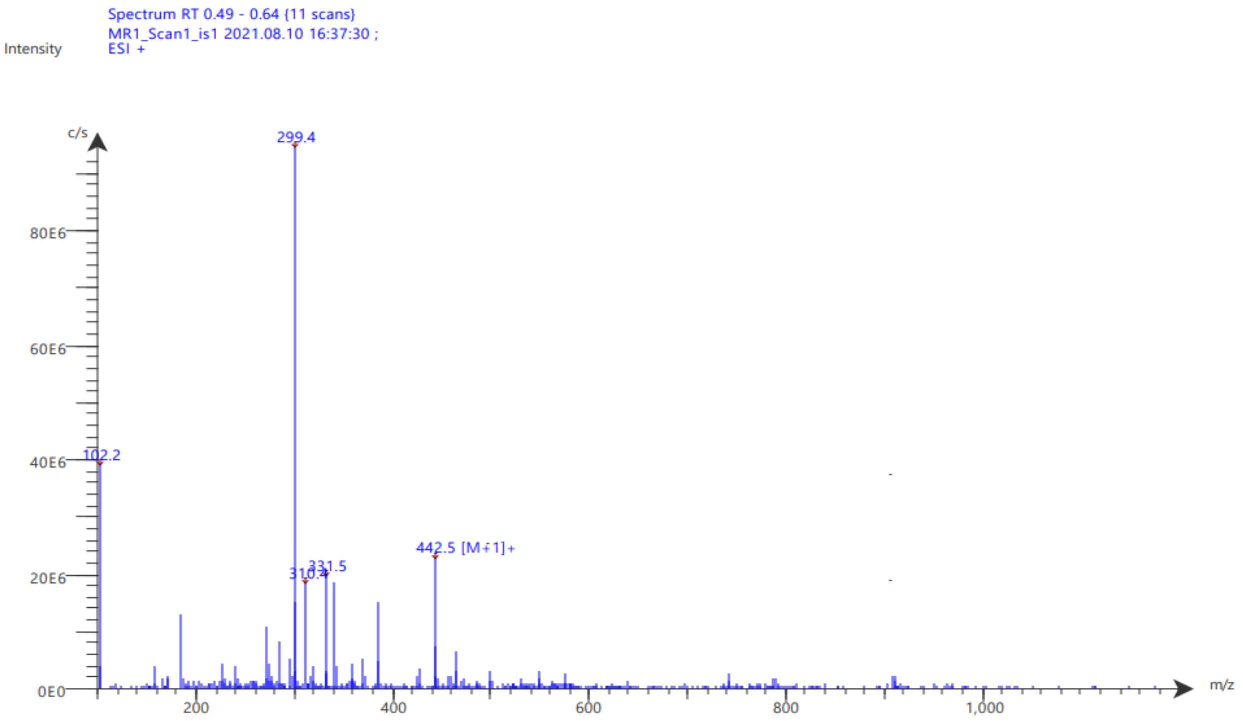

Figure S21: Mass of compound 6a

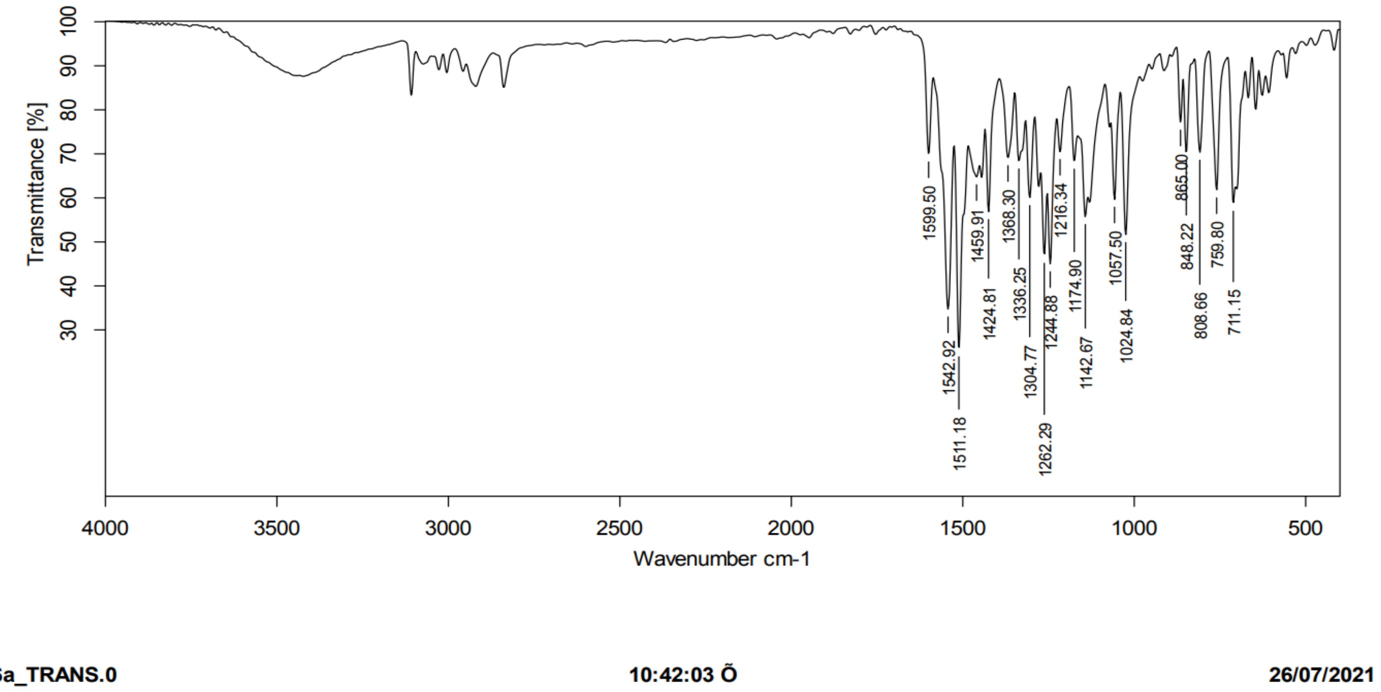

Figure S22: IR of compound 6a

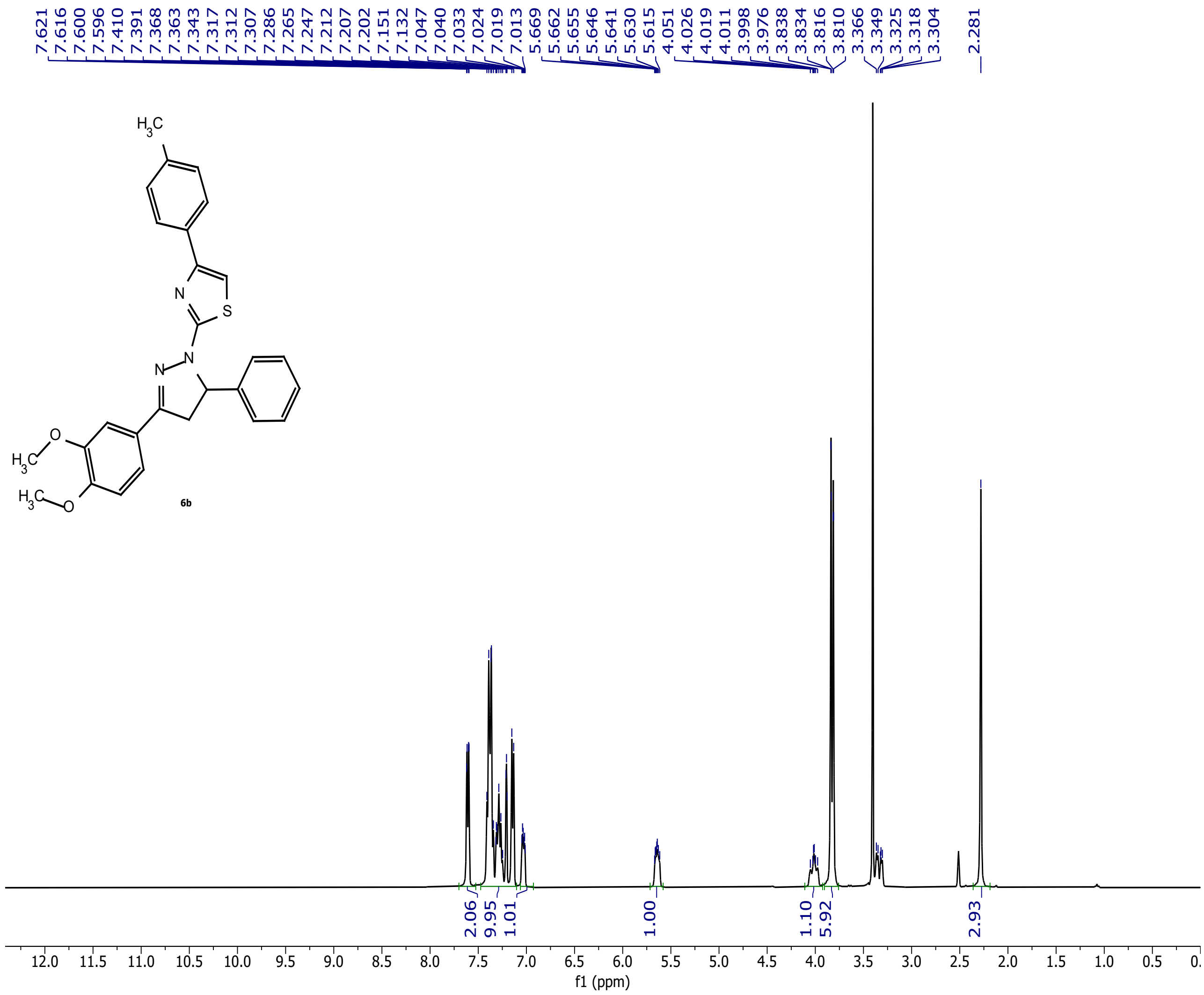

Figure S23: ^{1}H NMR of compound 6b

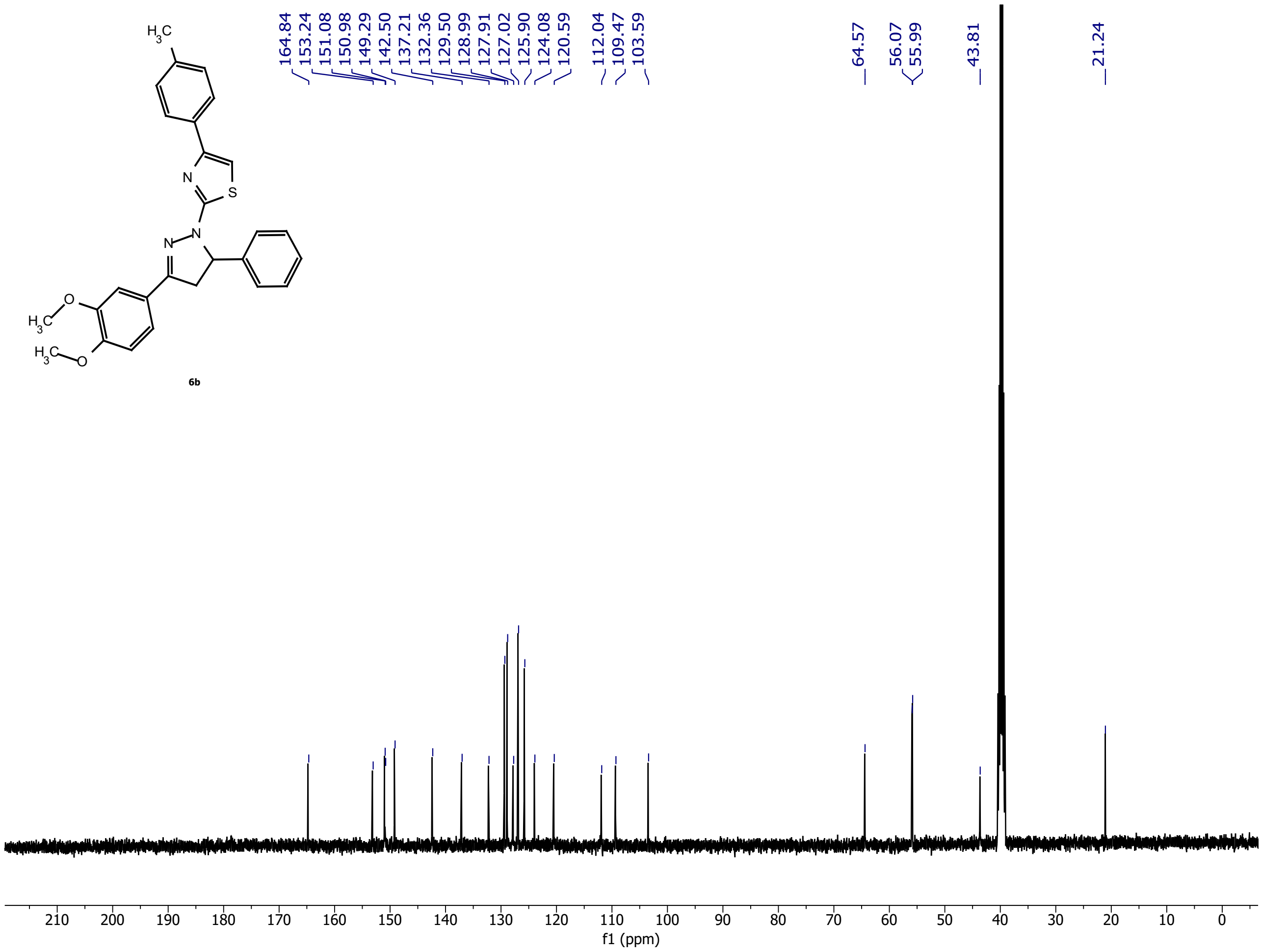

Figure S24: ^{13}C NMR of compound 6b

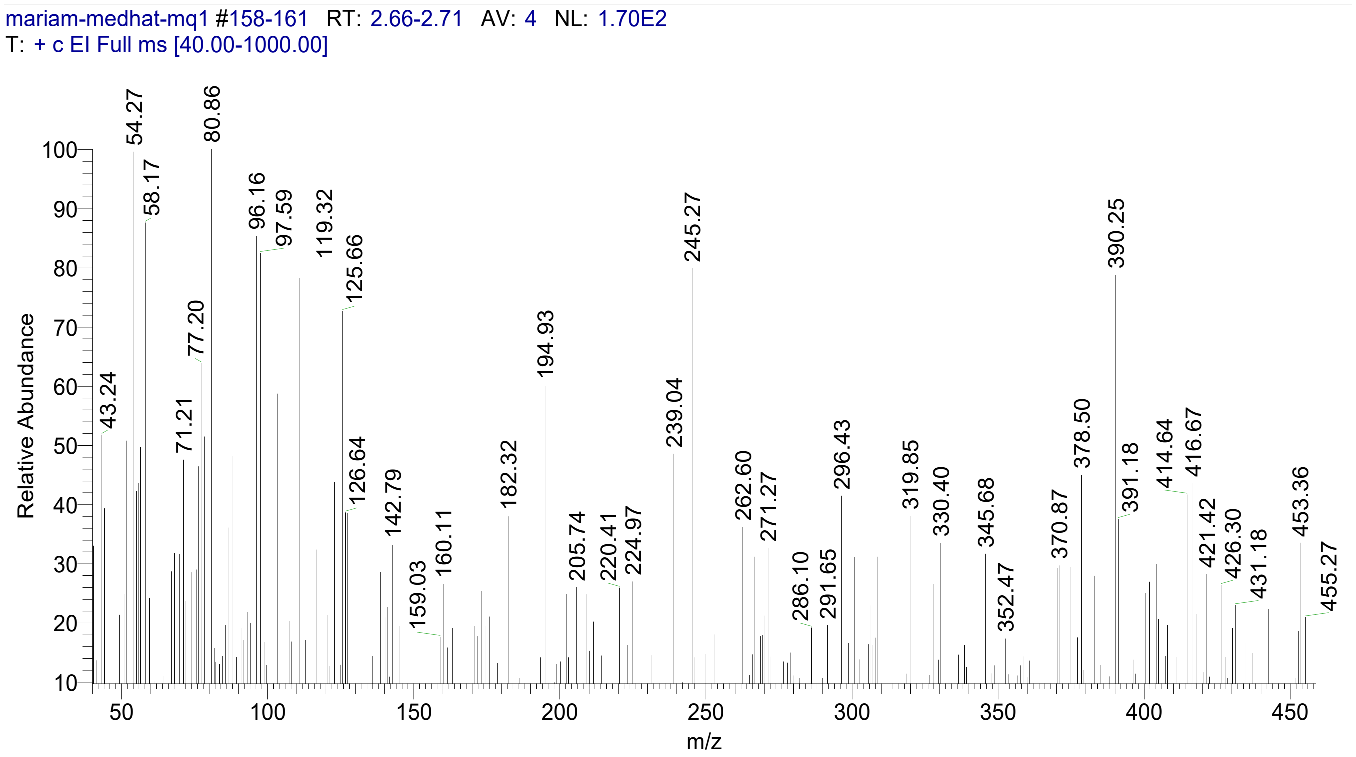

Figure S25: Mass of compound 6b

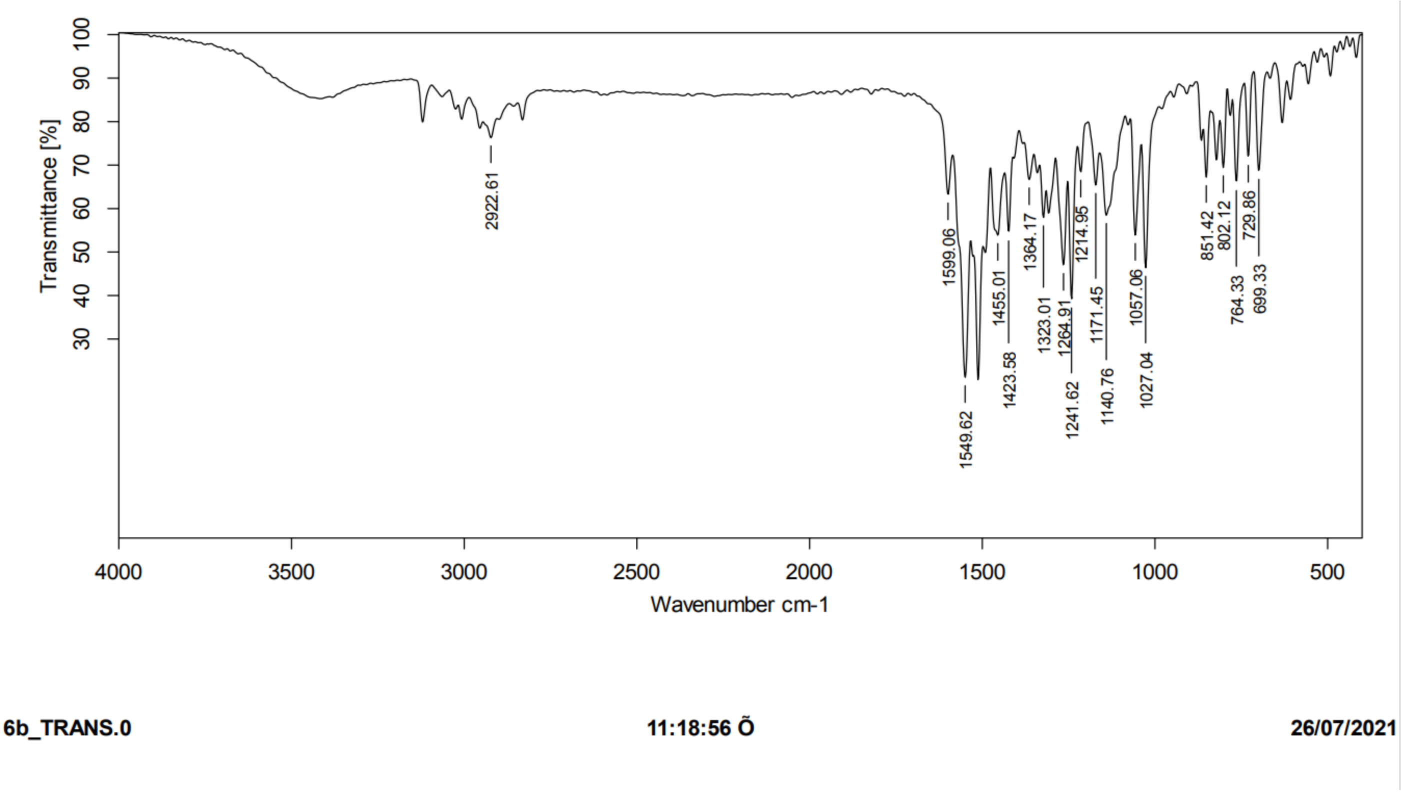

Figure S26: IR of compound 6b

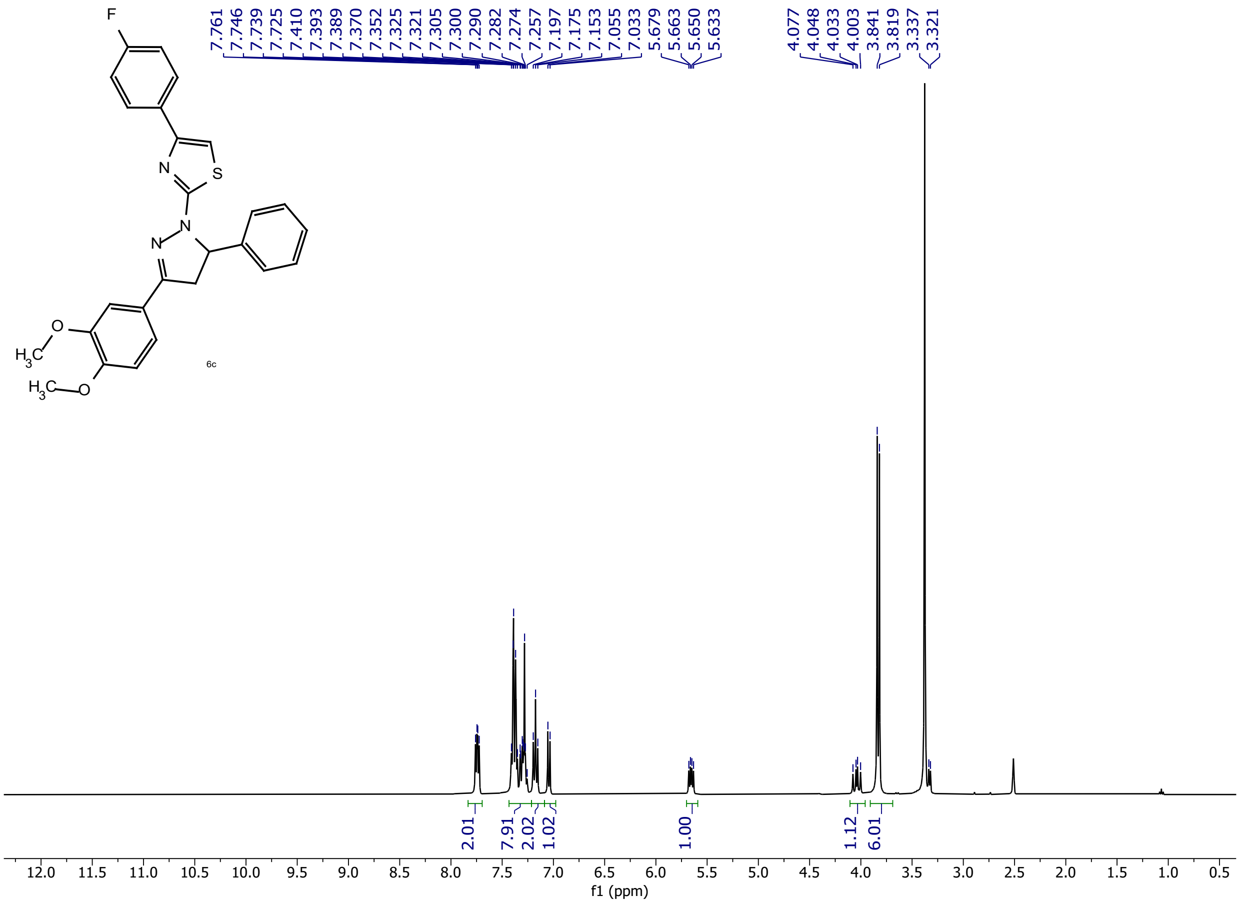

Figure S27: ^{1}H NMR of compound 6C

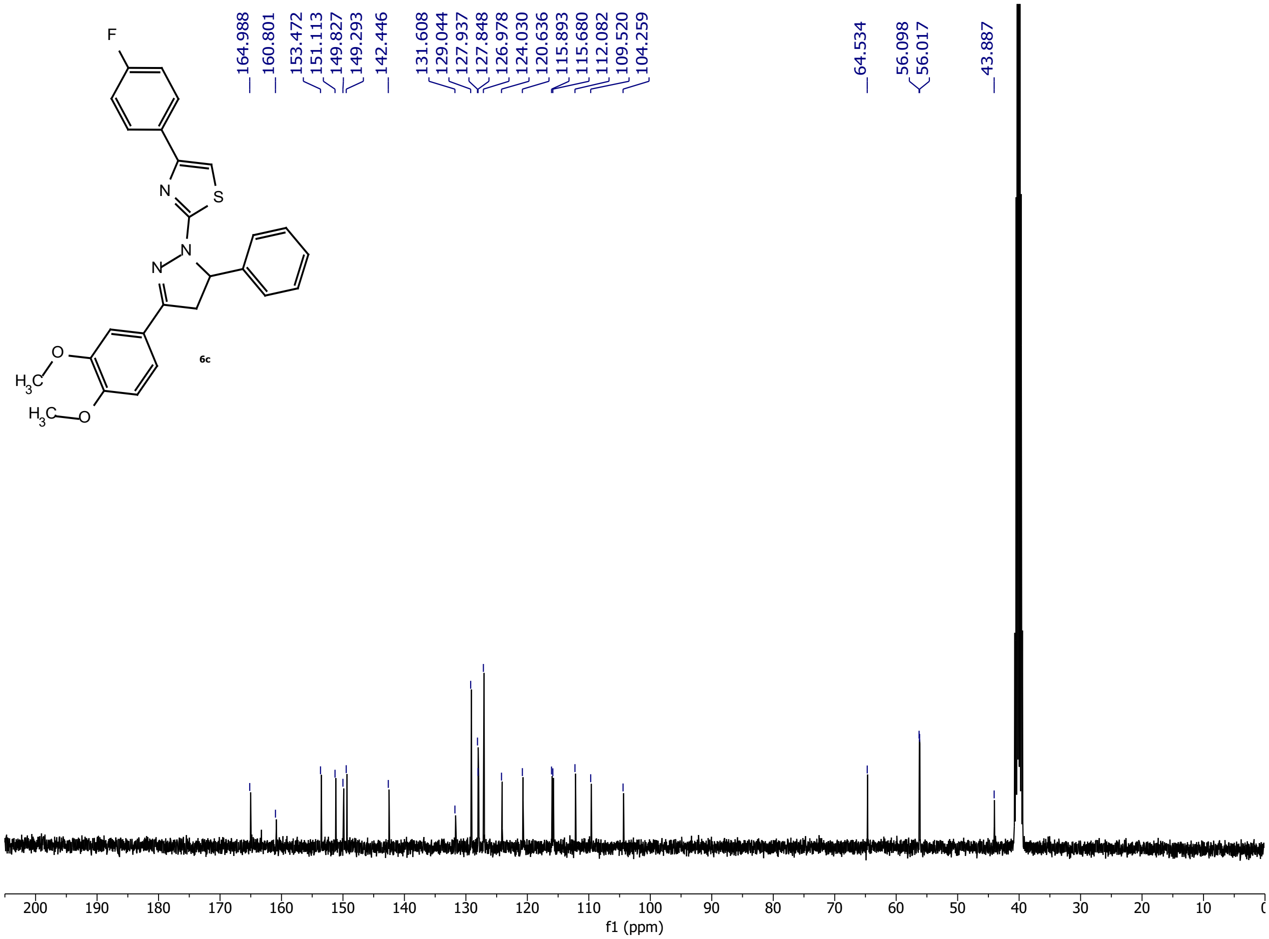

Figure S28: ^{13}C NMR of compound 6C

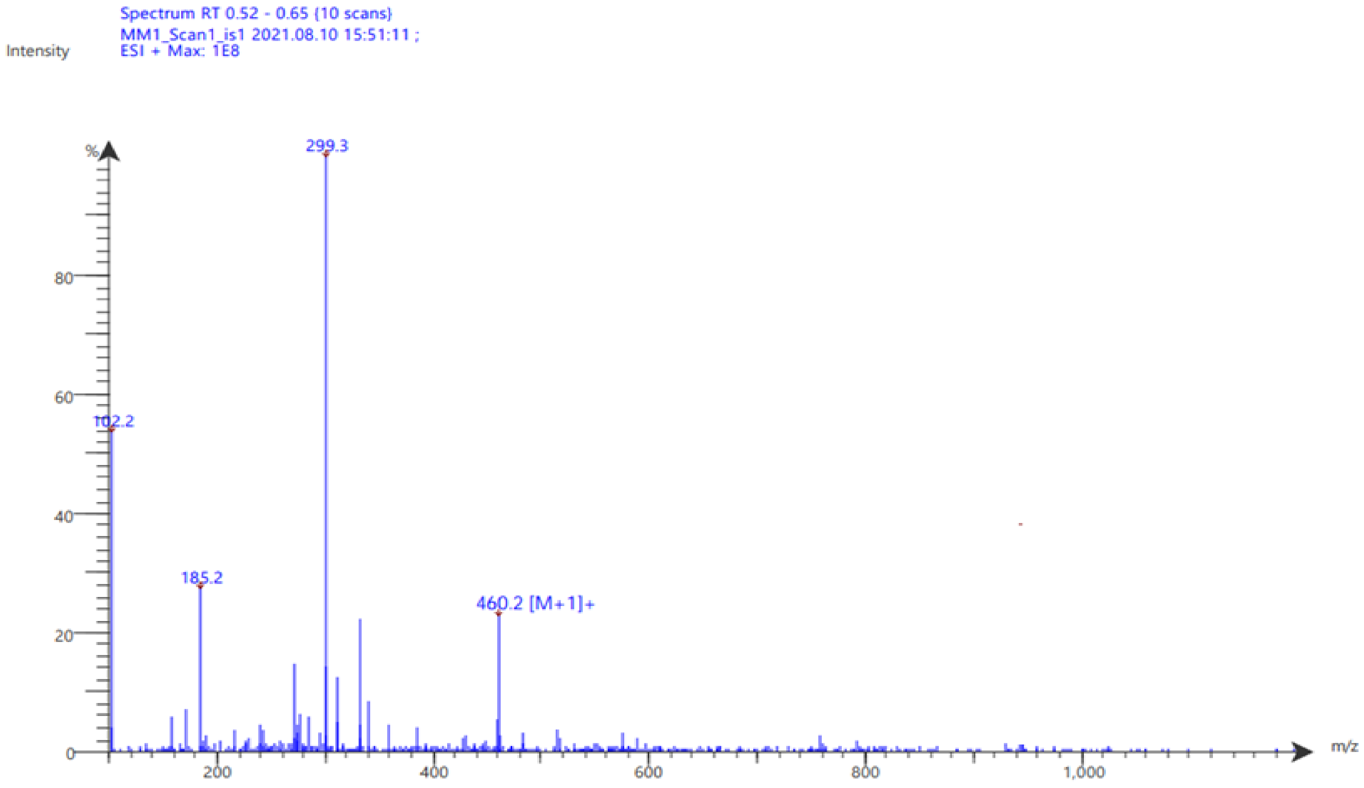

Figure S29: Mass of compound 6C

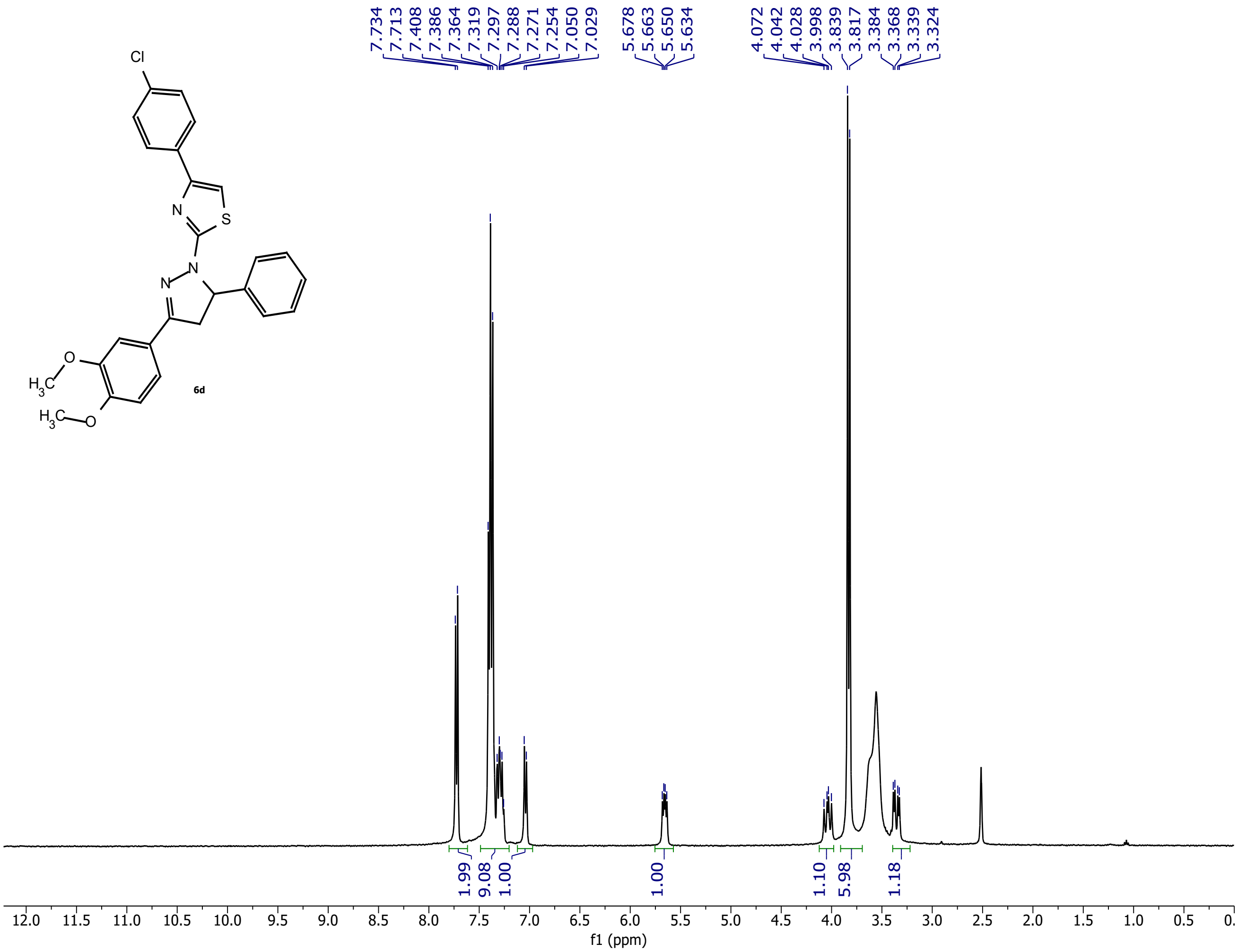

Figure S30: ^{1}H NMR of compound 6d

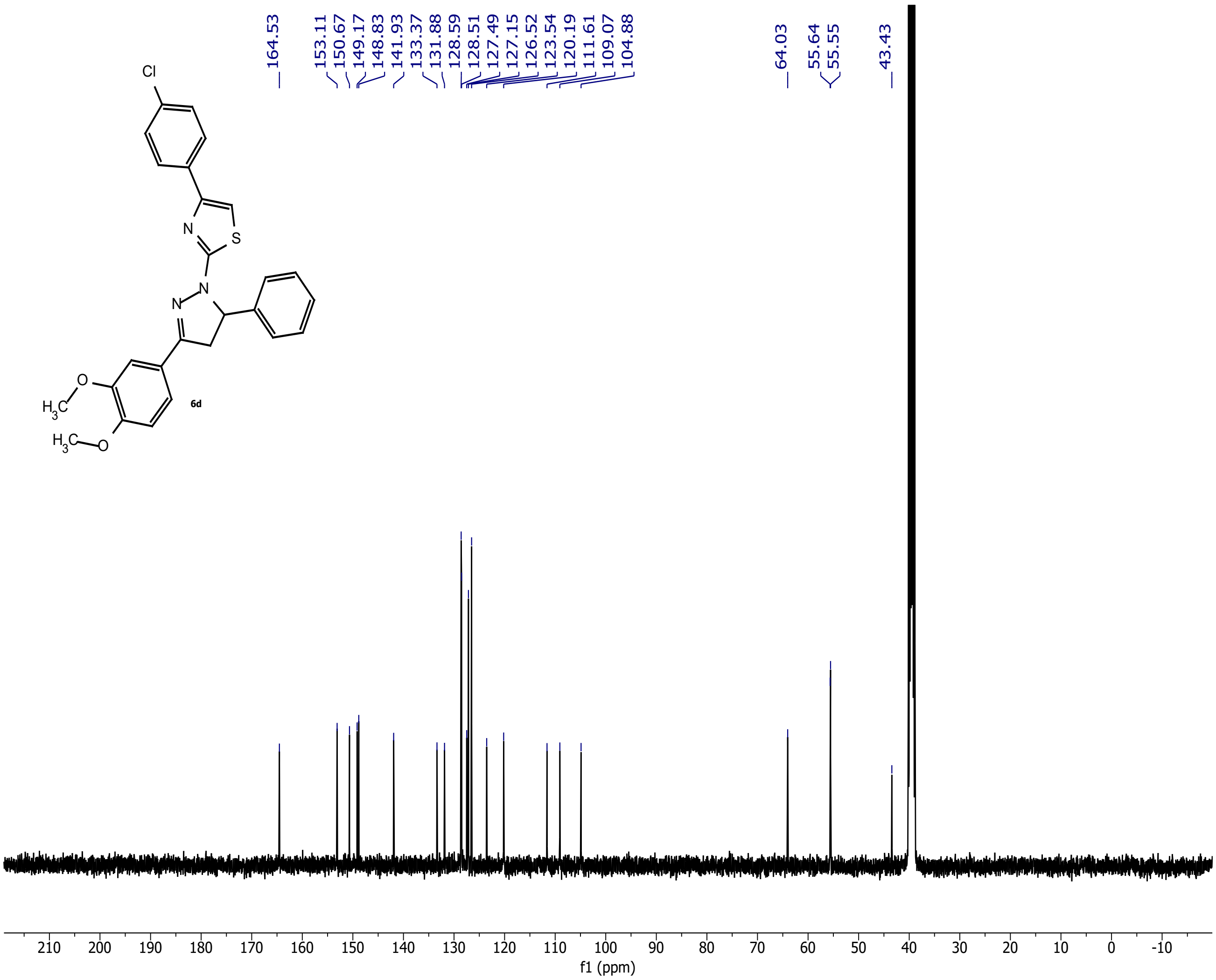

Figure S31: ^{13}C NMR of compound 6d

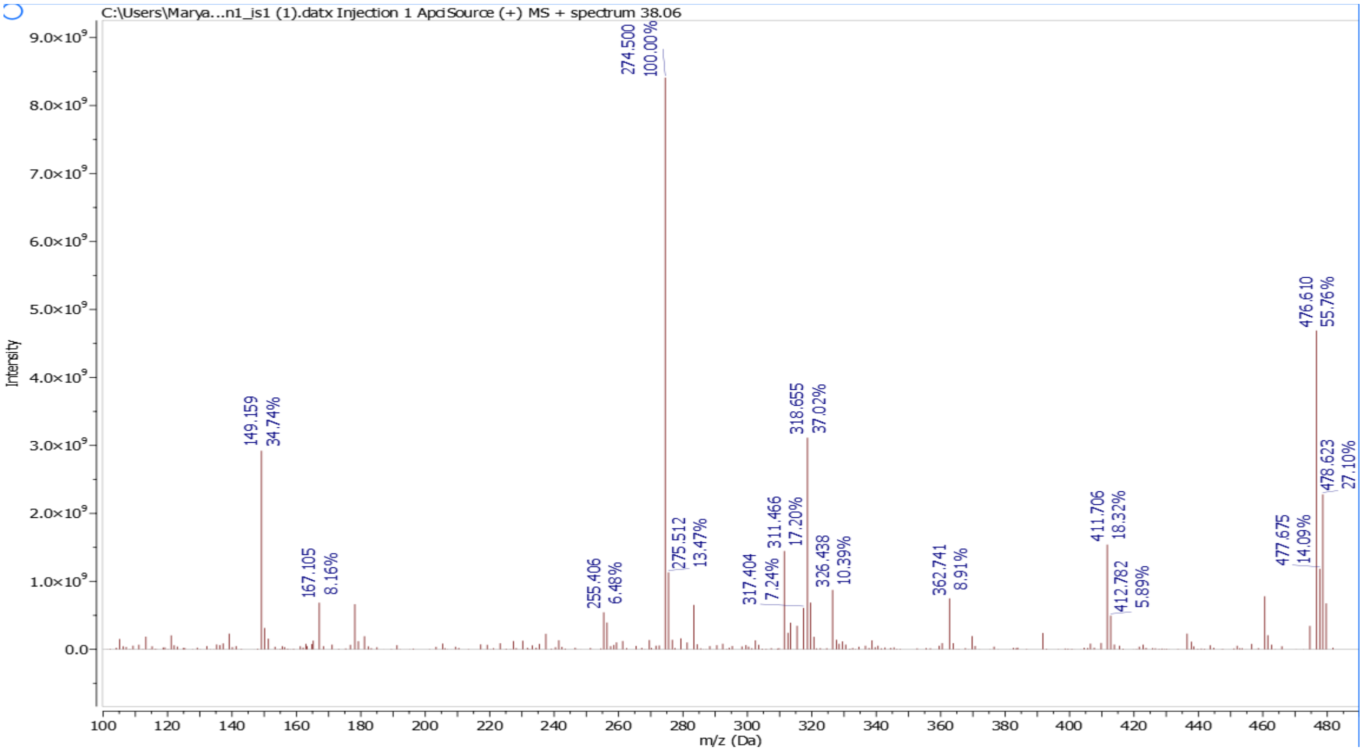

Figure S32: Mass of compound 6d

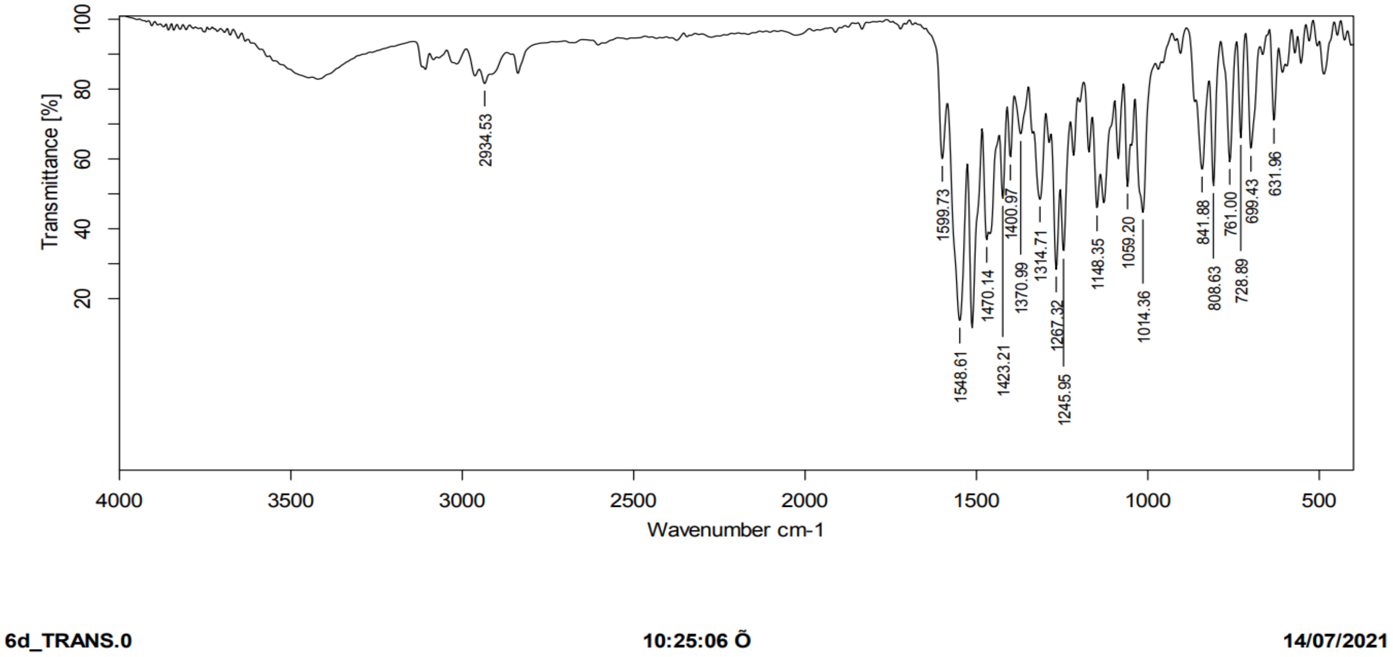

Figure S33: IR of compound 6d

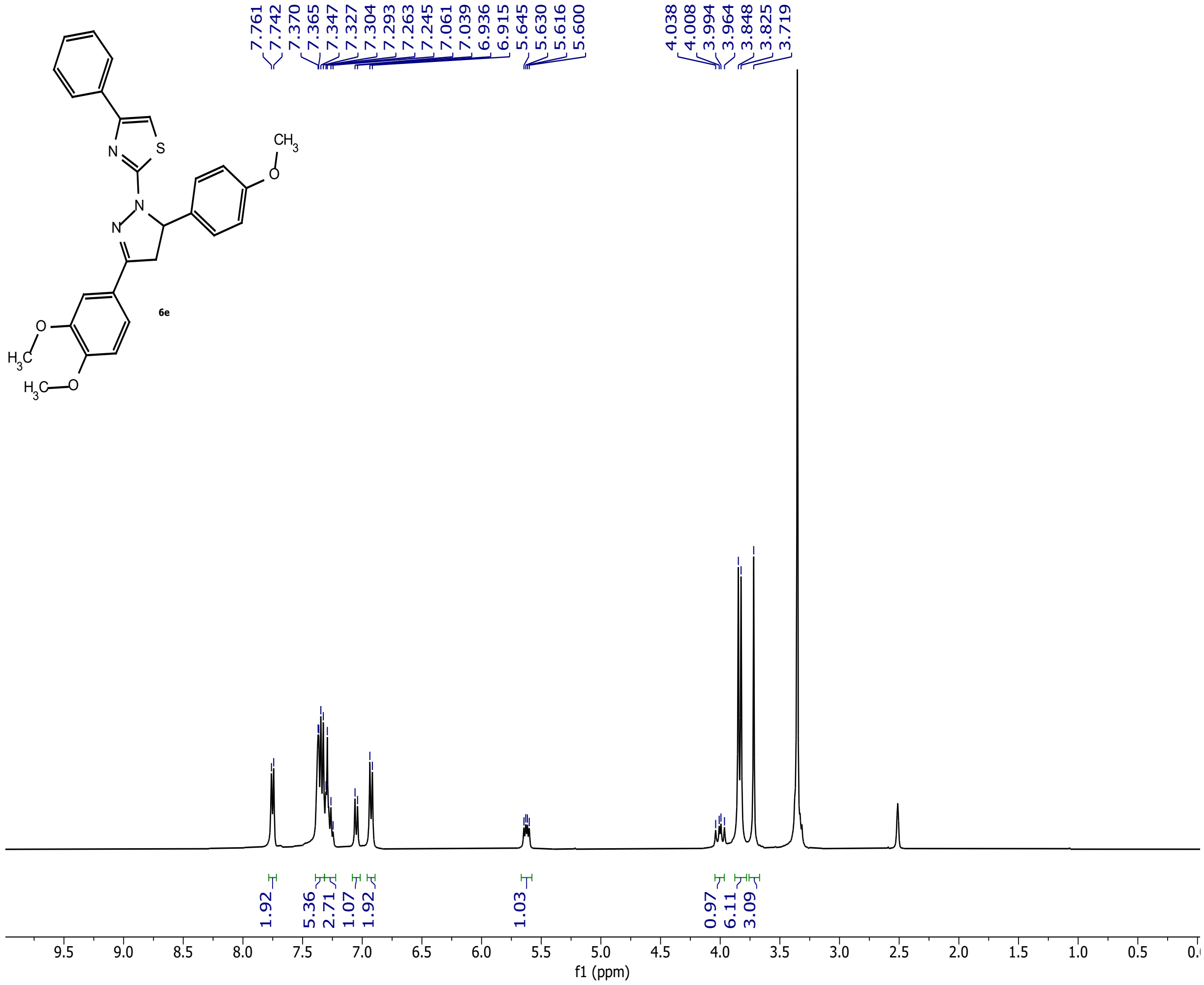

Figure S34: ^{1}H NMR of compound 6e

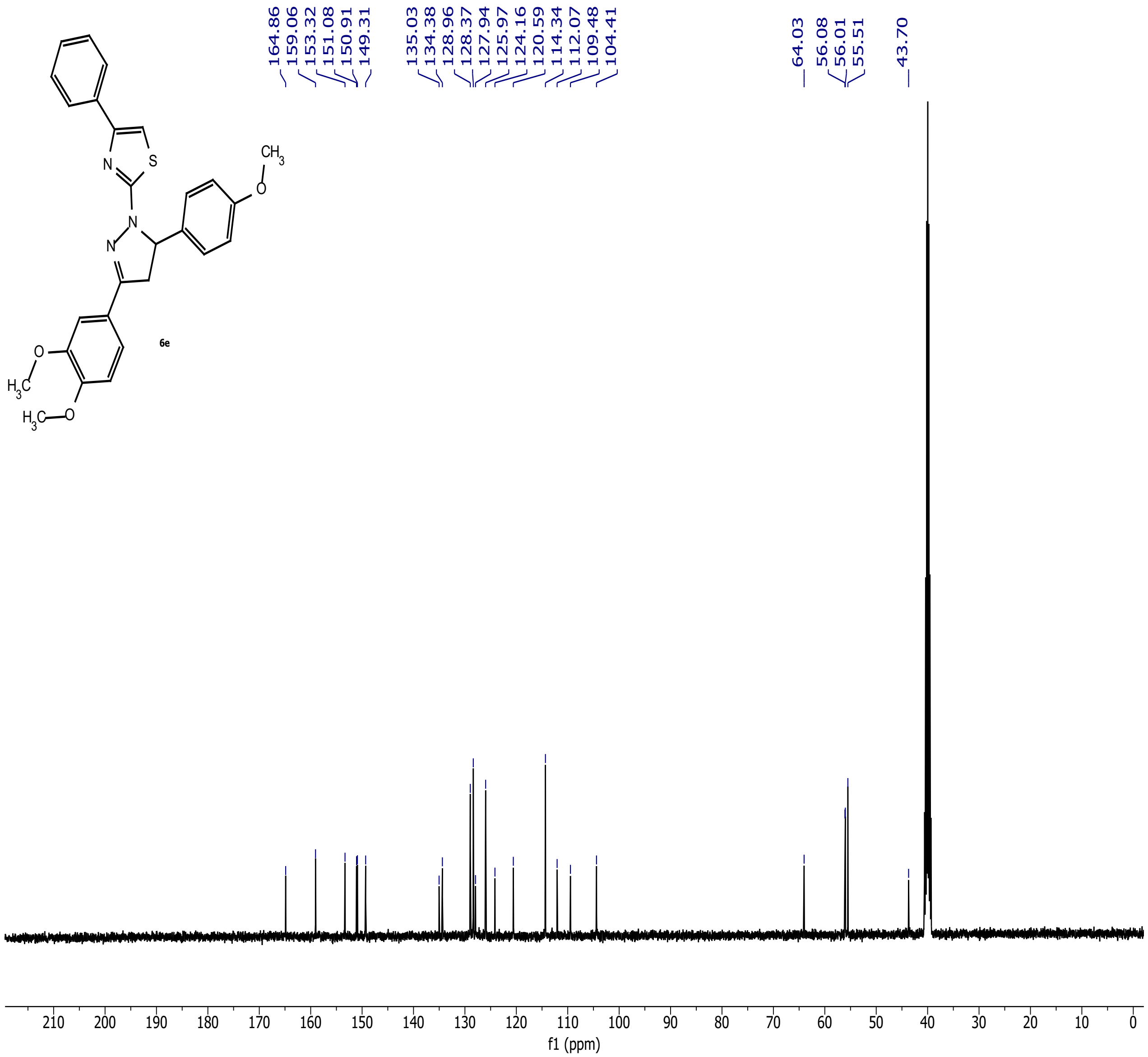

Figure S35: ^{13}C NMR of compound 6e

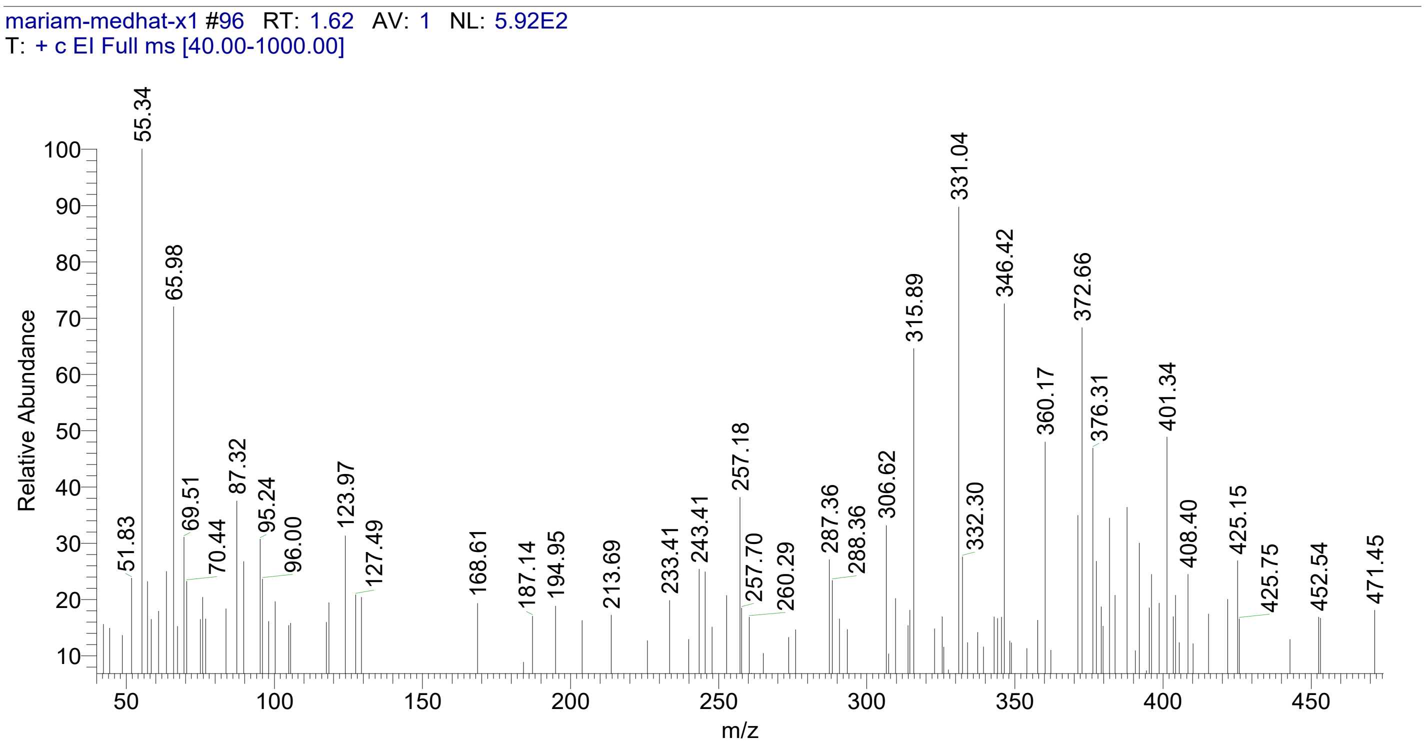

Figure S36: Mass of compound 6e

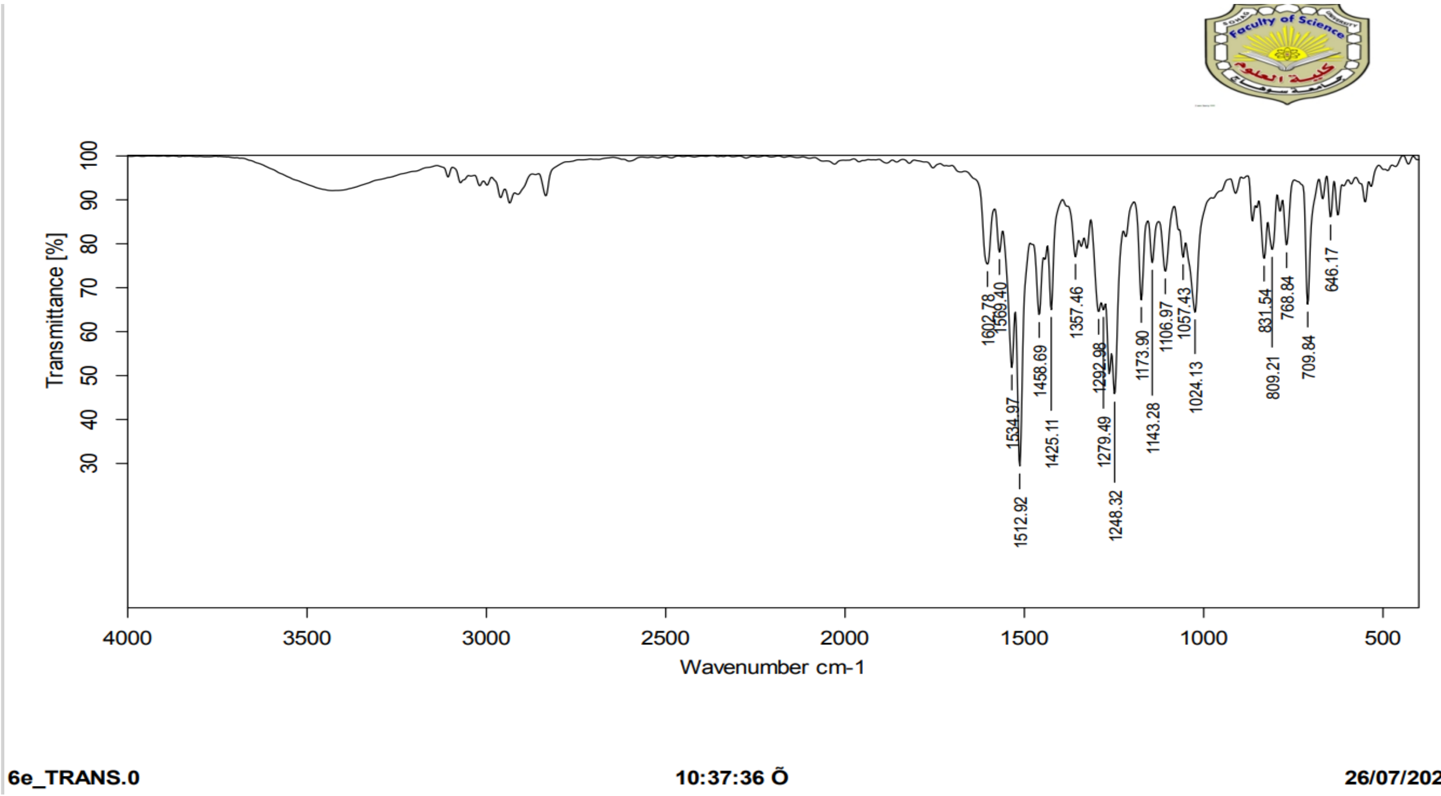

Figure S37: IR of compound 6e

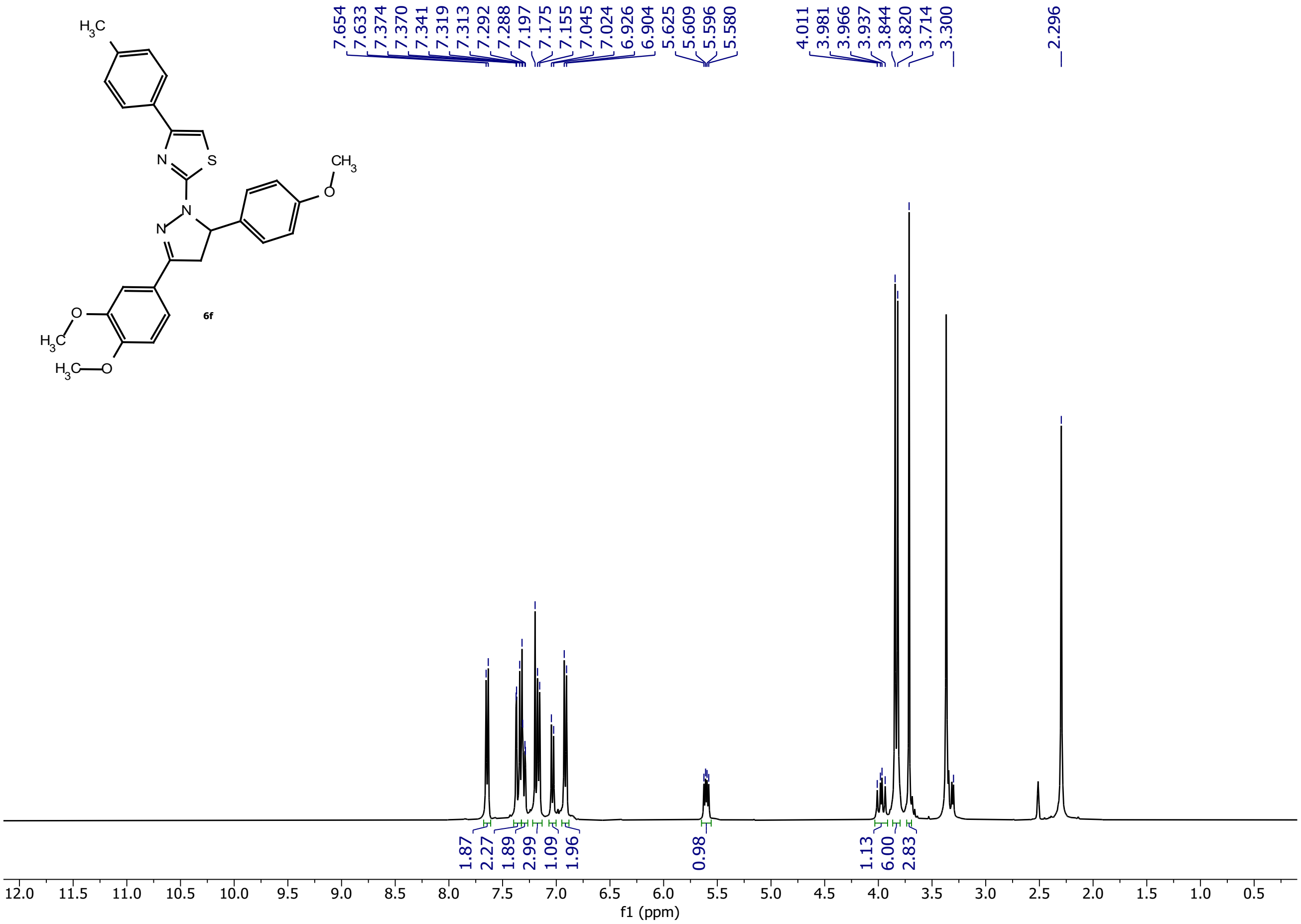

Figure S38: ^{1}H NMR of compound 6f

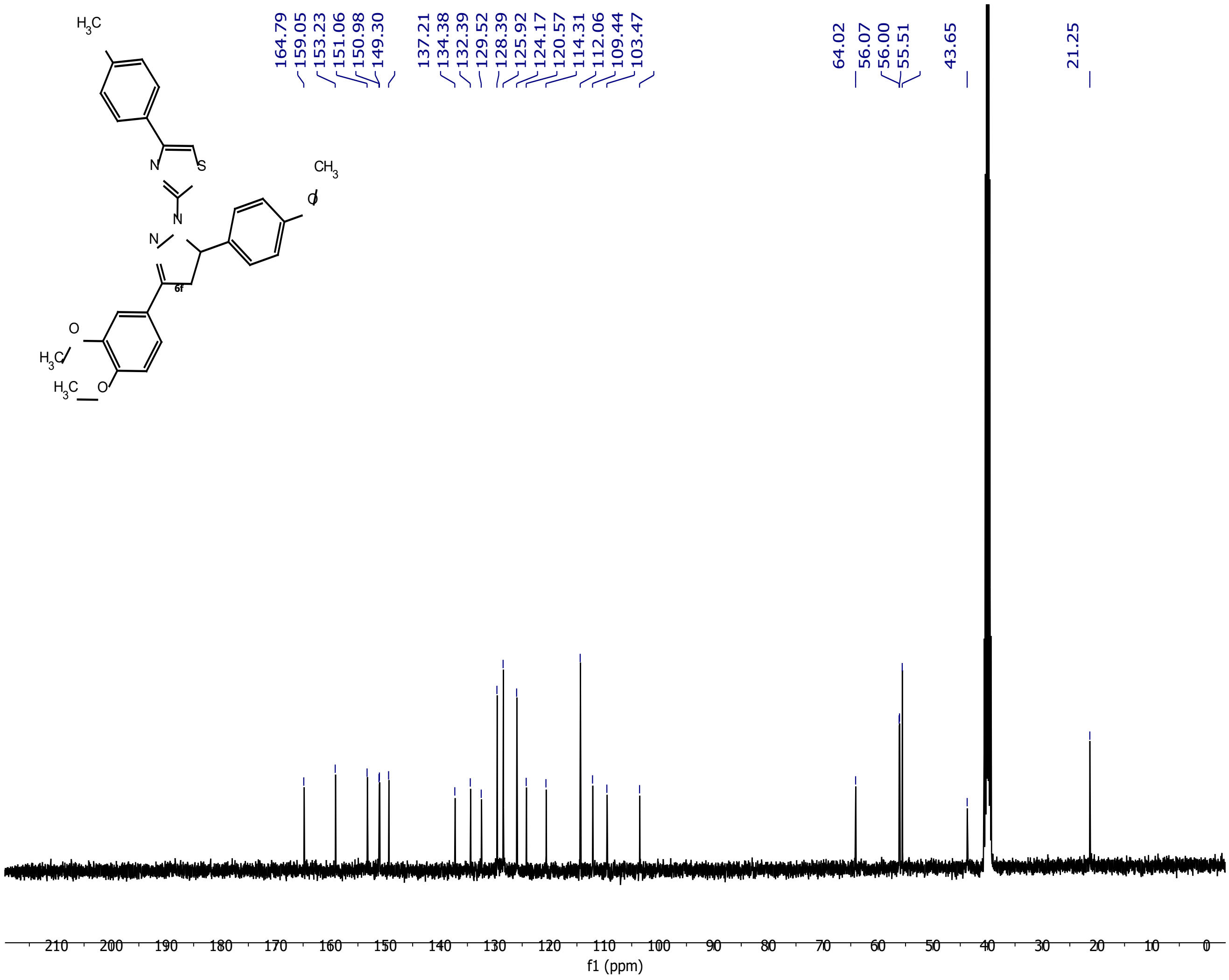

Figure S39: ^{13}C NMR of compound 6f

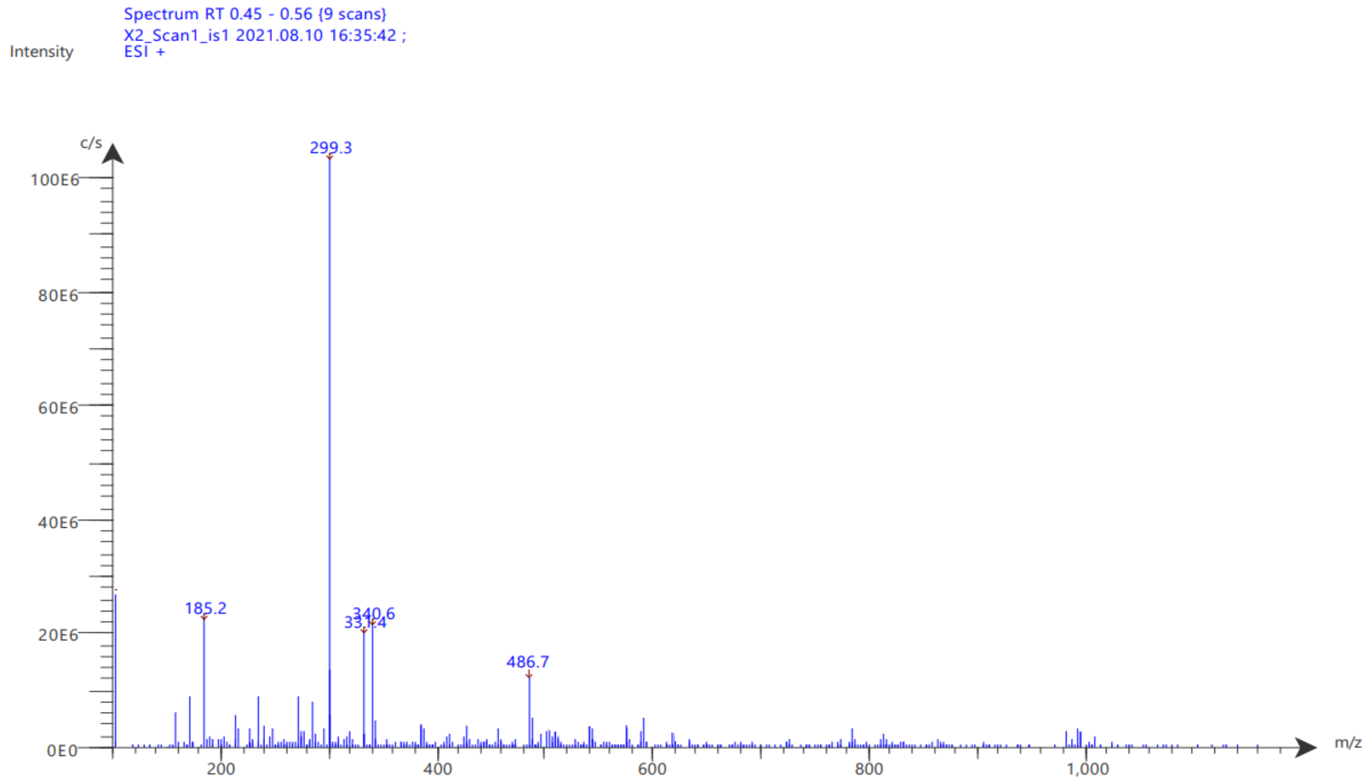

Figure S40: Mass of compound 6f

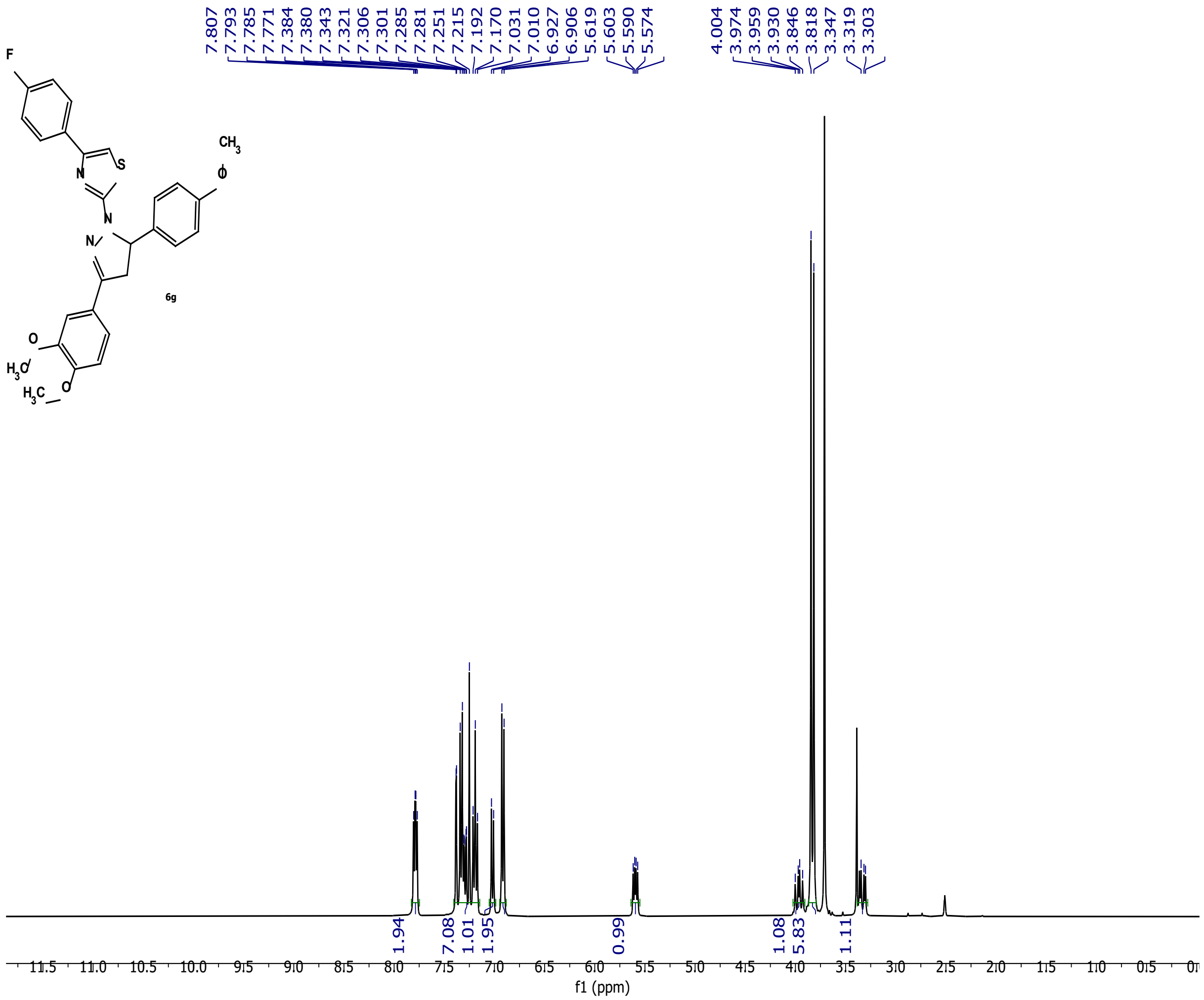

Figure S41: ^{1}H NMR of compound 6g

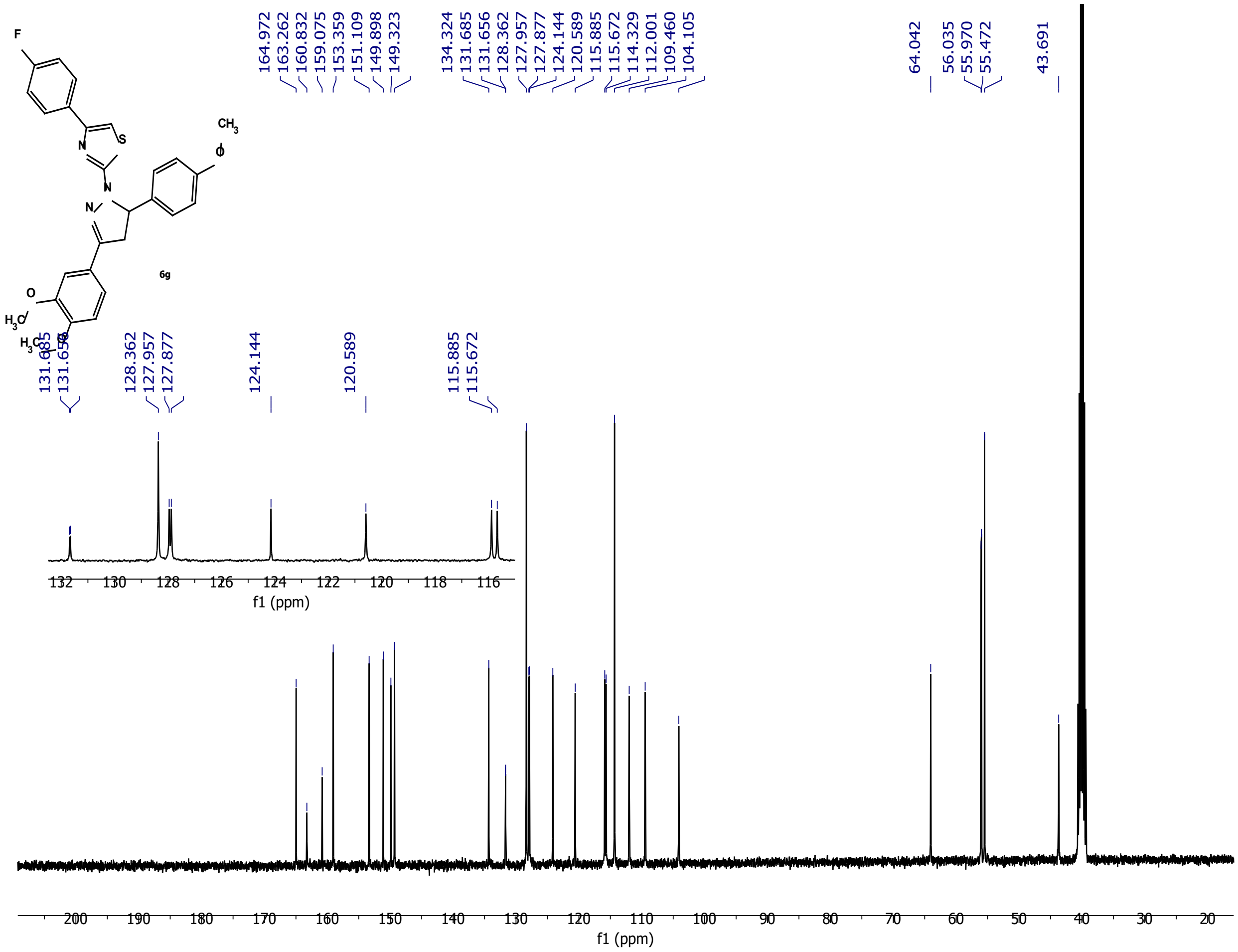

Figure S42: ^{13}CNMR of compound 6g

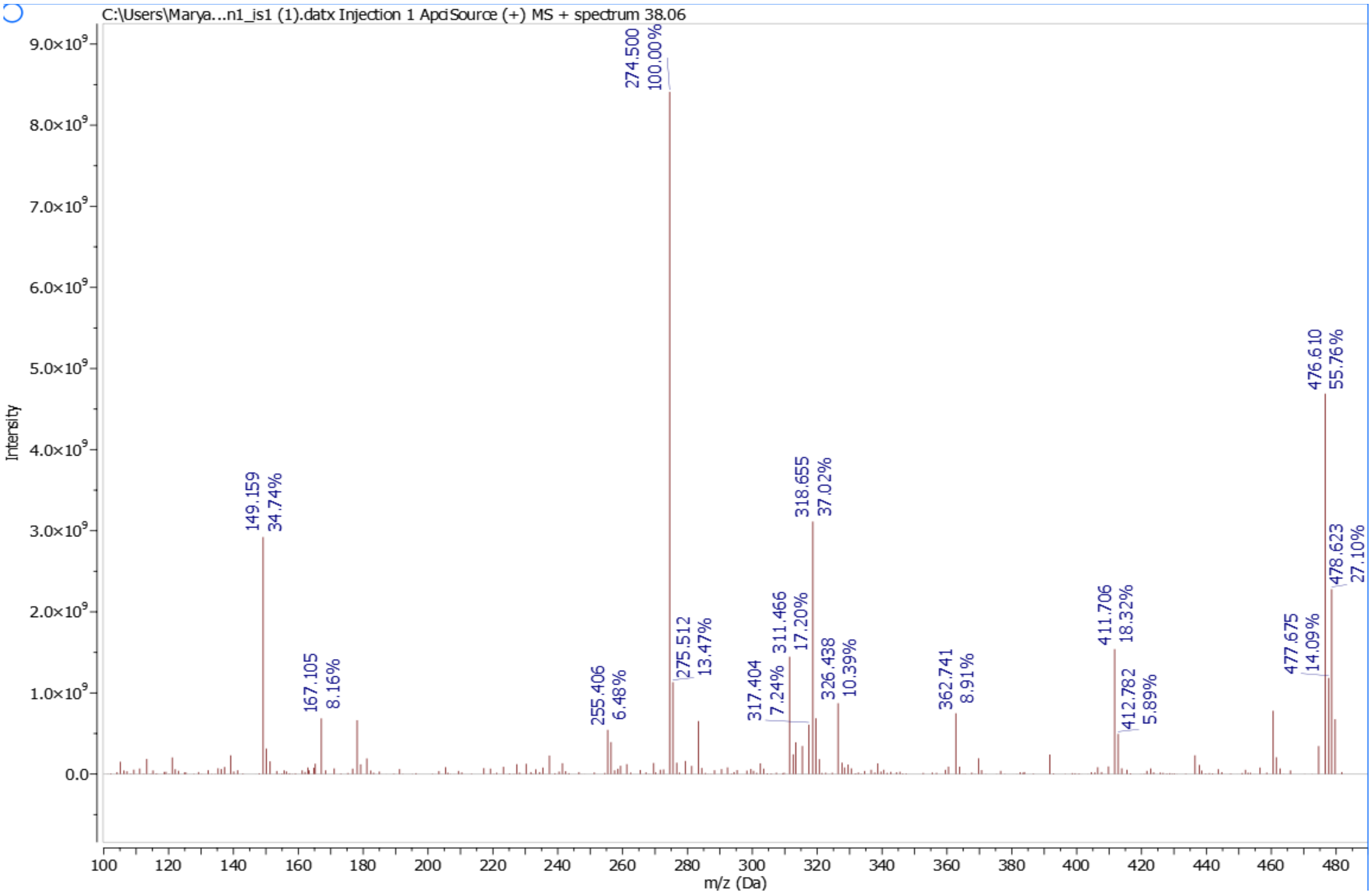

Figure S43: Mass of compound 6g

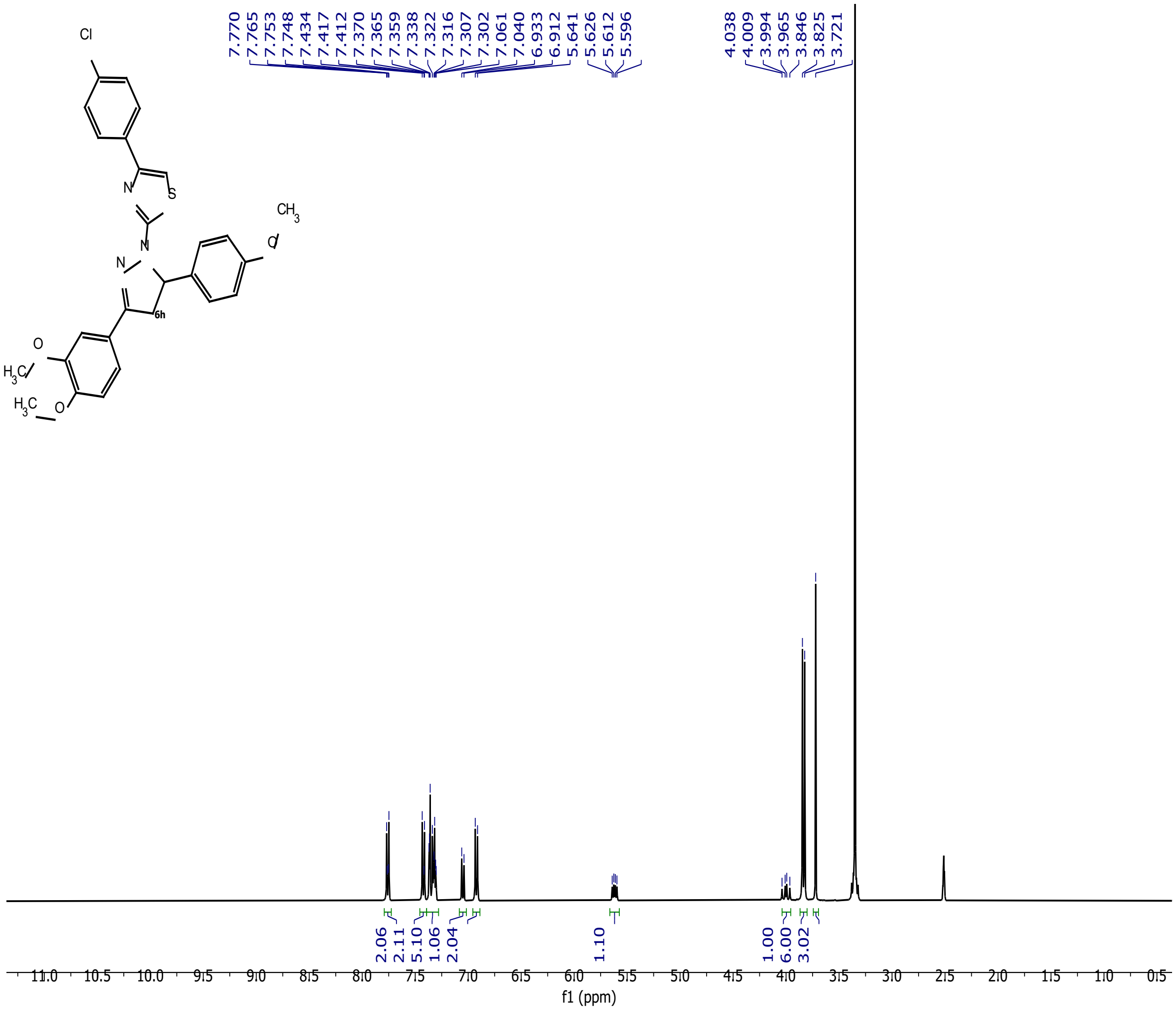

Figure S44: ^{1}H NMR of compound 6h

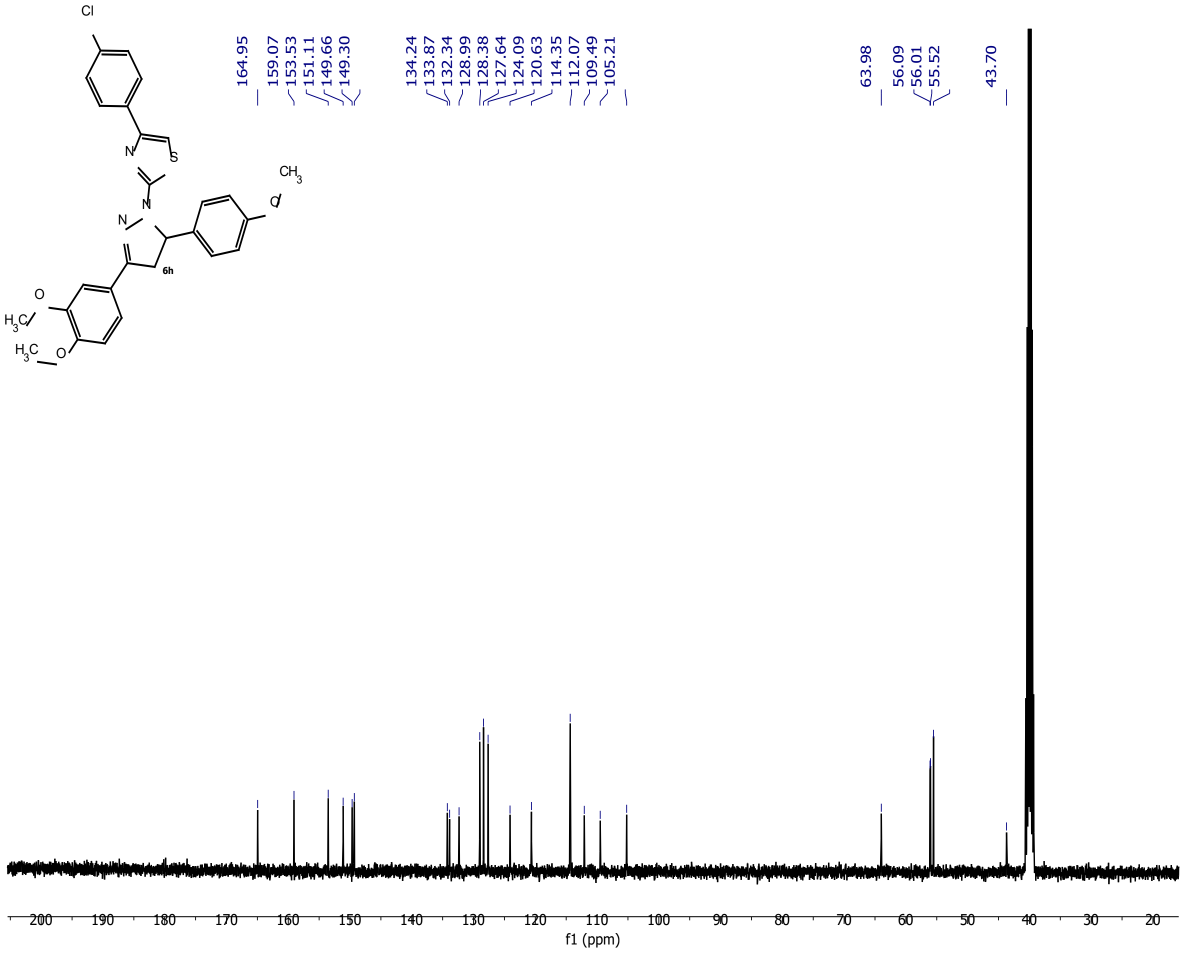

Figure S45: ^{13}C NMR of compound 6h

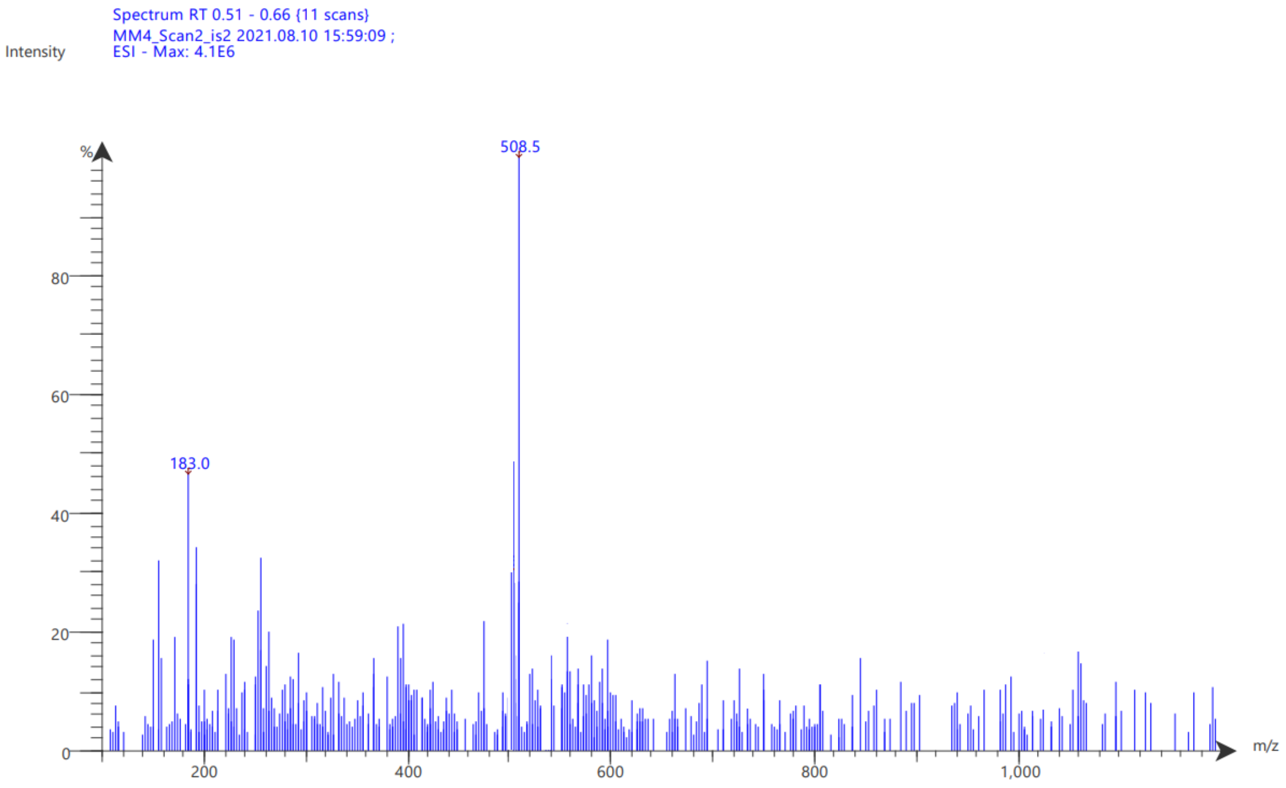

Figure S46: Mass of compound 6h

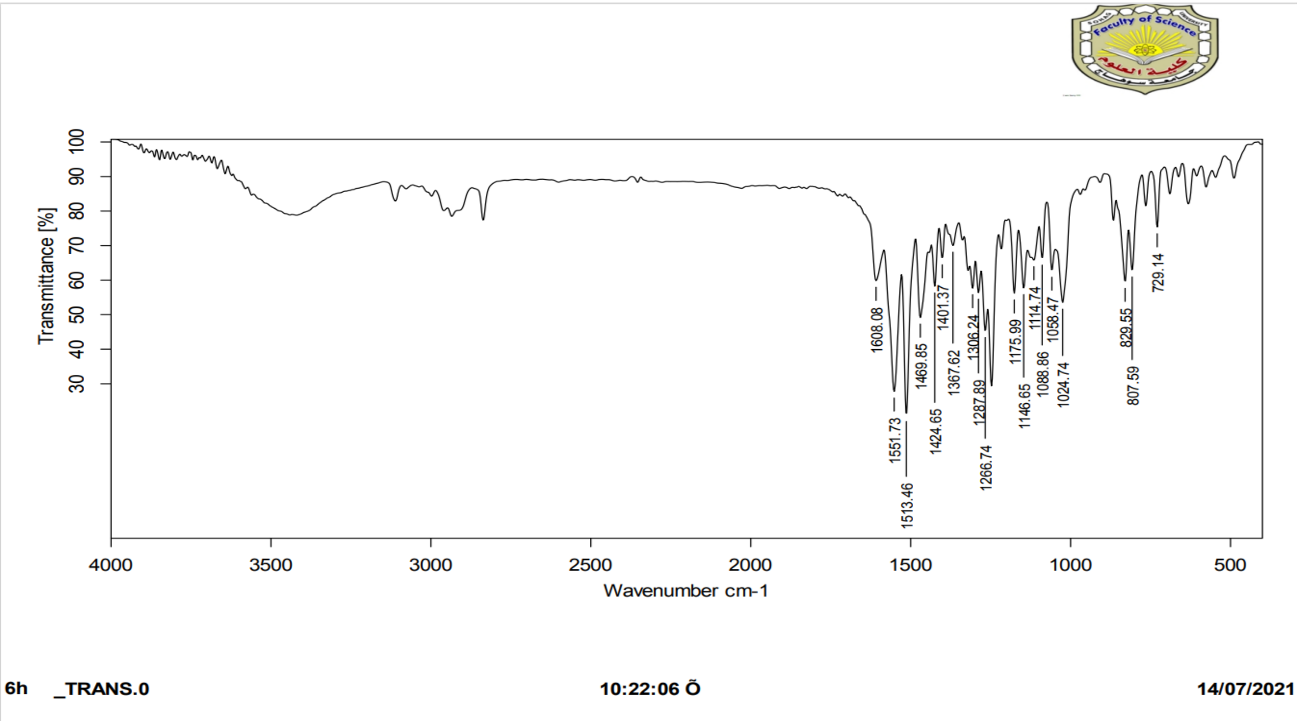

Figure S47: IR of compound 6h

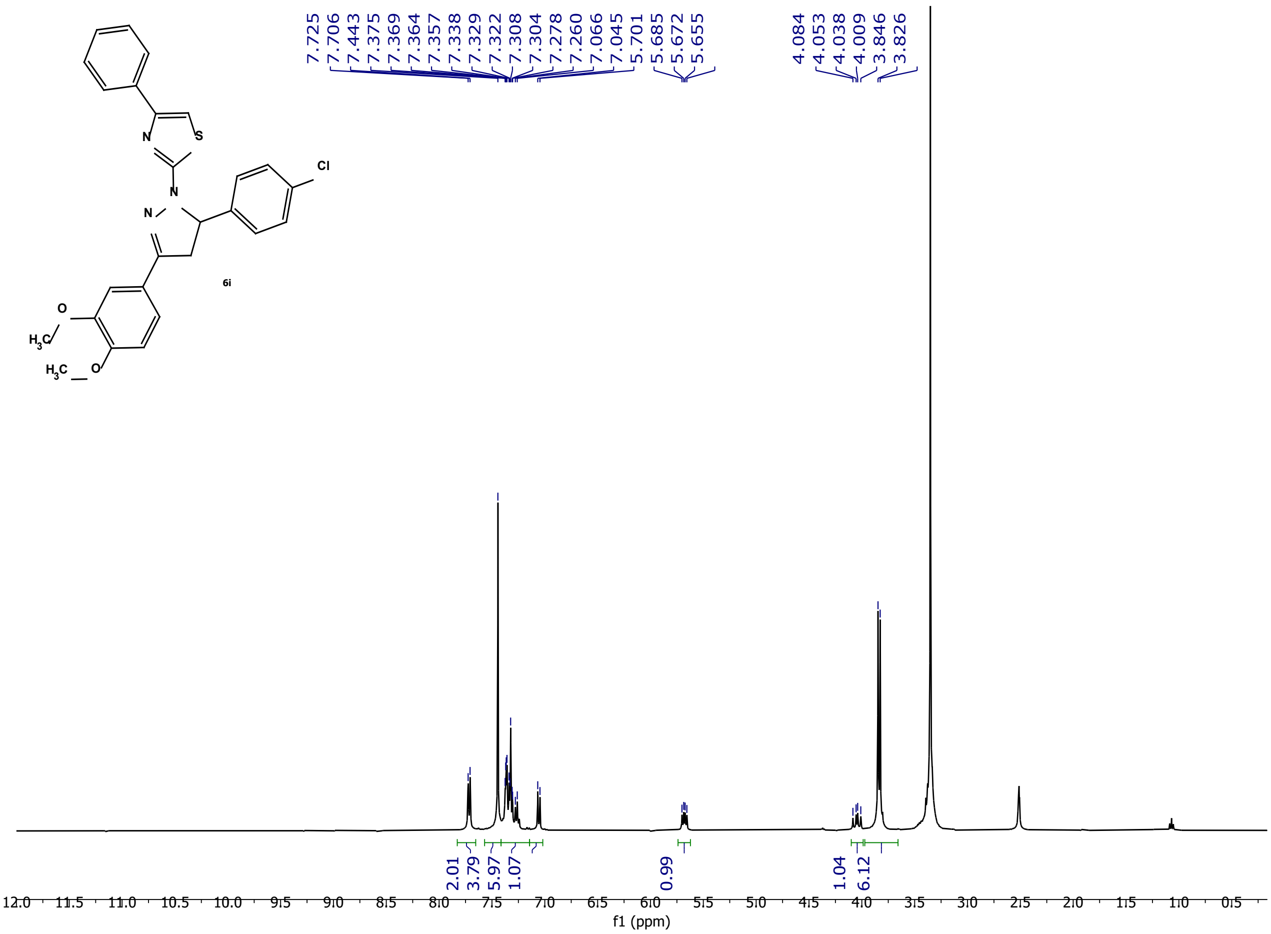

Figure S48: ^{1}H NMR of compound 6i

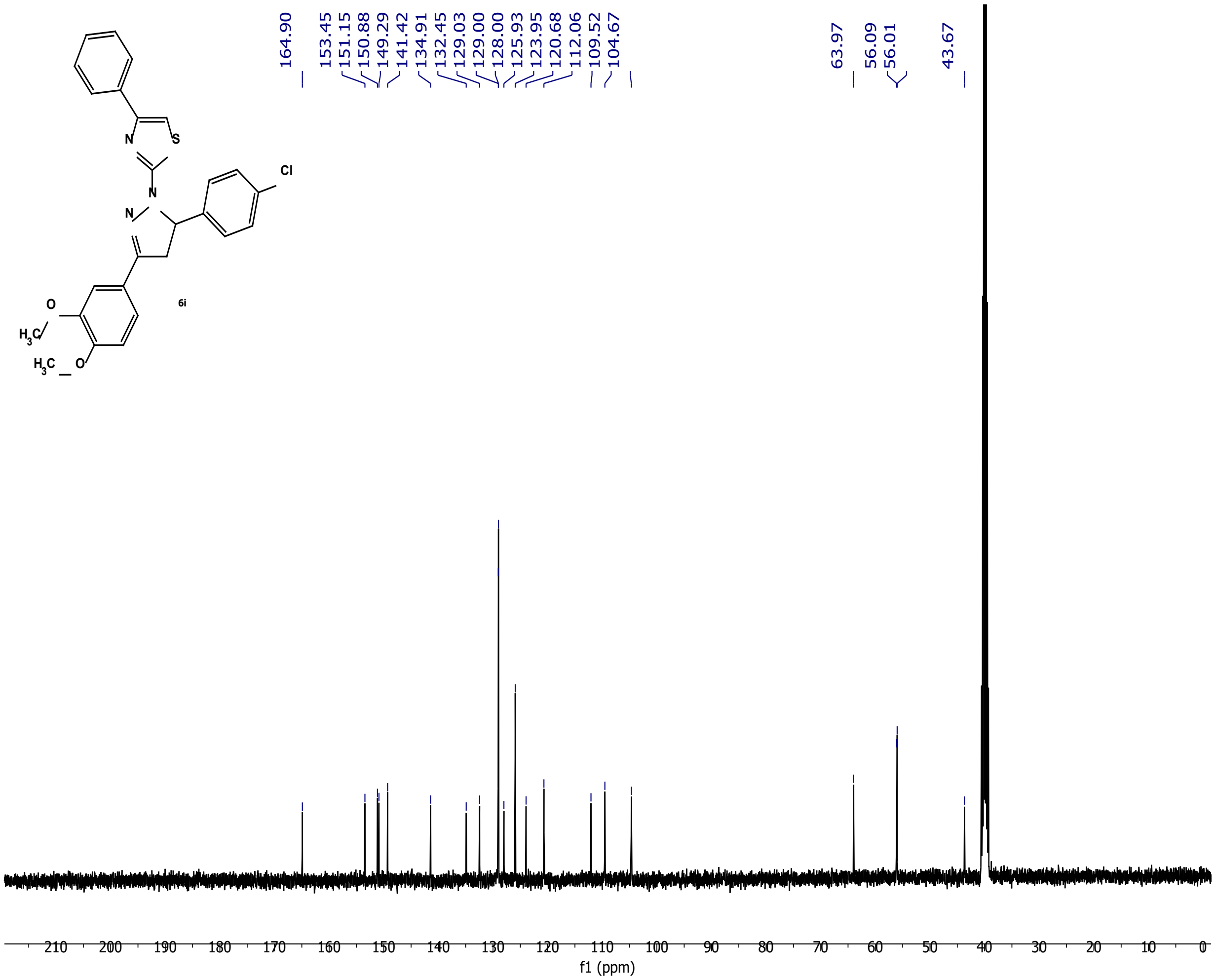

Figure S49: ^{13}C NMR of compound 6i

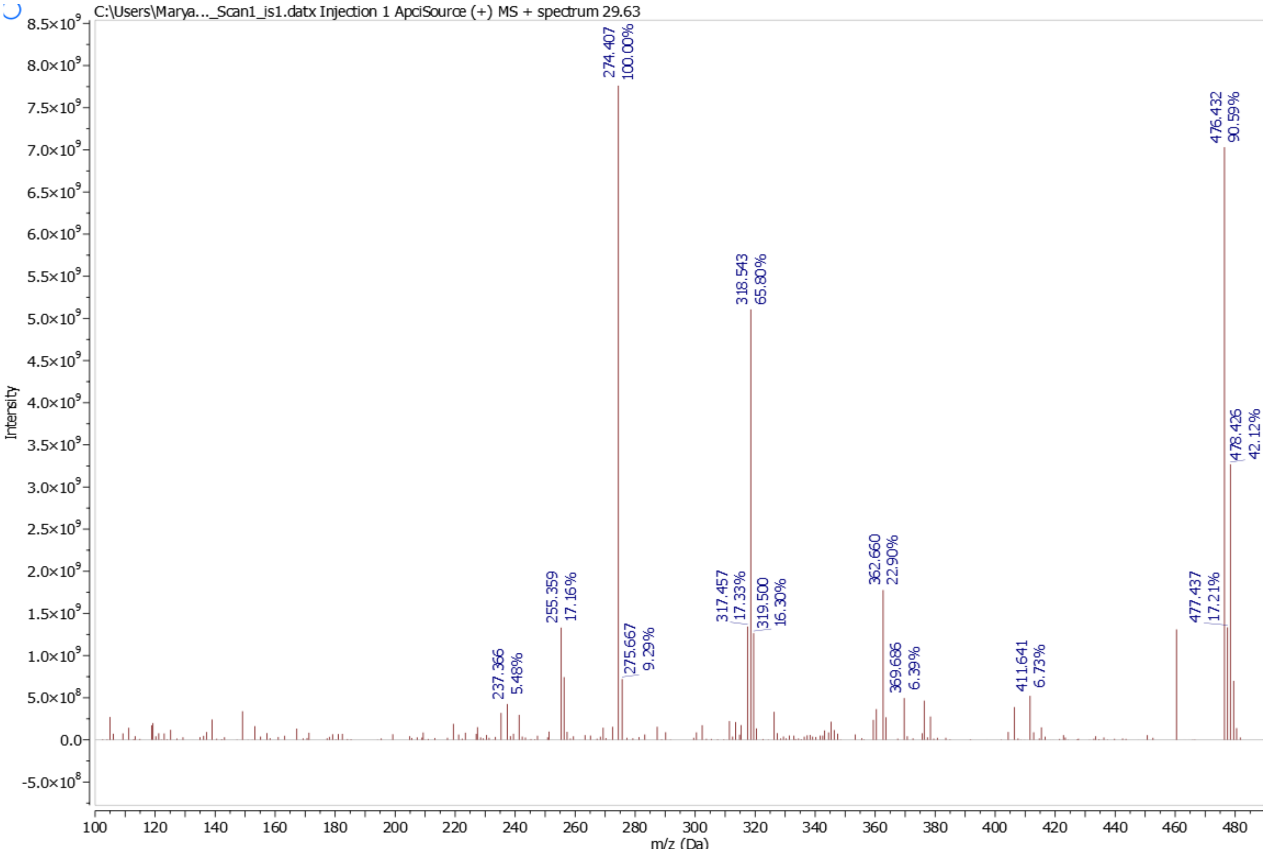

Figure S50: Mass of compound 6i

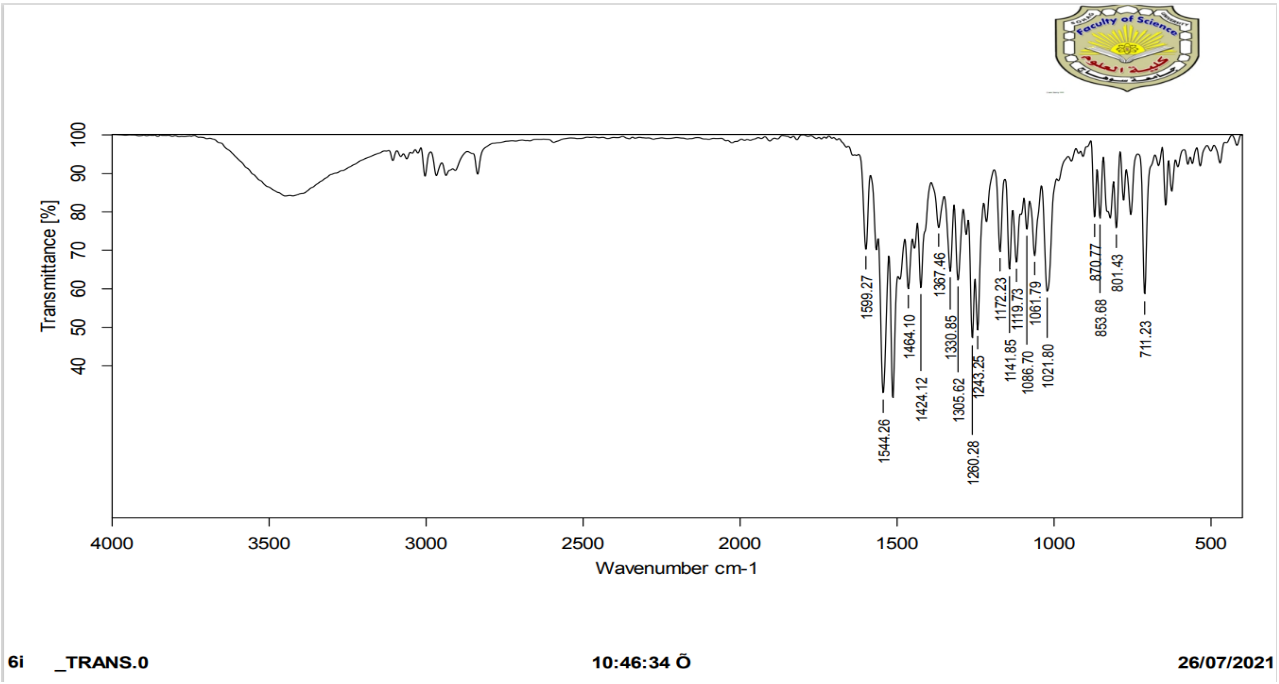

Figure S51: IR of compound 6i

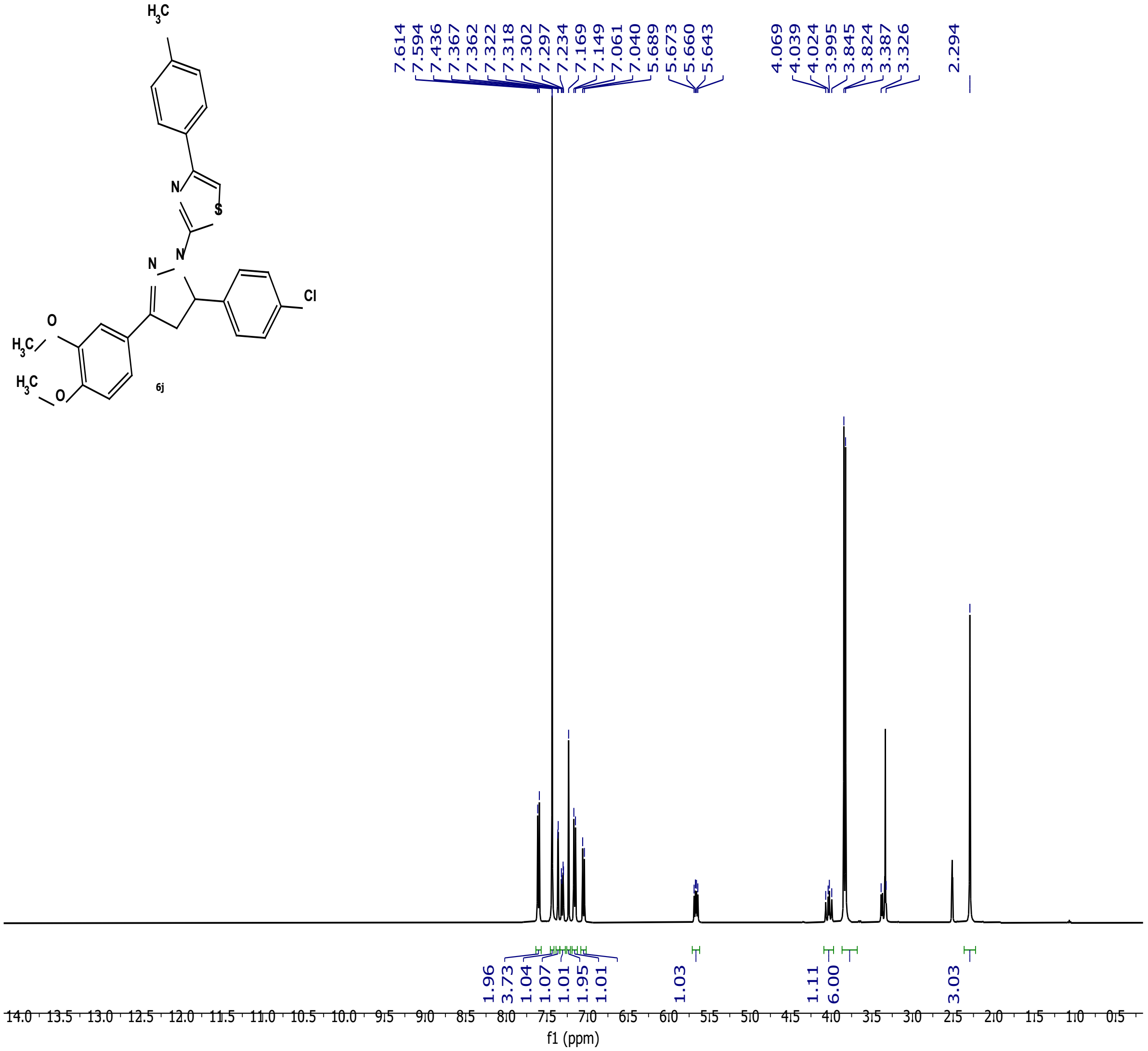

Figure S52: ^{1}H NMR of compound 6j

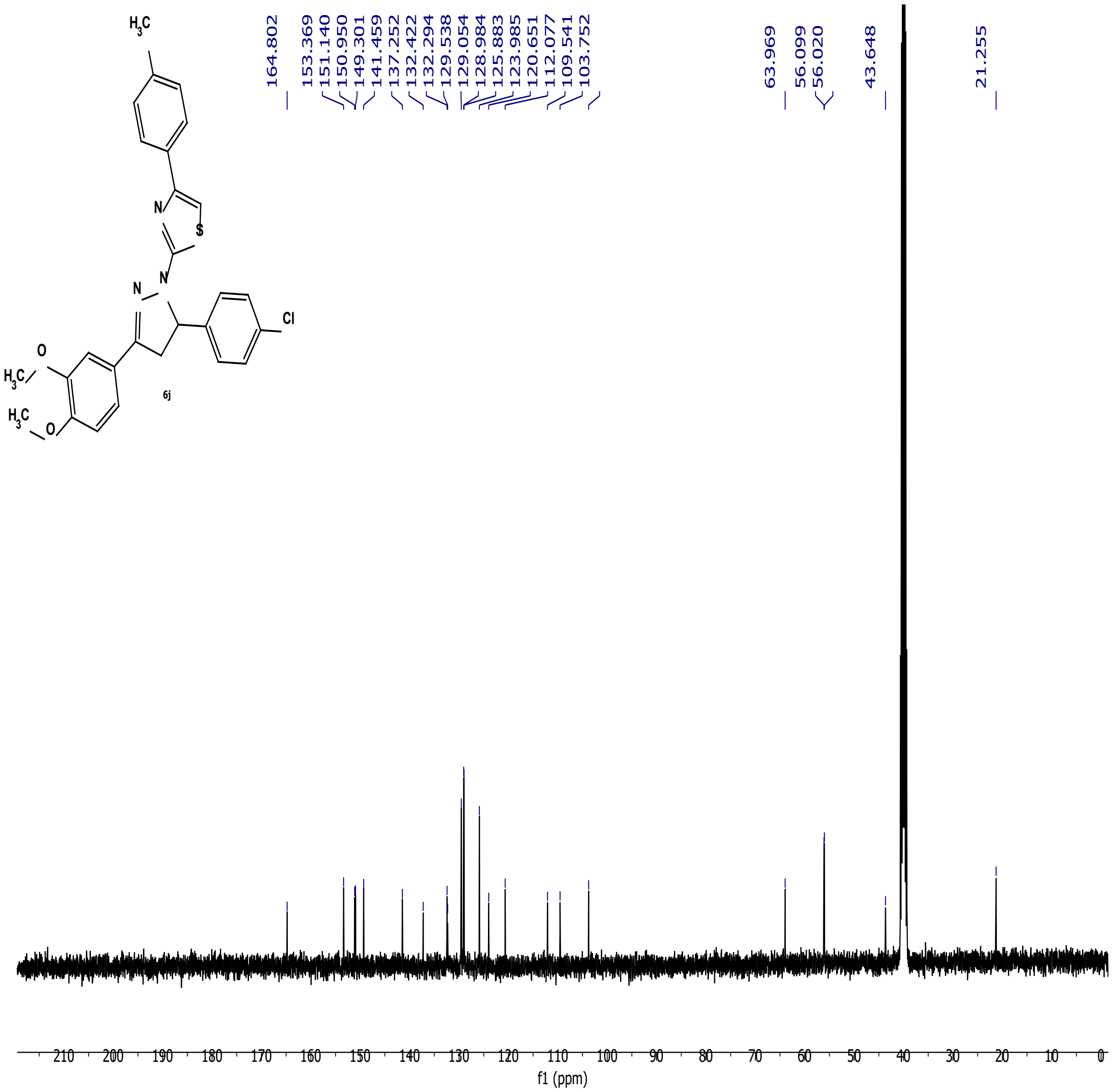

Figure S53: ^{13}C NMR of compound 6j

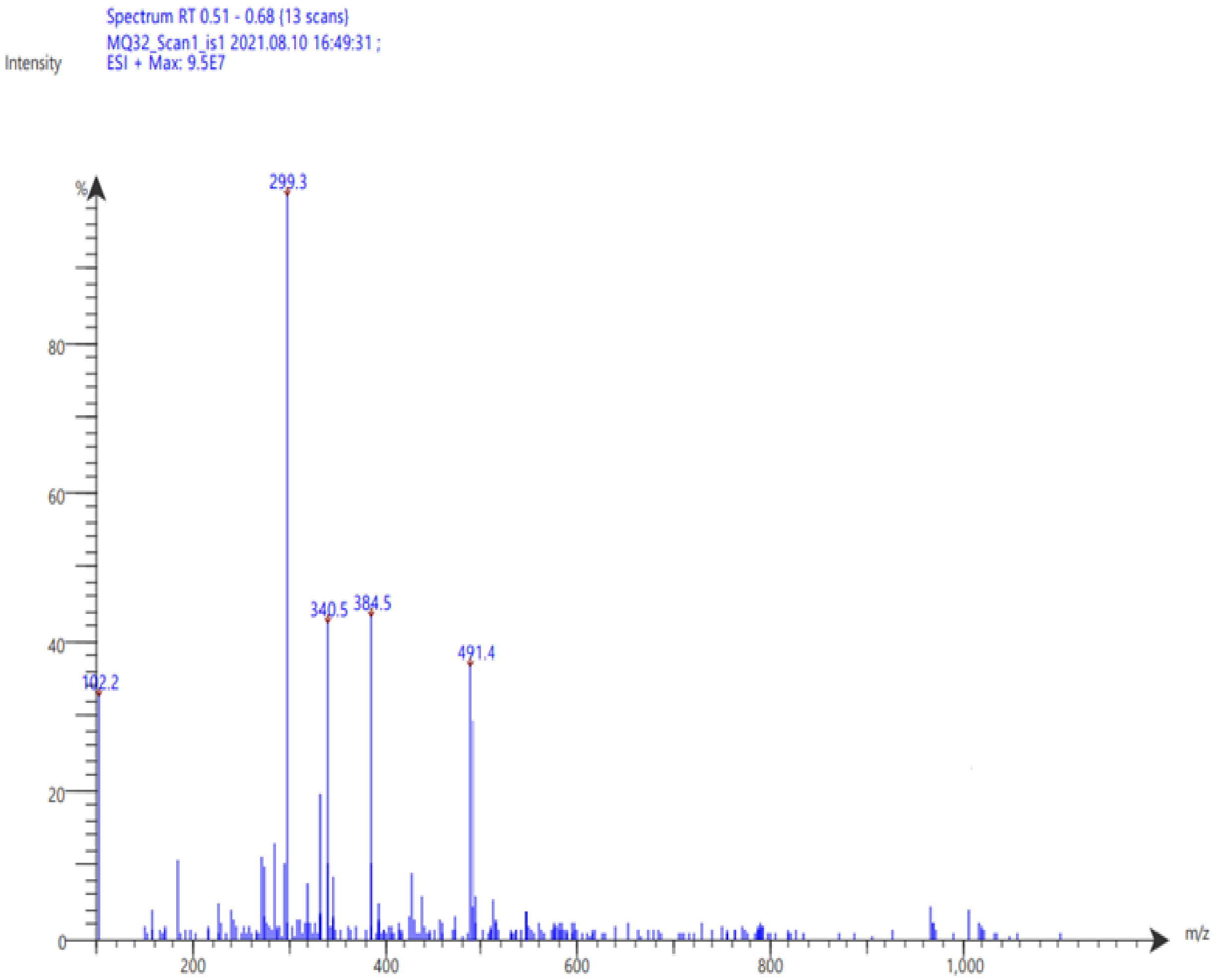

Figure S54: Mass of compound 6j

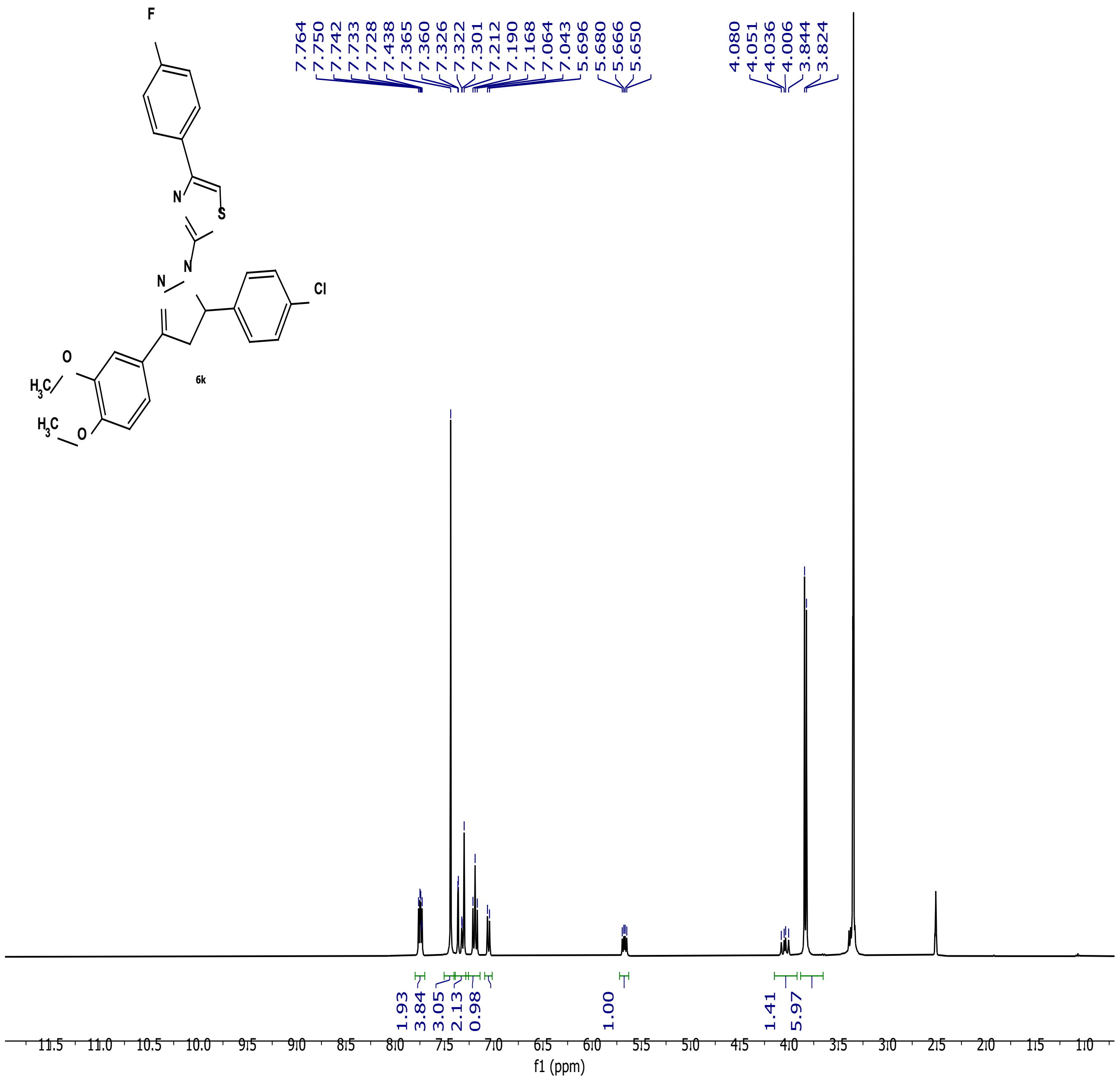

Figure S55: ¹H NMR of compound 6k

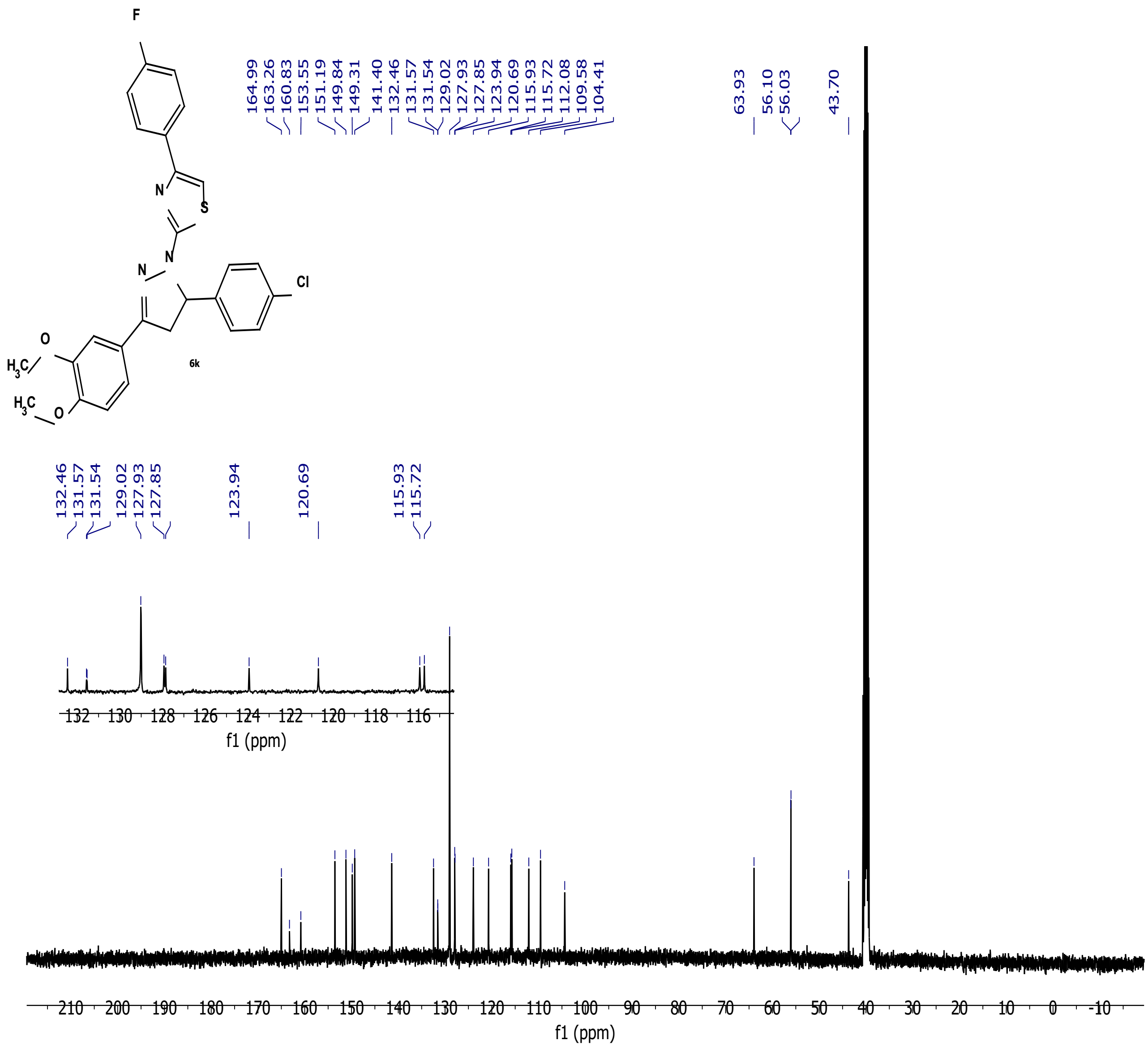

Figure S56: ^{13}C NMR of compound 6k

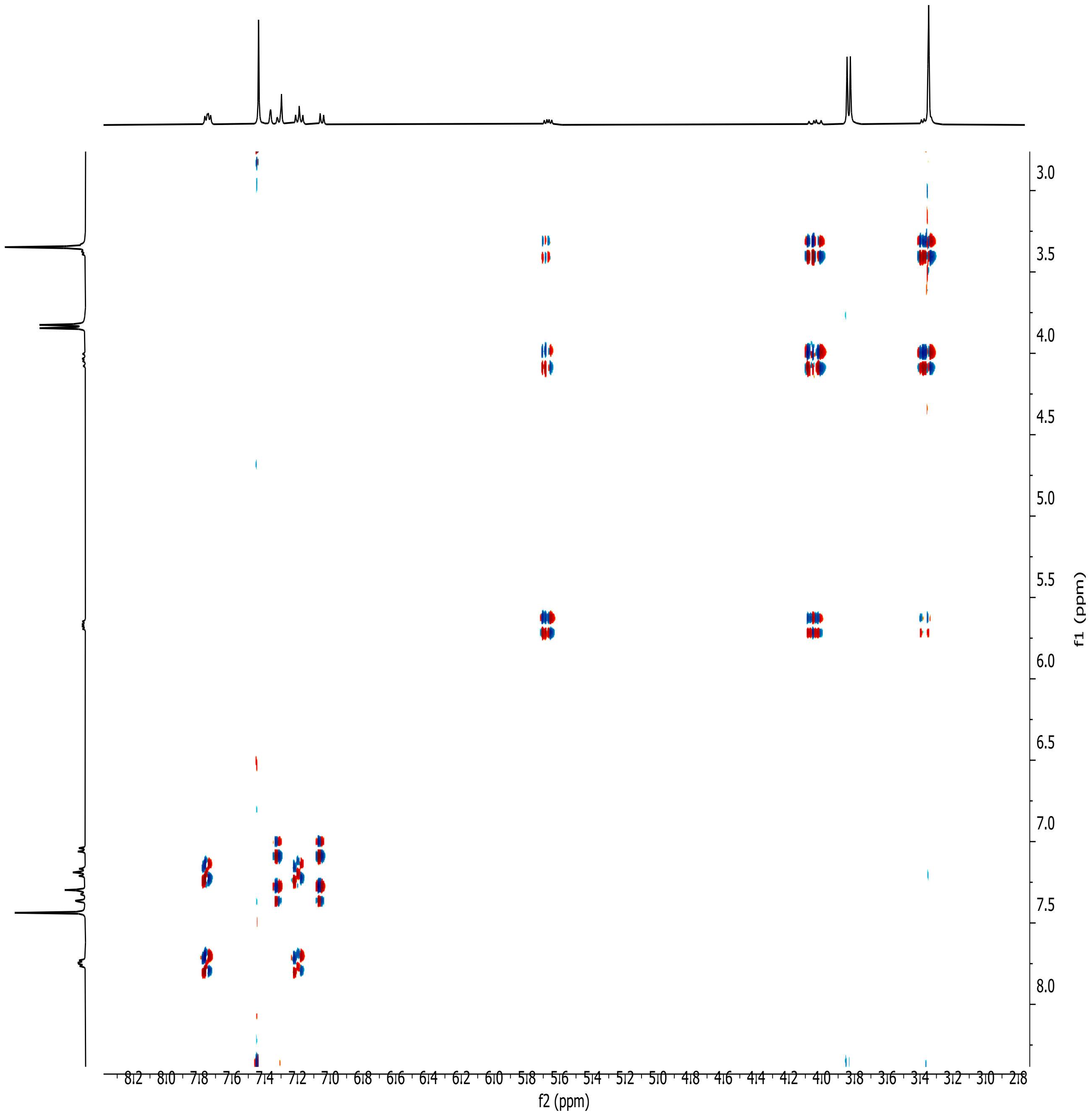

Figure S57: ^{1}H-^{1}H Cosy of compound 6k

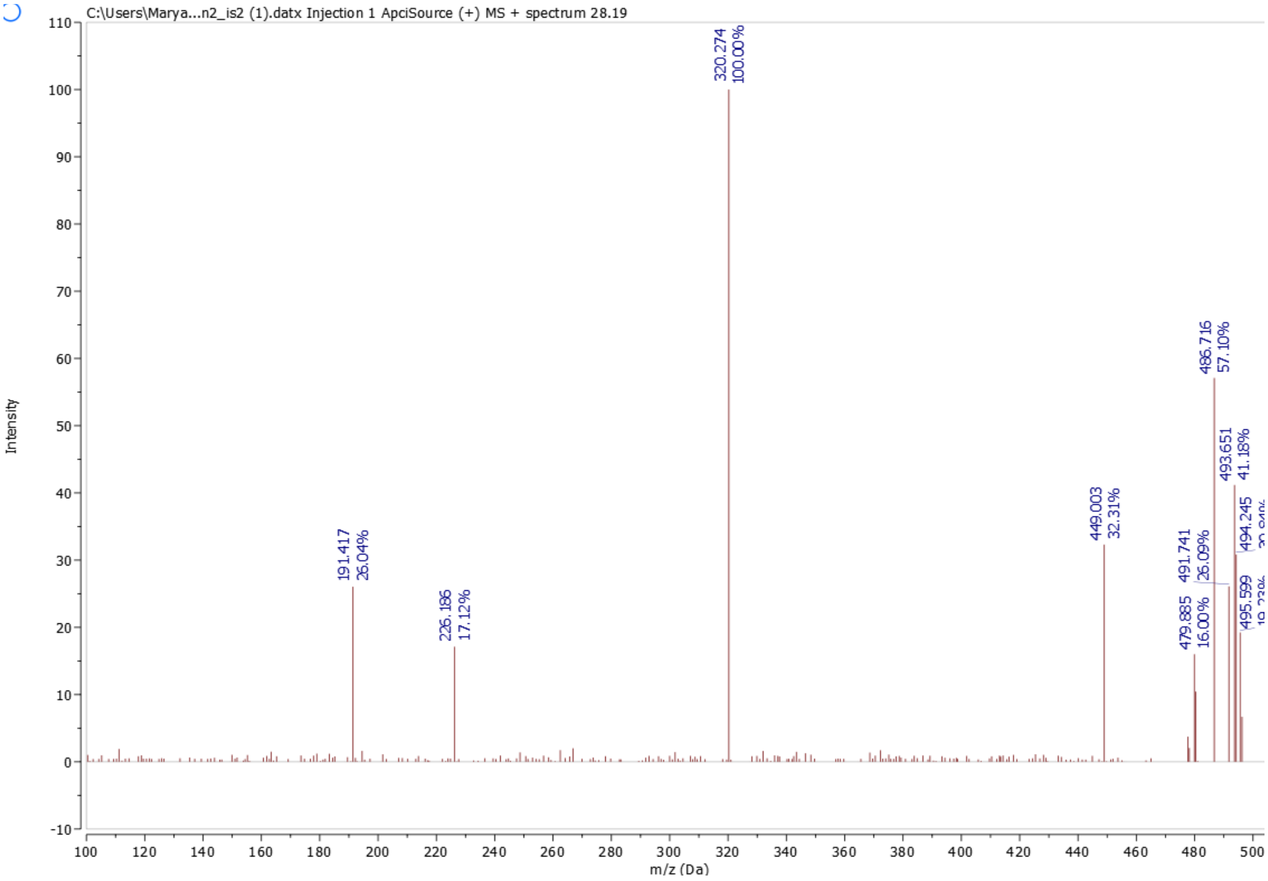

Figure S58: Mass of compound 6K

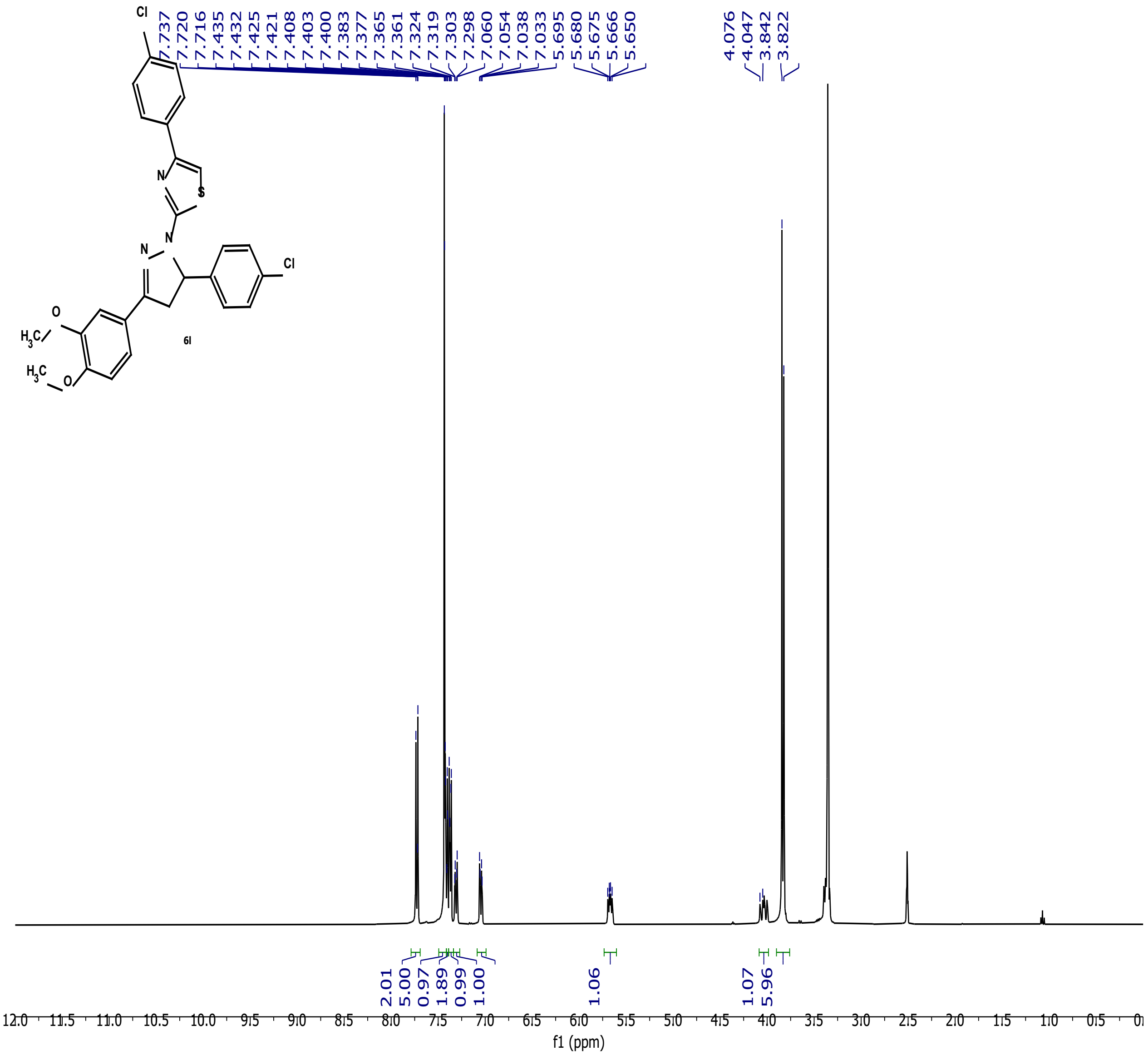

Figure S59: ^{1}H NMR of compound 6l

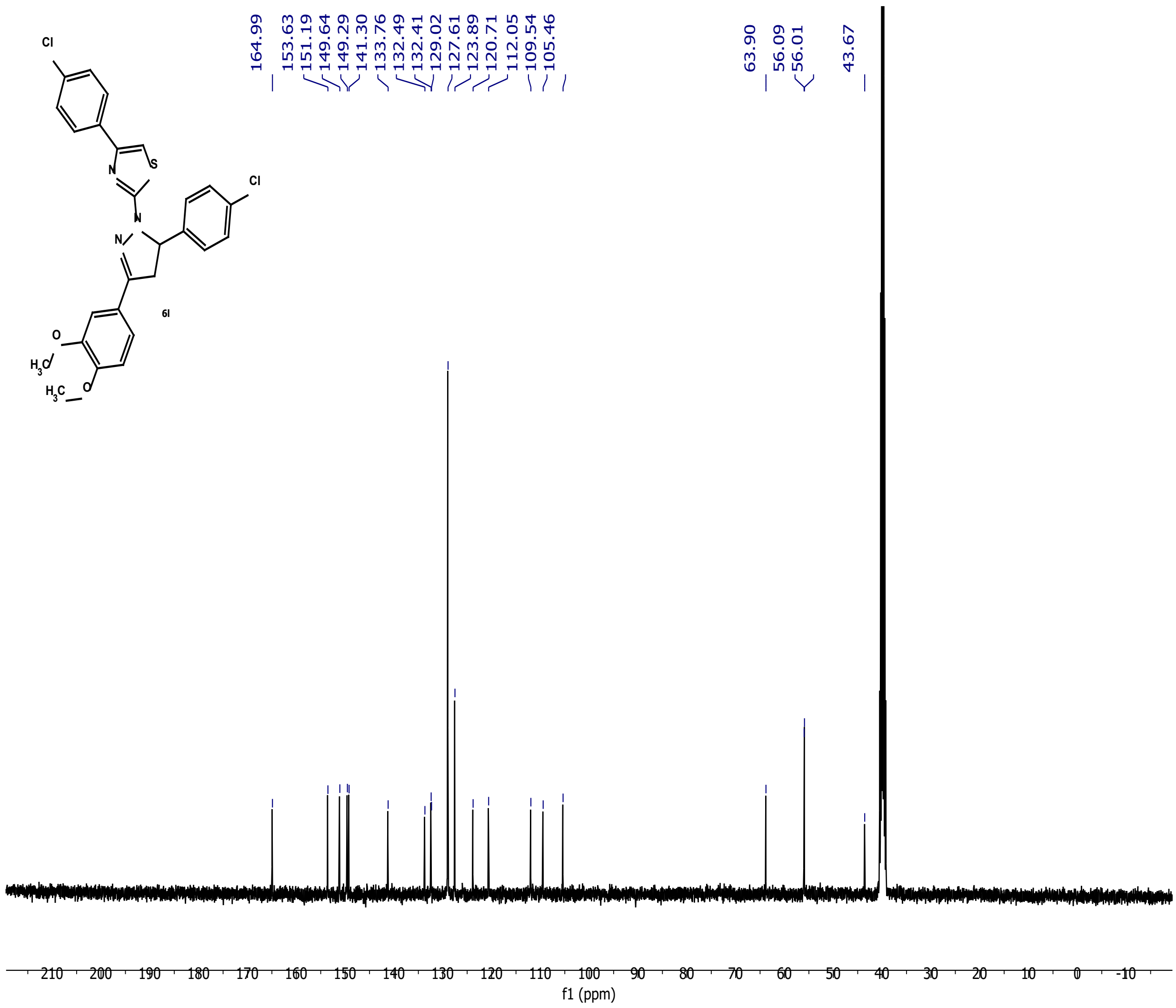

Figure S60: ^{13}C NMR of compound 6l

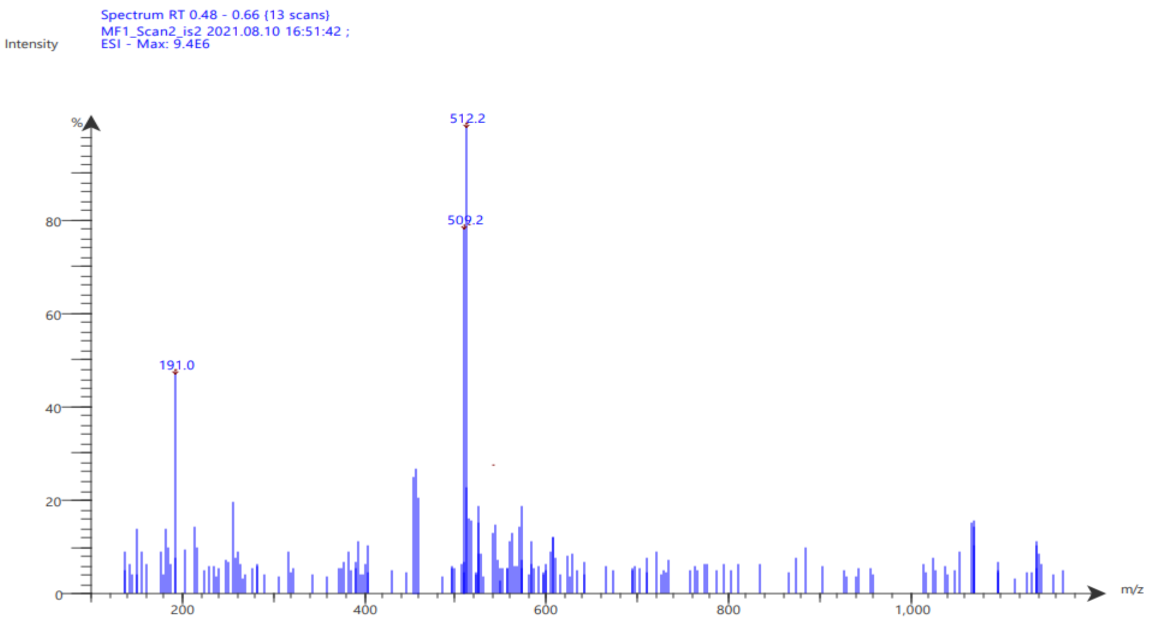

Figure S61: Mass of compound 6l

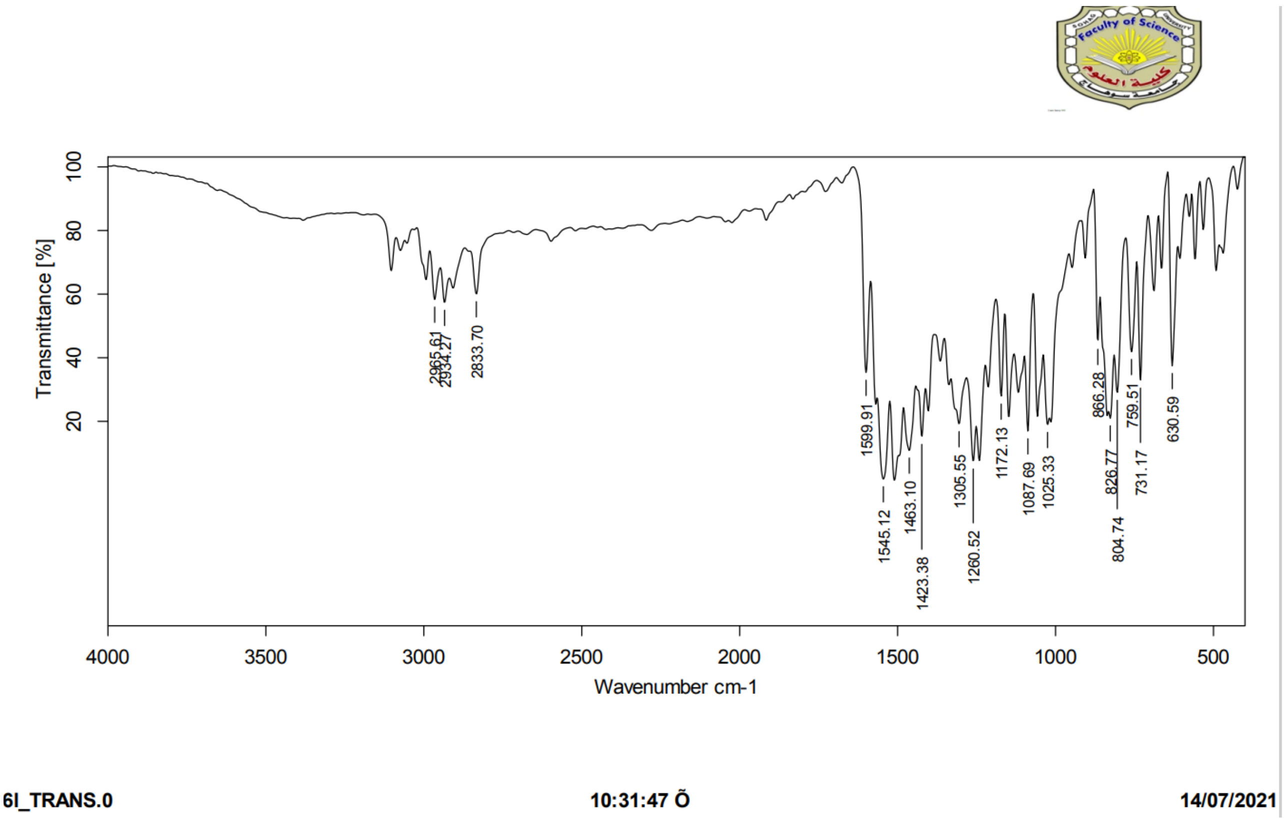

Figure S62: IR of compound 6l

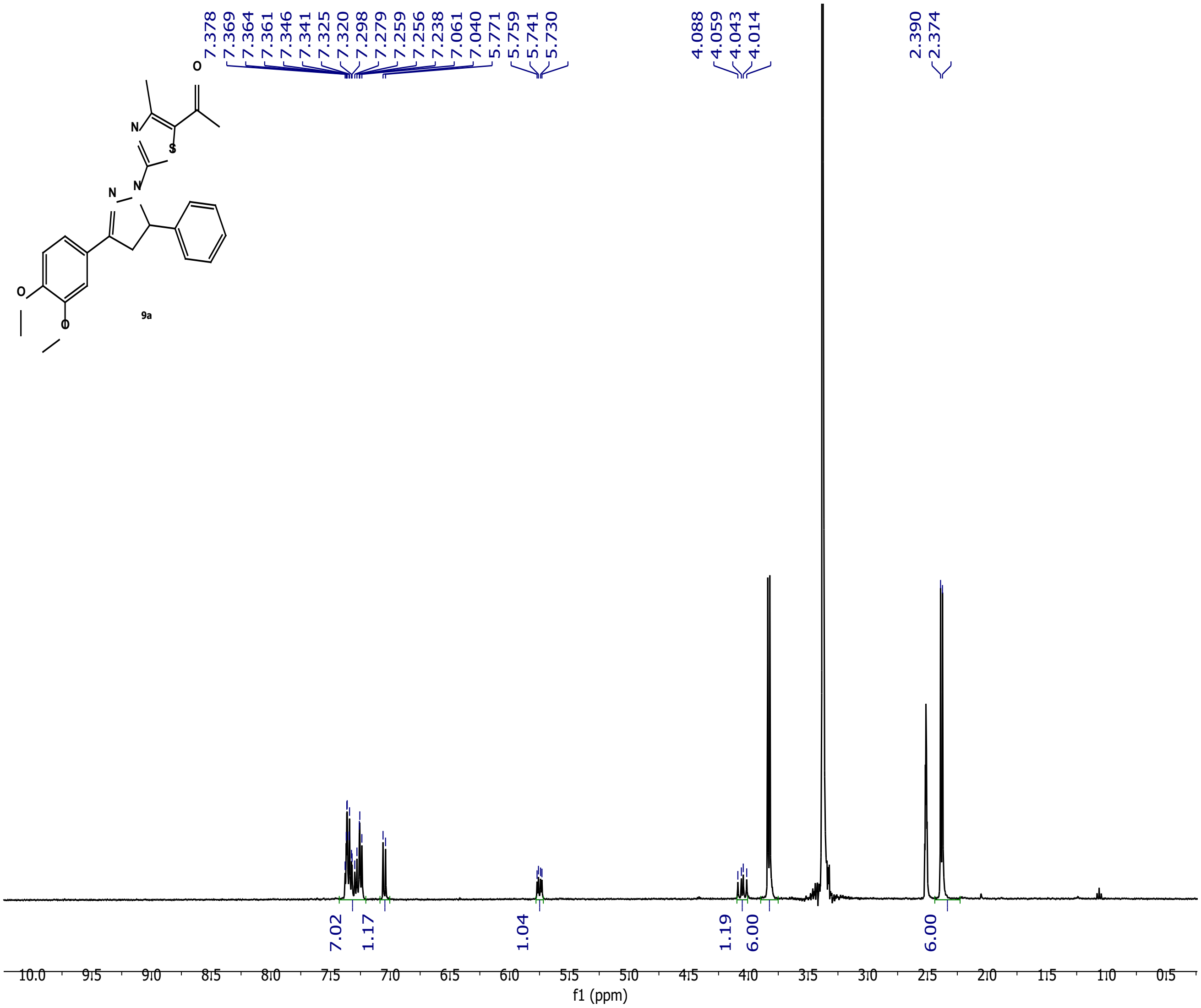

Figure S63: ^{1}H NMR of compound 9a

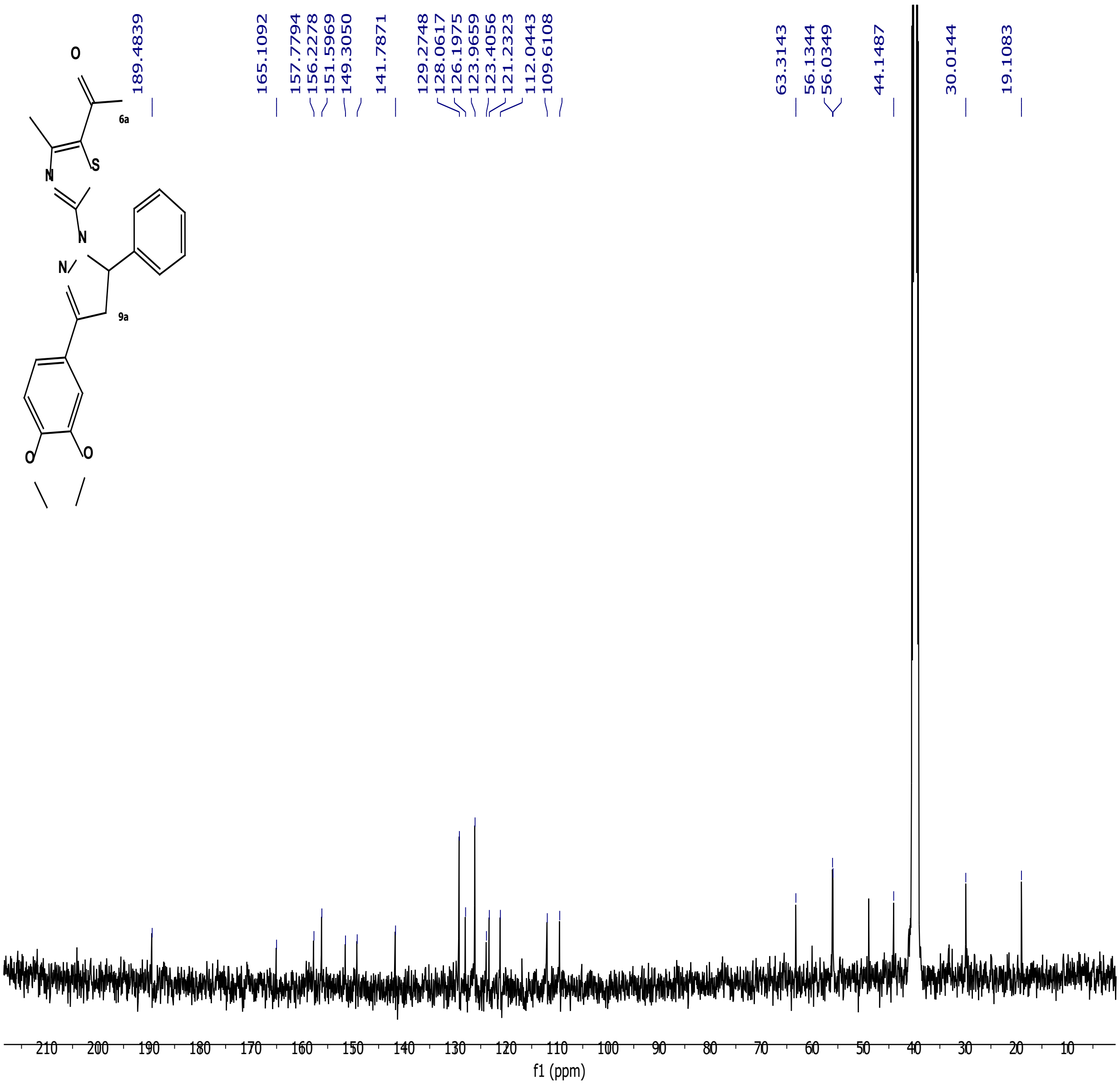

Figure S64: ^{13}C NMR of compound 9a

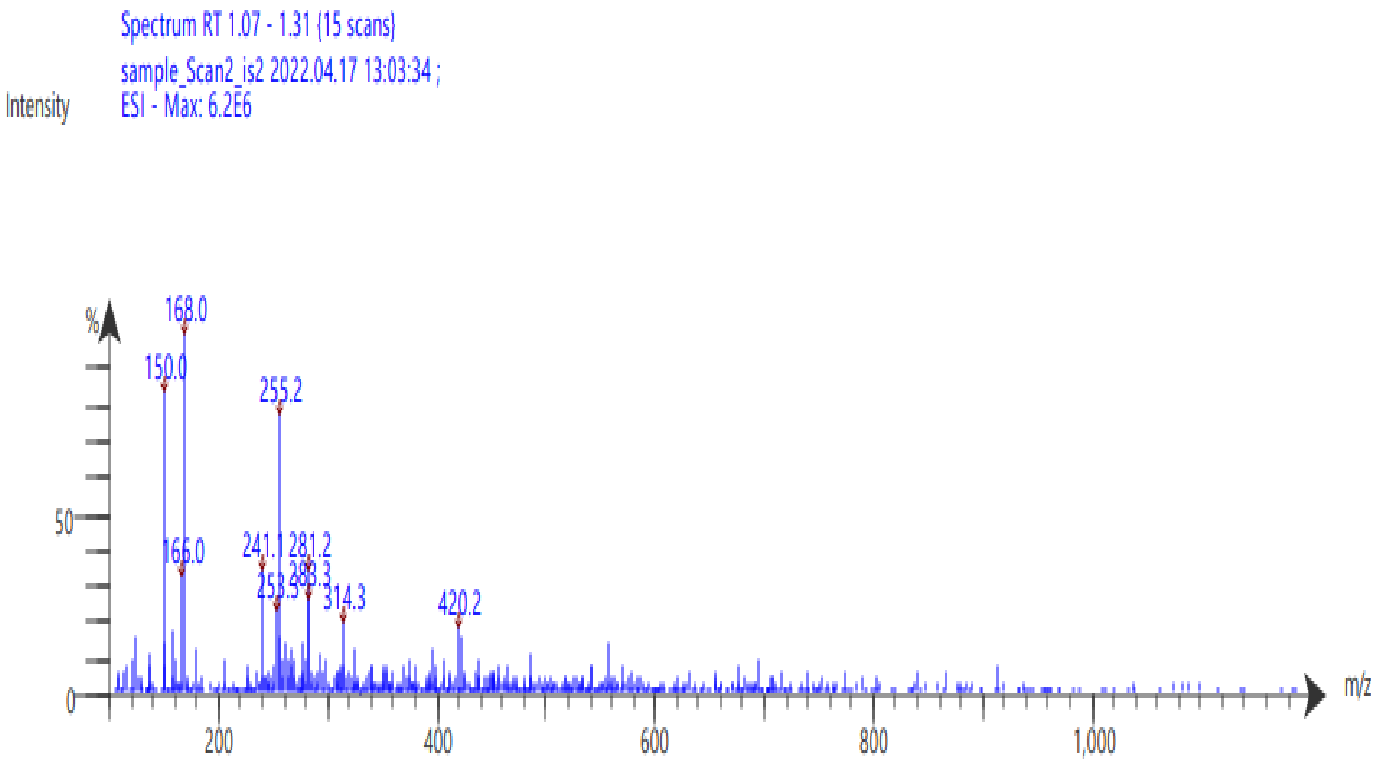

Figure S65: Mass of compound 9a

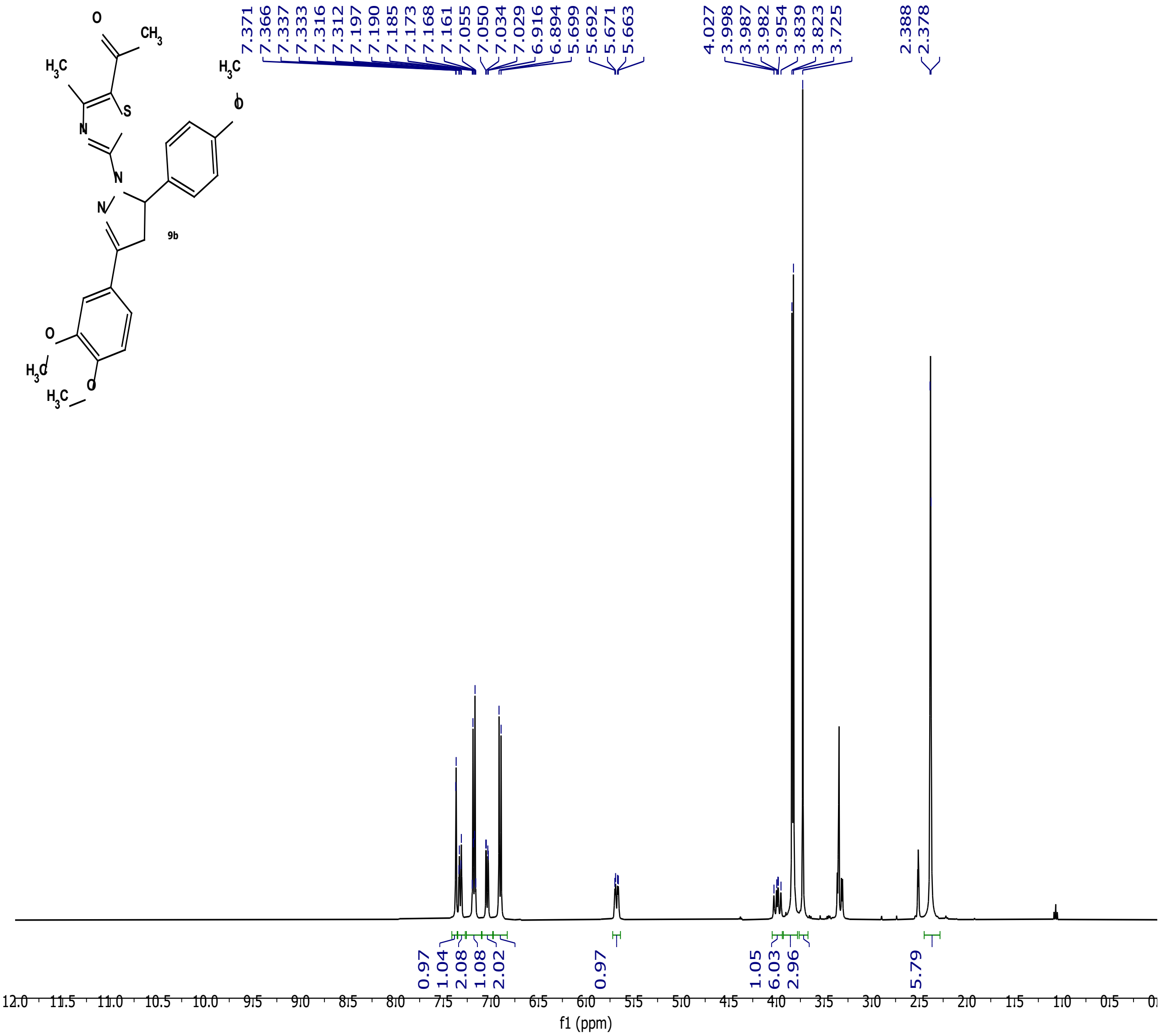

Figure S66: ¹H NMR of compound 9b

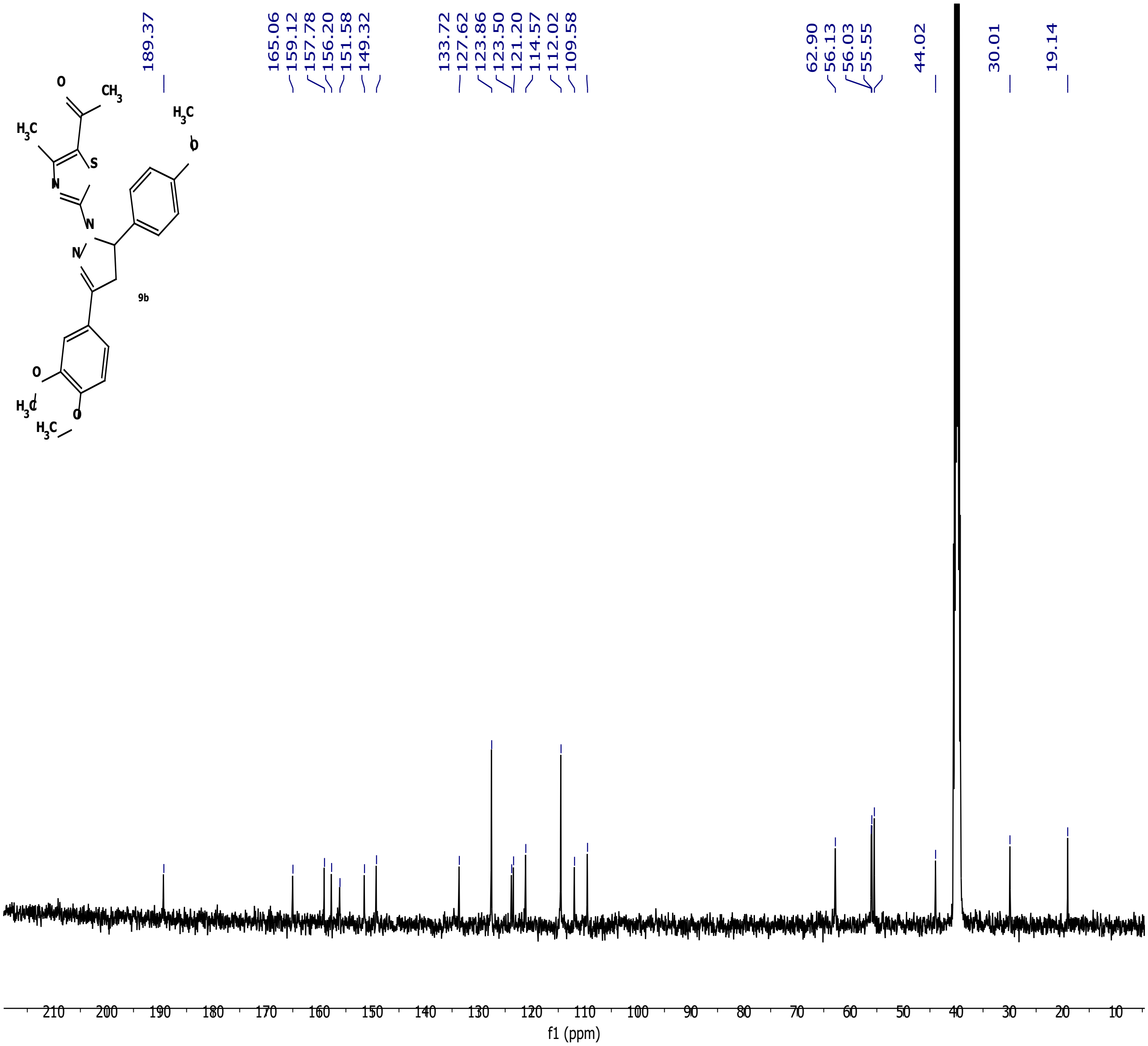

Figure S67: ^{13}C NMR of compound 9b

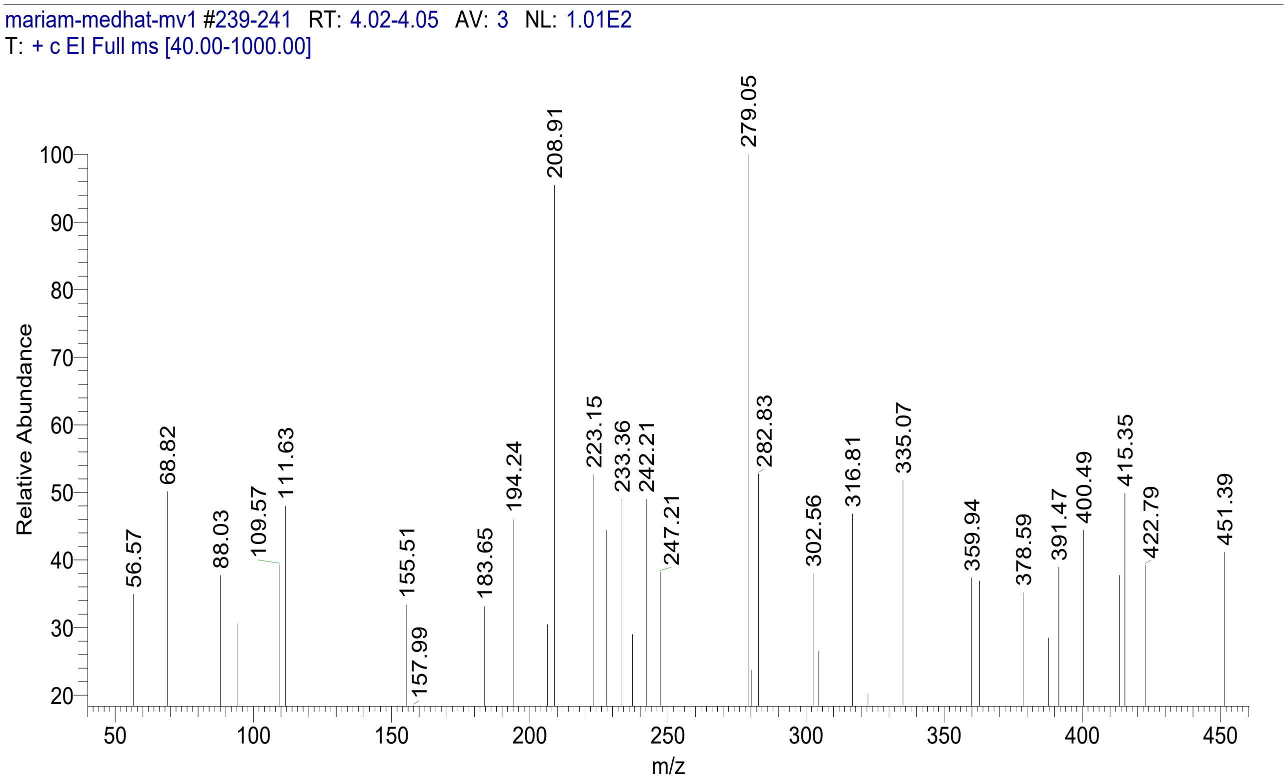

Figure S68: Mass of compound 9b

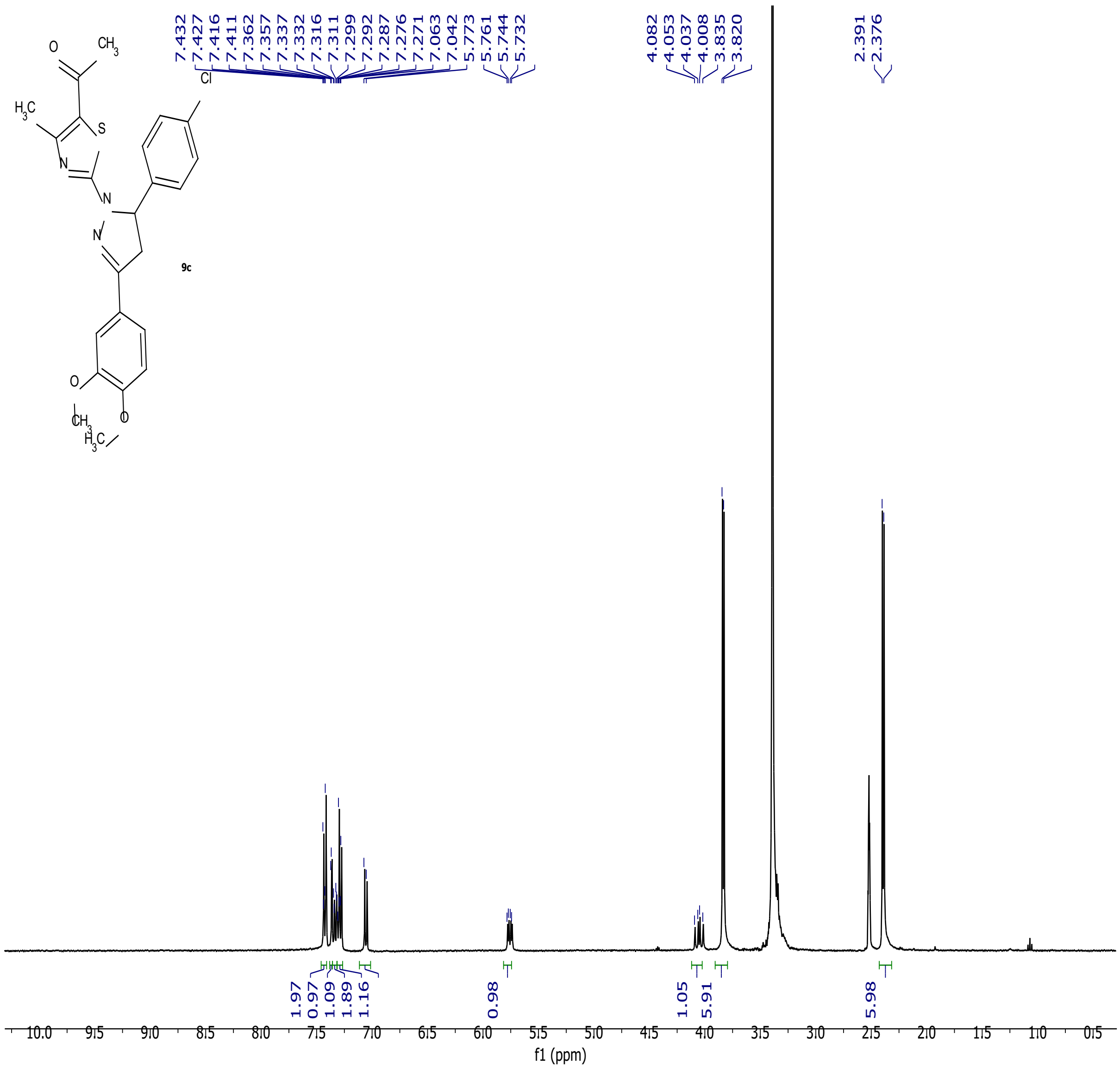

Figure S69: ^{1}H NMR of compound 9c

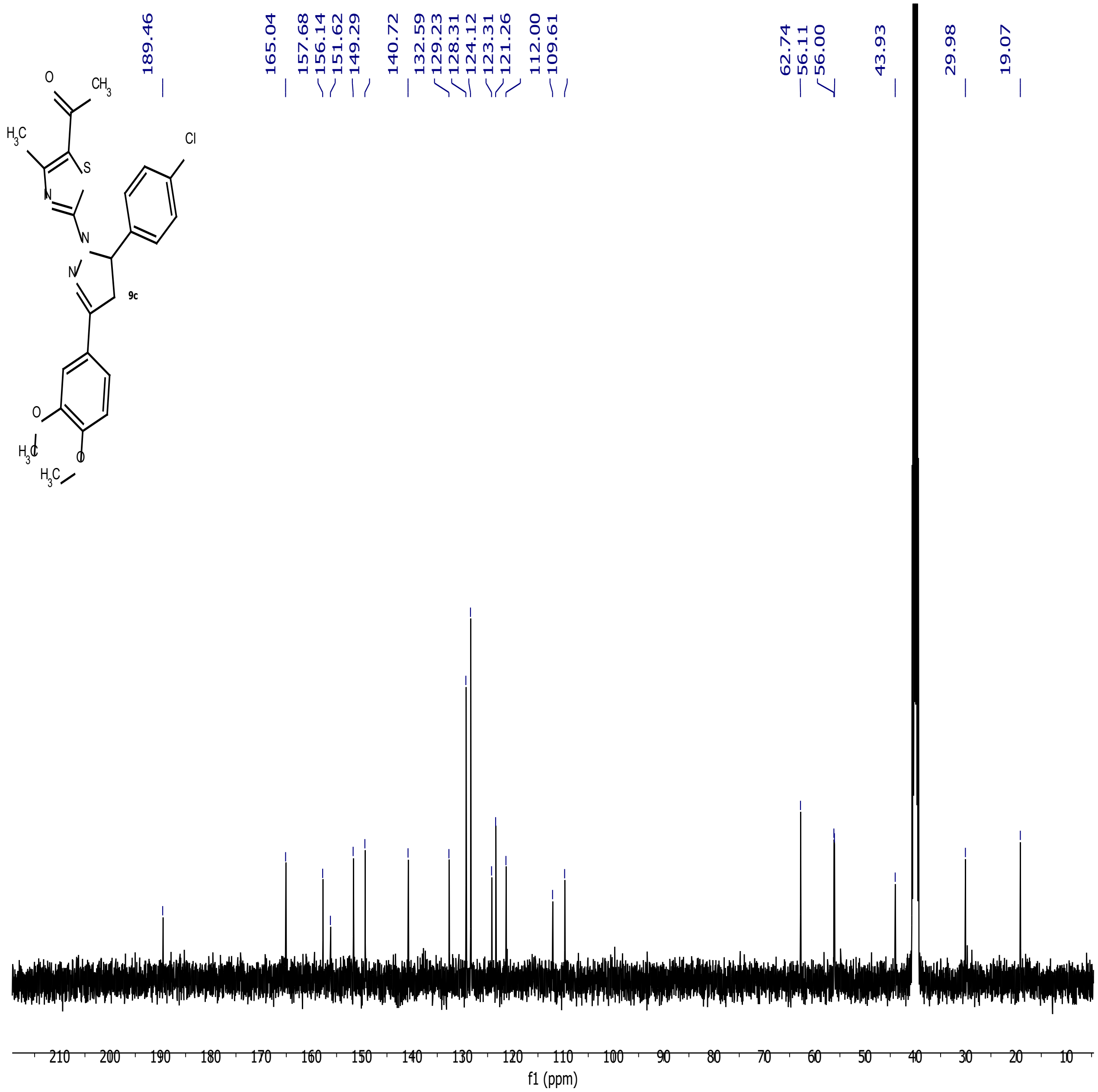

Figure S70: ^{13}C NMR of compound 9c

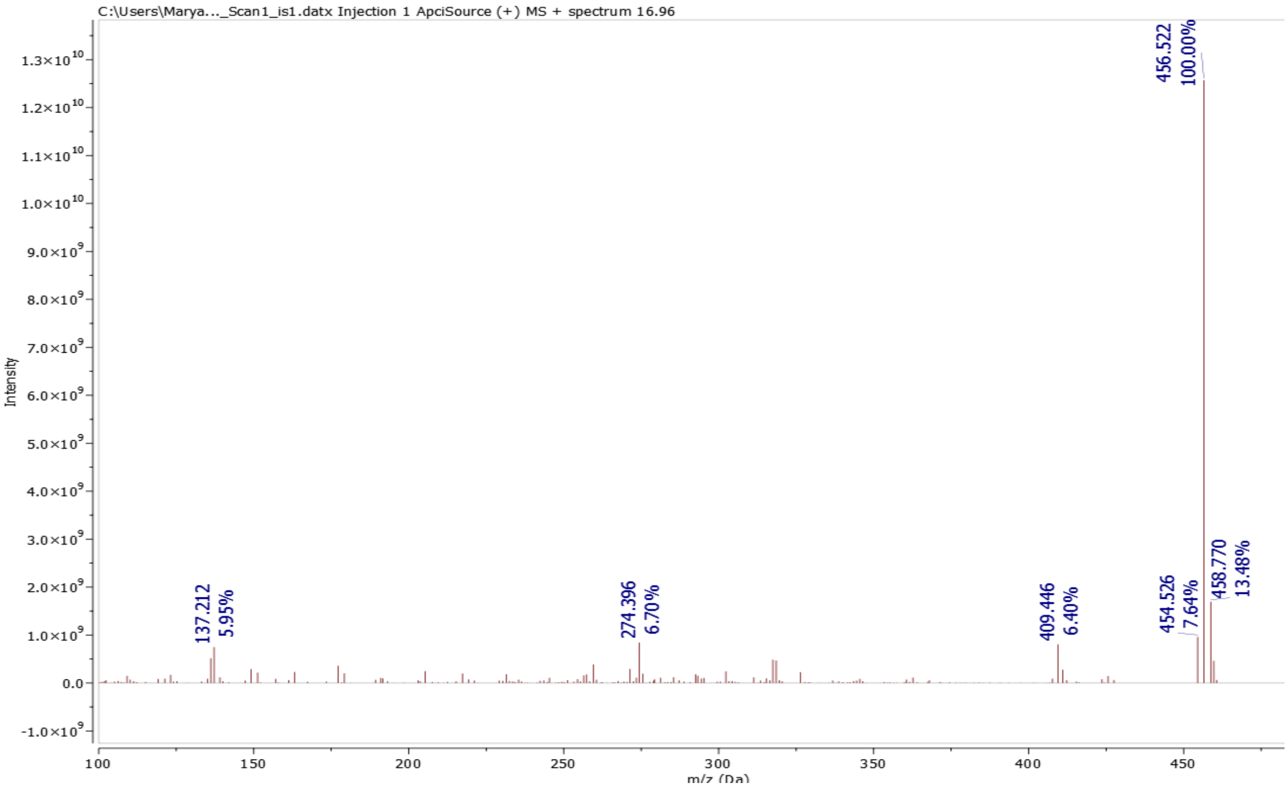

Figure S71: Mass of compound 9c

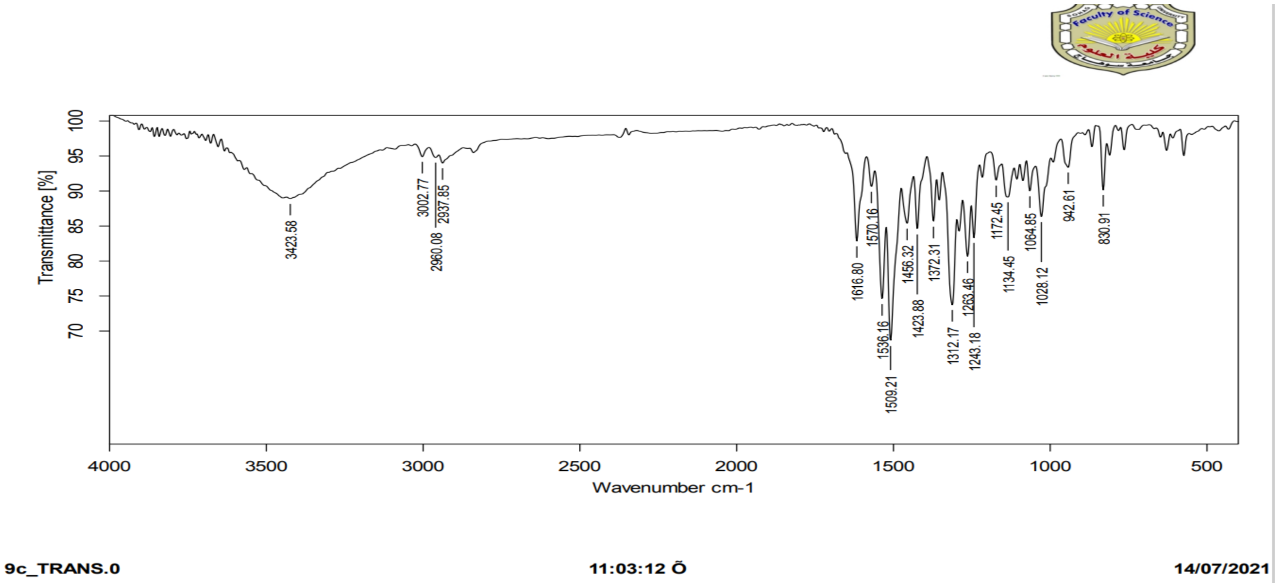

Figure S72: IR of compound 9c

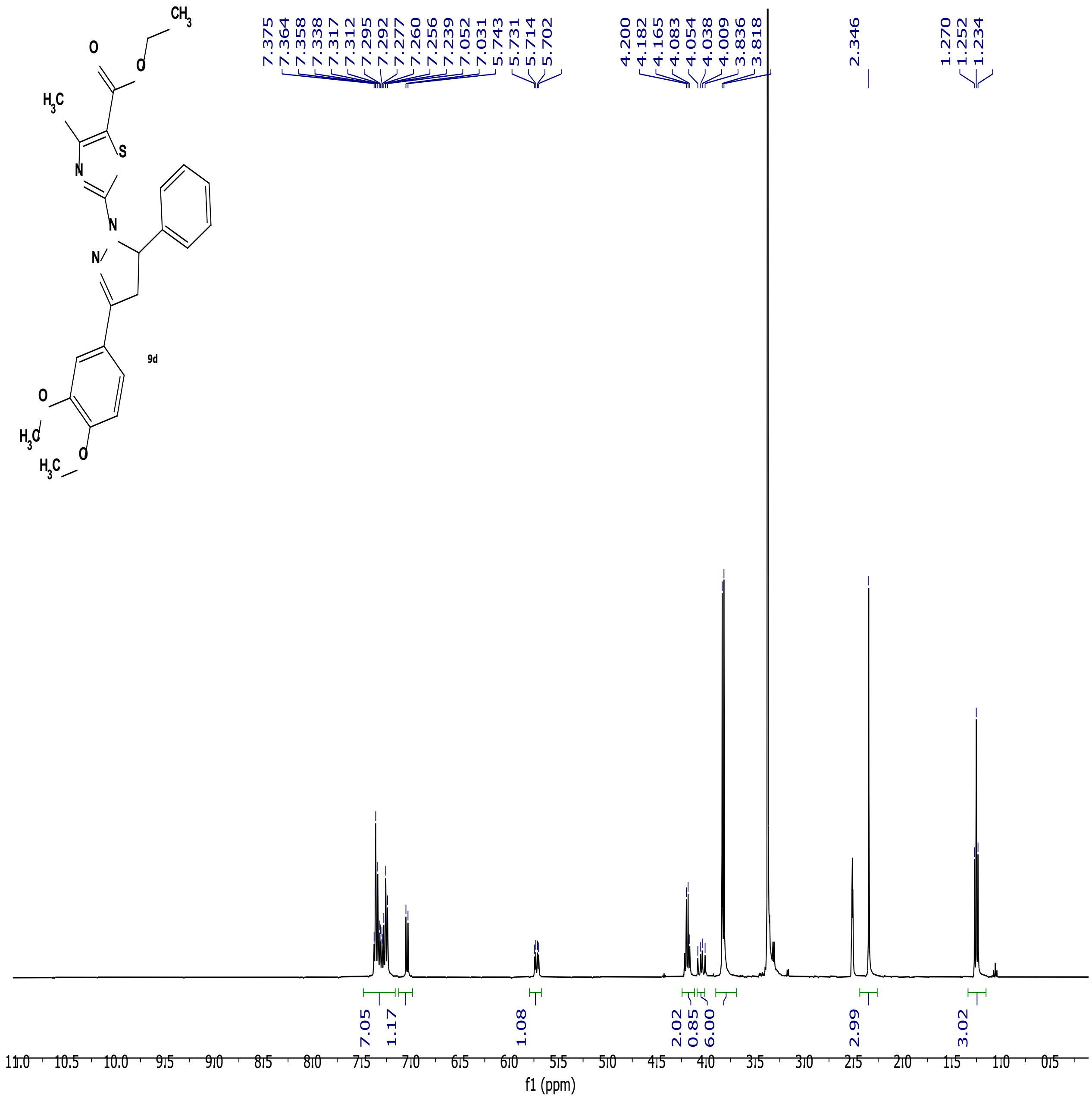

Figure S73: ^{1}H NMR of compound 9d

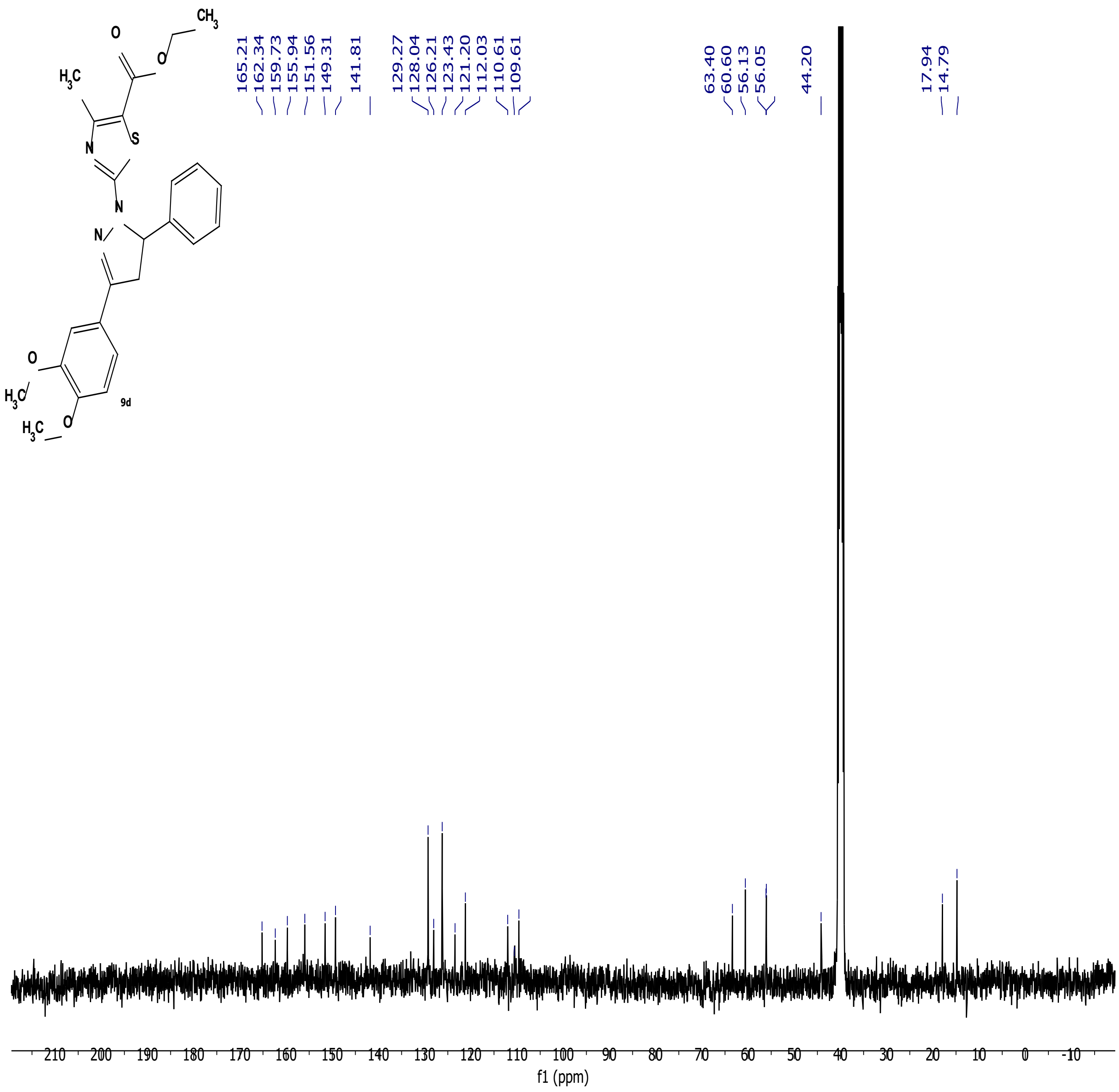

Figure S74: ^{13}C NHR of compound 9d

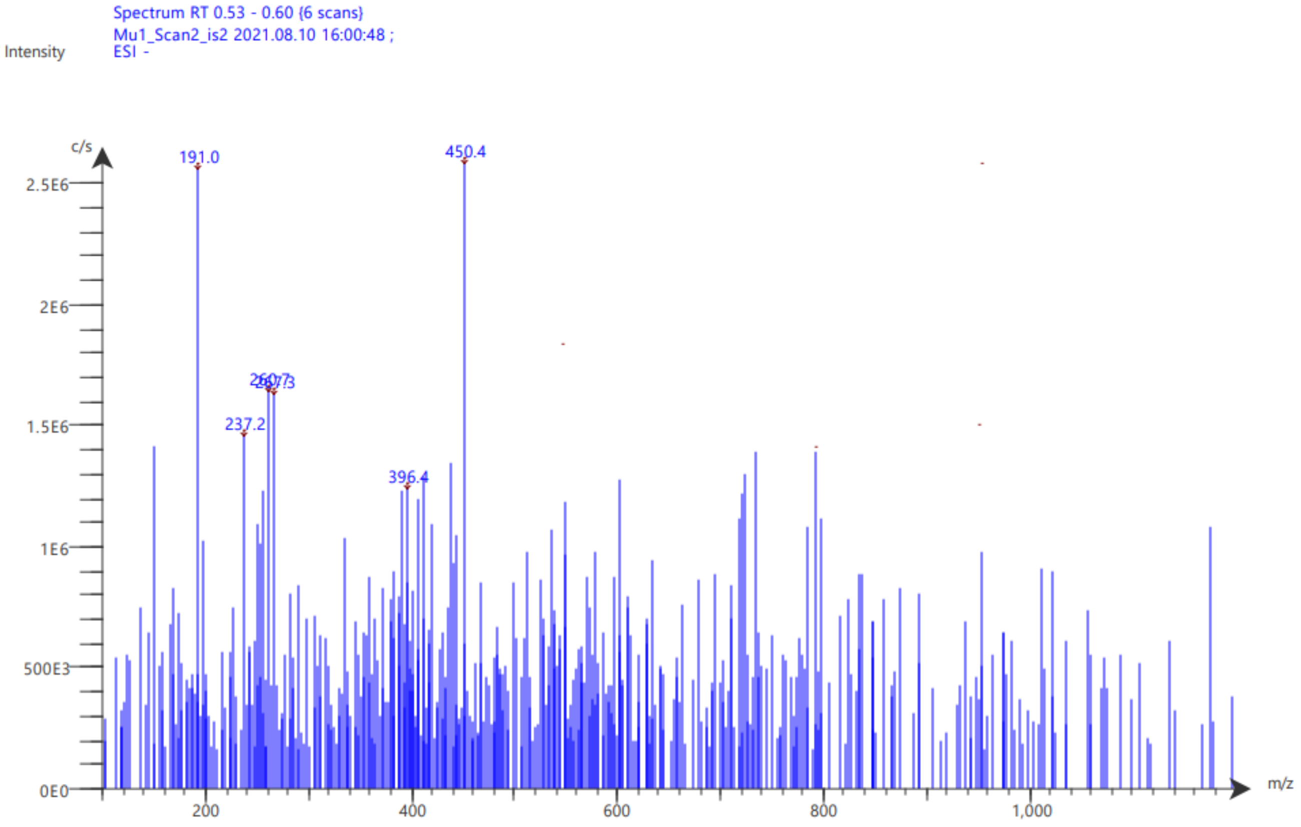

Figure S75: Mass of compound 9d

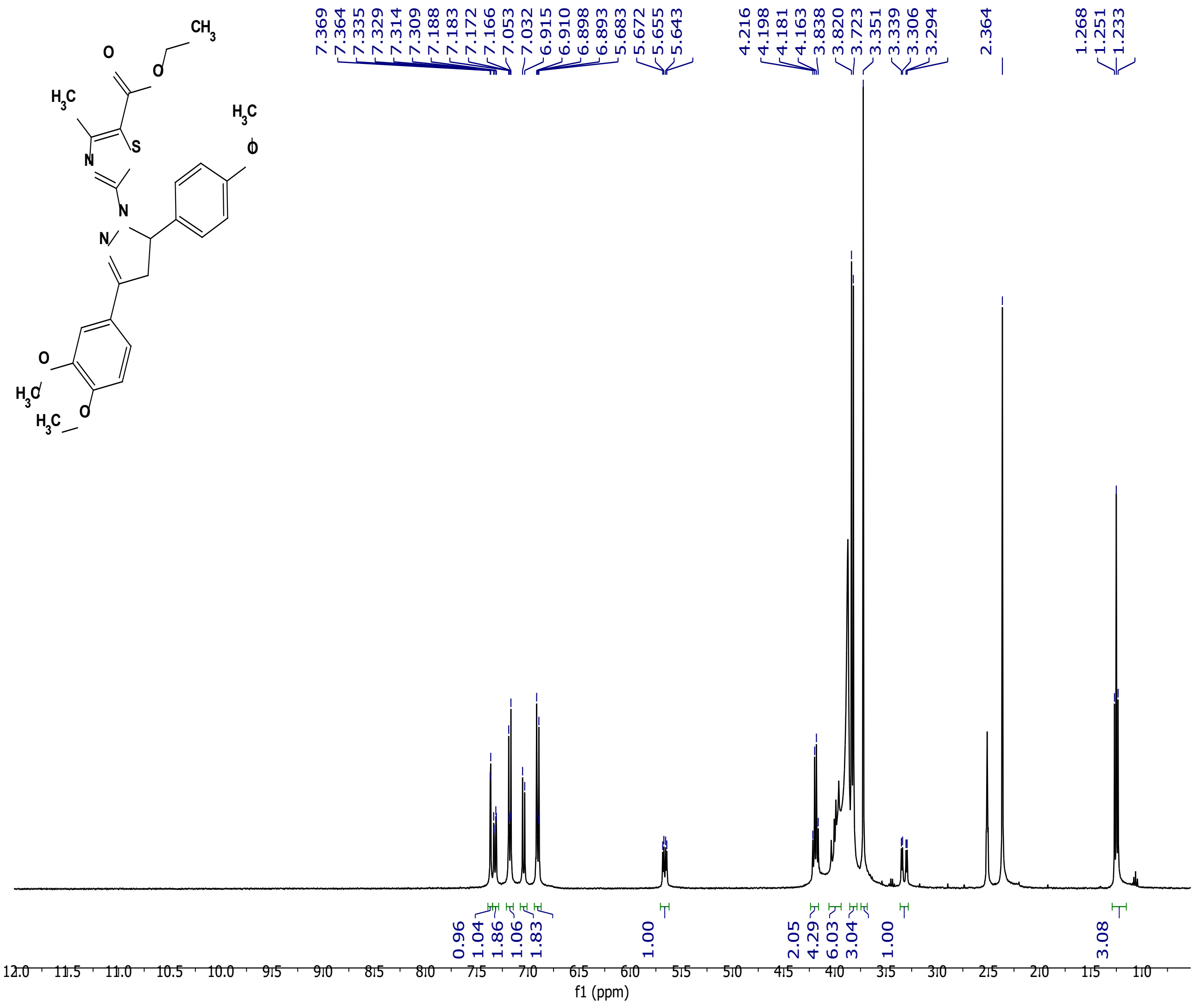

Figure S76: ^{1}H NMR of compound 9e

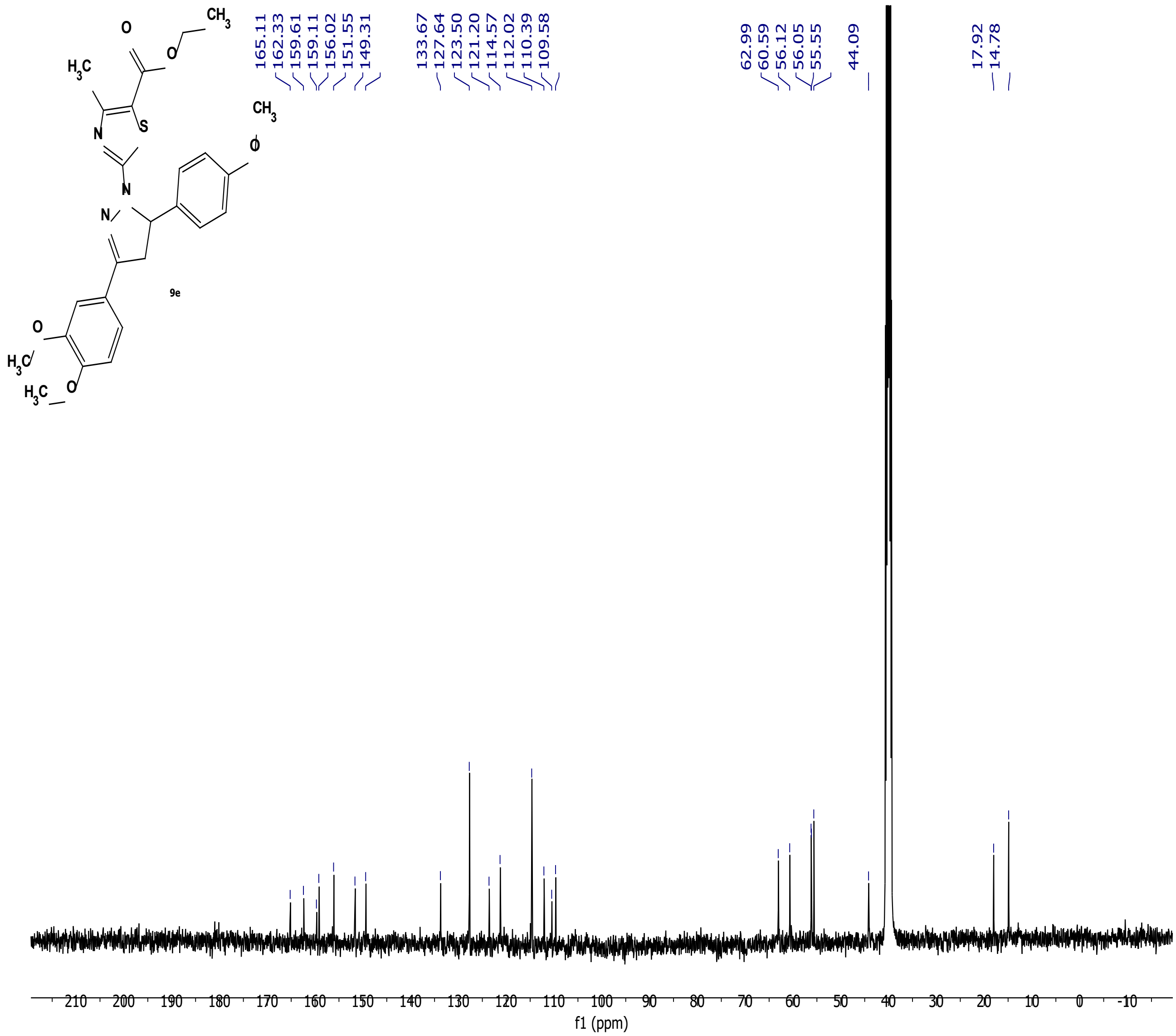

Figure S77: ^{13}C NMR of compound 9e

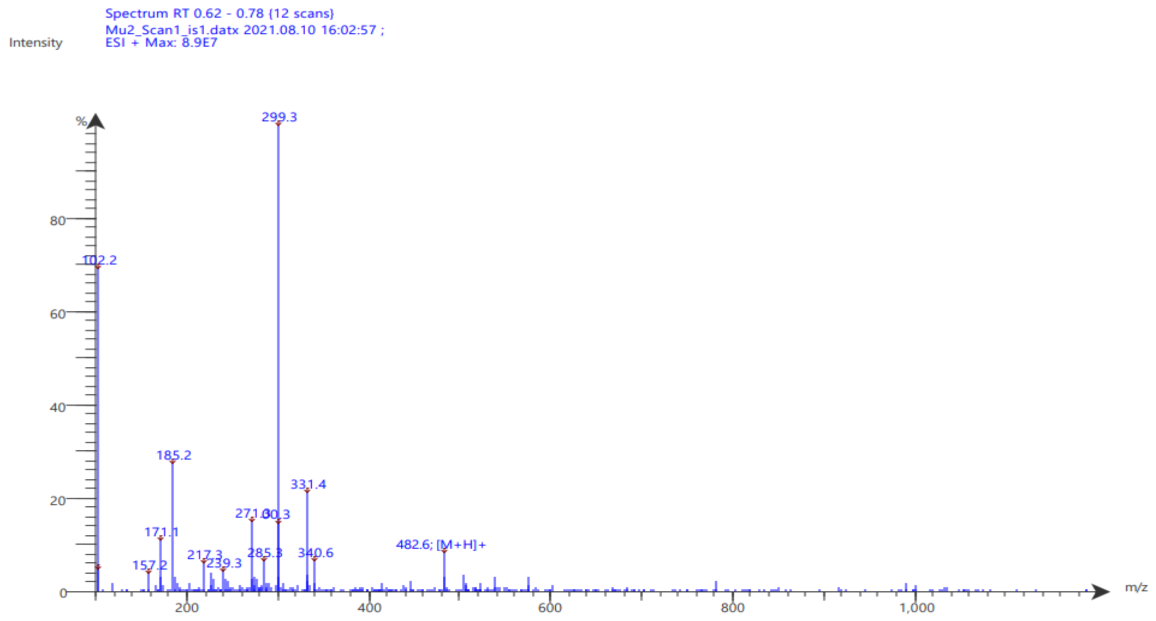

Figure S78: Mass of compound 9e

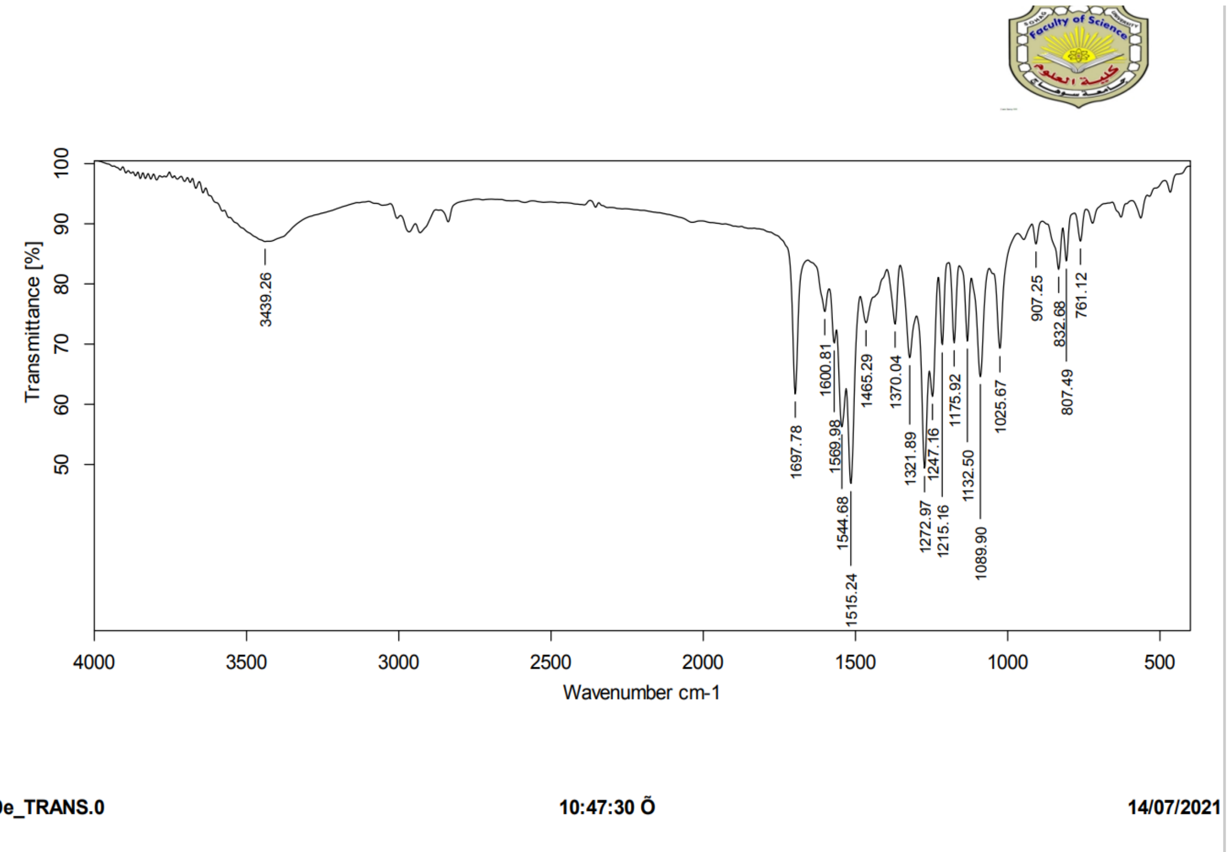

Figure S79: IR of compound 9e

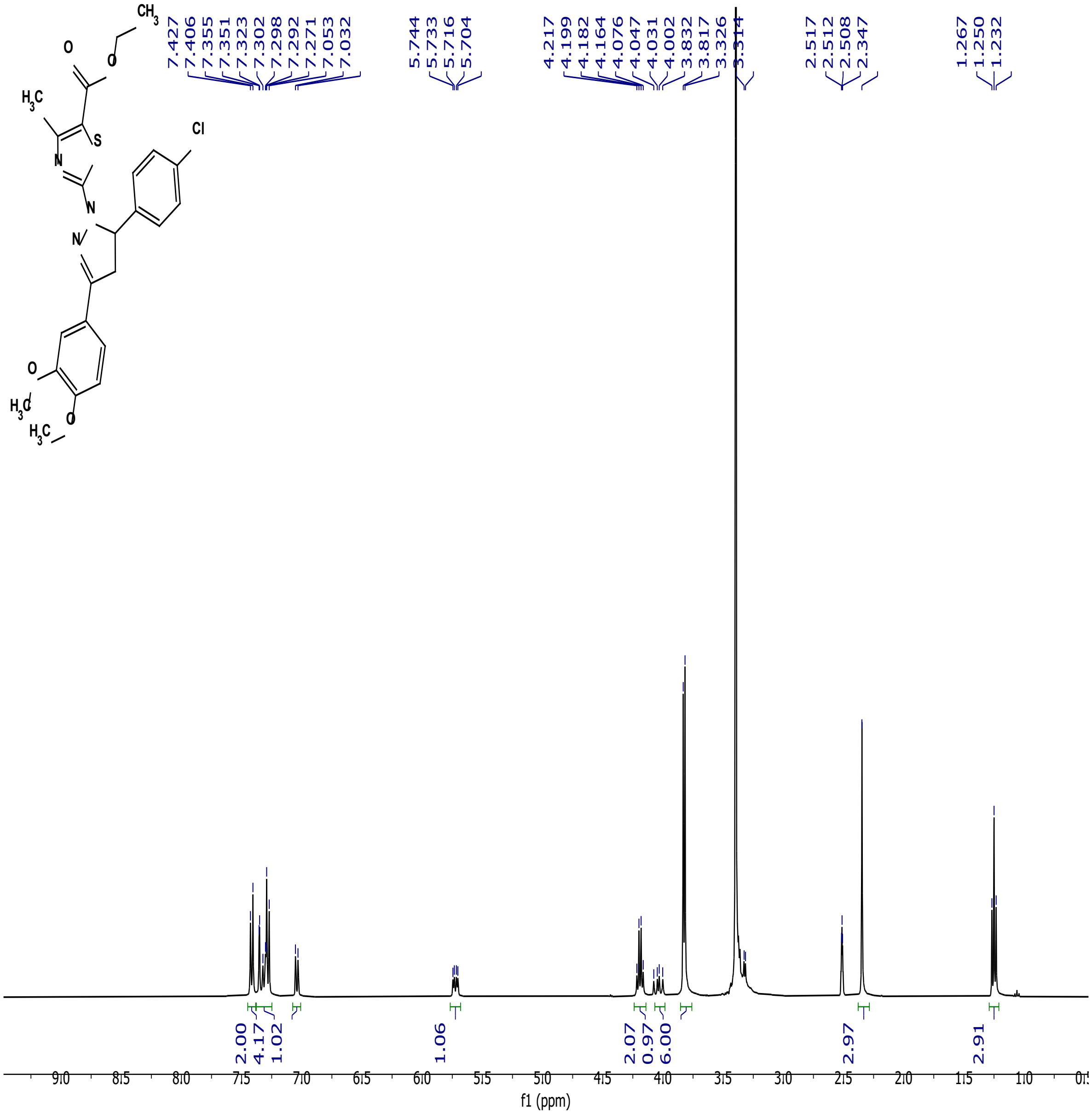

Figure S80: ^{1}H NMR of compound 9f

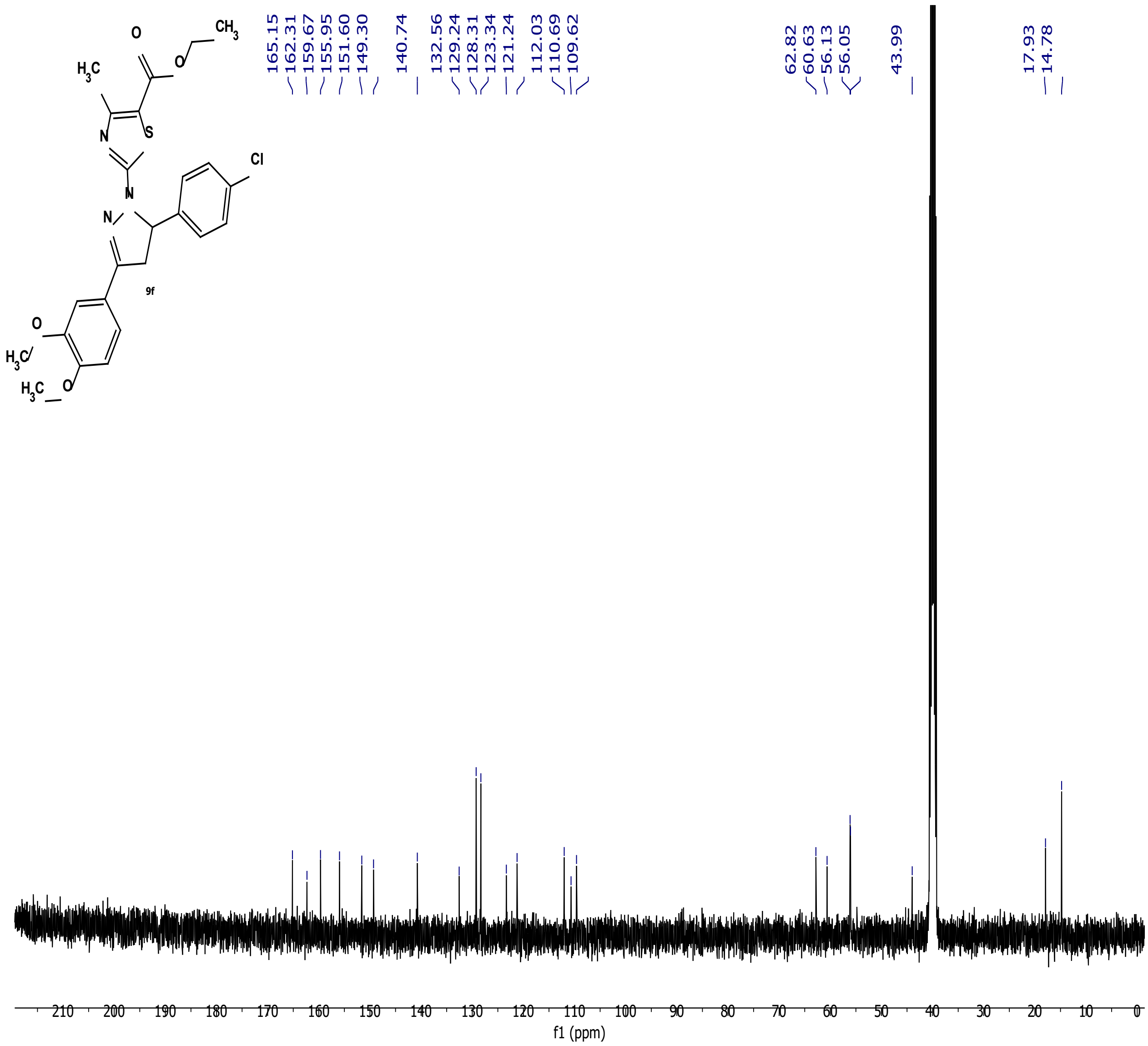

Figure S81: ^{13}C NMR of compound 9f

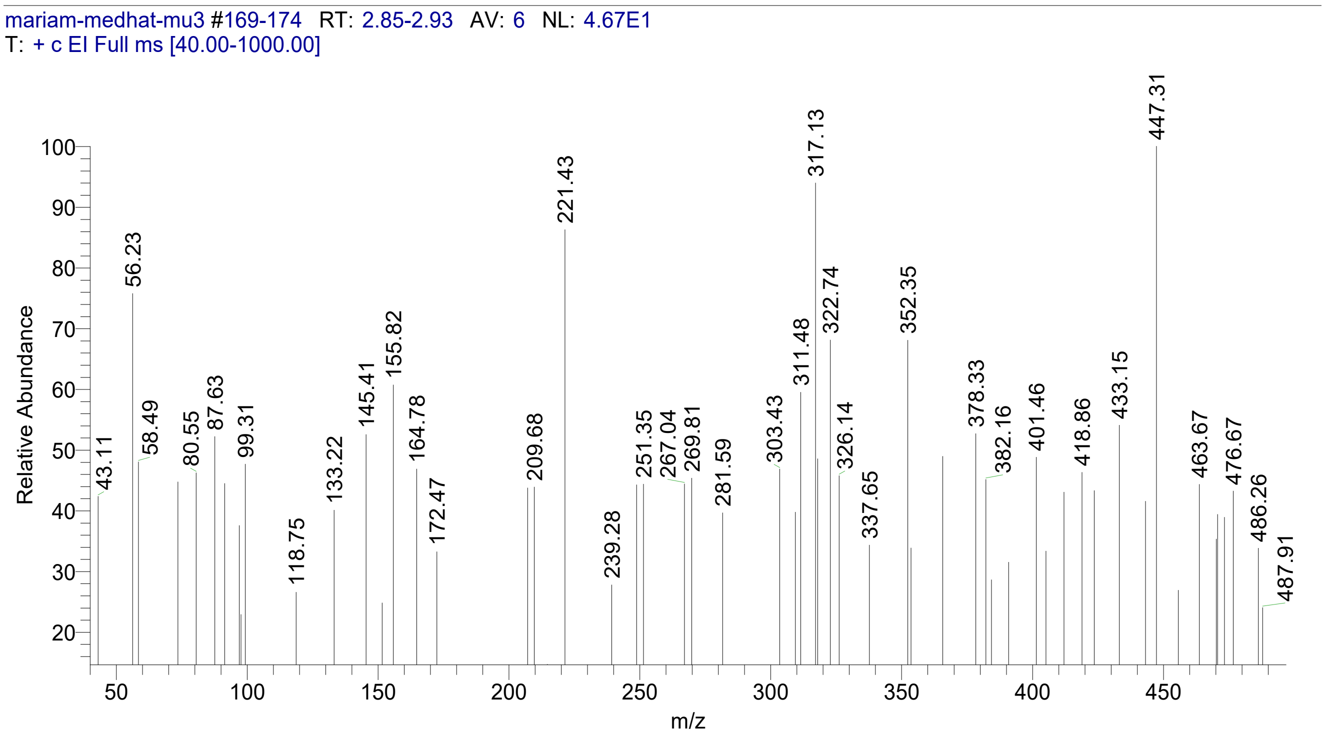

Figure S82: Mass of compound 9f

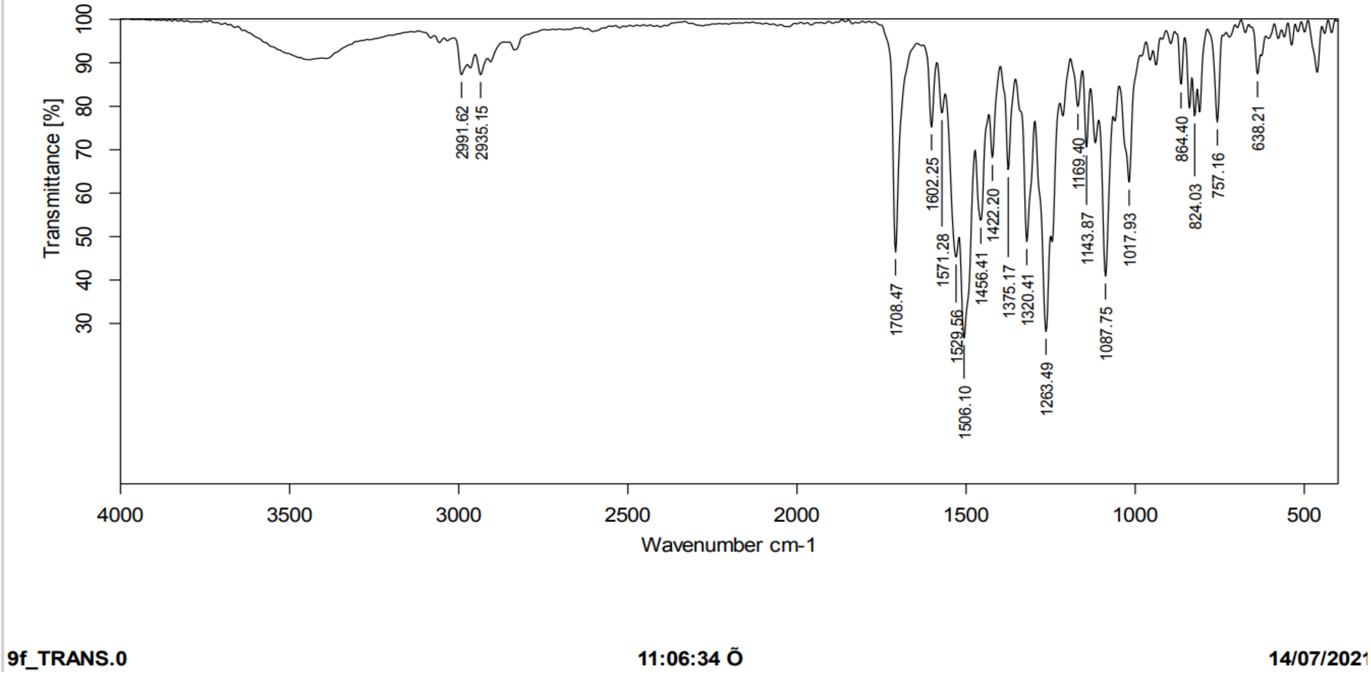

Figure S83: IR of compound 9f

Al-Azhar University
The Regional Center for Mycology and Biotechnology

Requester Data:

Name: **Dr. Mariem Medhat Fakhry**
Authority: Faculty of Pharmacy, Egyptian Russian University

Sample Data:

Eleven samples had been submitted for elemental analysis.

Analysis Report:

Sample Code	C%	H%	N%	S%
**6a**	70.95	5.41	9.80	7.43
**6i**	65.43	4.85	9.12	6.78
**6d**	64.07	5.69	9.53	7.23
**9e**	62.51	5.83	8.97	6.81
**9f**	59.58	5.06	8.89	6.72
**9b**	64.09	5.67	9.48	7.28
**9c**	60.81	5.08	9.41	7.14
**9a**	65.78	5.63	10.15	7.68
**3c**	57.65	4.97	11.45	8.70
**6e**	69.04	5.60	9.17	6.93
**6f**	69.47	5.78	8.81	6.72

INVESTIGATOR **DIRECTOR**

M. Elnasser H. Sheikh

جامعة الأزهر
المركز الإقليمي للفطريات وتطبيقاتها

Al-Azhar University Campus - Nasr City, Cairo, Egypt.
Tel: 0202 22620373 Fax : 0202 22620373
E.mail:rcmb@azhar.edu.eg
Website: http://www.azhar.edu.eg.htm * http://www.azhar.edu.eg/pages/fungi_center.htm
Facebook : RCMB AZHAR P.O. box mail : 11751 Nasr City Cairo, Egypt.

Al-Azhar University

The Regional Center for Mycology and Biotechnology

Requester Data:

Name: **Dr. Mariem Medhat Fakhry**
Authority: Faculty of Pharmacy, Egyptian Russian University

Sample Data:

Ten samples had been submitted for elemental analysis.

Analysis Report:

Sample Code	C%	H%	N%	S%
6L	61.40	4.29	8.51	6.37
6K	63.51	4.45	8.75	6.42
3a	63.59	5.78	12.52	9.48
3b	61.72	5.86	11.58	8.75
6c	67.78	5.01	9.42	6.85
6g	66.51	5.11	8.75	6.68
6d	65.87	4.80	9.09	6.85
6h	64.23	4.93	8.47	6.50
6b	71.42	5.67	9.49	7.18
6j	66.46	5.11	8.79	6.61

INVESTIGATOR **DIRECTOR**

Al-Azhar University Campus - Nasr City, Cairo, Egypt.
Tel: 0202 22620373 Fax : 0202 22620373
E.mail:rcmb@azhar.edu.eg
Website: http://www.azhar.edu.eg.htm * http://www.azhar.edu.eg/pages/fungi_center.htm
Facebook : RCMB AZHAR P.O. box mail : 11751 Nasr City Cairo, Egypt.

Figure S84: Elemental analysis (CHN) for 3a-c, 6a-l, 9a-f

Table S1: Predicted properties and Lipinski's parameters for target compounds and lapatinib as a reference.

Compound No.	RB	HBA	HBD	MR	TPSA	CLogP	Log S	Lipinski's violations	Veber's violations
**3a**	5	3	1	105.51	92.17	2.66	-3.68	0	0
**3b**	6	4	1	112	101.4	2.74	-3.75	0	0
**3c**	5	3	1	110.52	92.17	3.2	-4.27	0	0
**6a**	6	4	0	136.35	75.19	5.04	-6.49	0	0
**6b**	6	4	0	141.32	75.19	5.36	-6.79	0	0
**6c**	6	5	0	136.31	75.19	5.35	-6.65	0	0
**6d**	6	4	0	141.36	75.19	5.57	-7.08	1	0
**6e**	7	5	0	142.85	84.42	5.06	-6.56	0	0
**6f**	7	5	0	147.81	84.42	5.42	-6.86	0	0
**6g**	7	6	0	142.8	84.42	5.39	-6.72	0	0
**6h**	7	5	0	147.86	84.42	5.61	-7.15	1	0
**6i**	6	4	0	141.36	75.19	5.57	-7.08	1	0
**6j**	6	4	0	146.33	75.19	5.96	-7.38	1	0
**6k**	6	5	0	141.32	75.19	5.94	-7.24	1	0
**6l**	6	4	0	146.37	75.19	6.16	-7.68	2	0
**9a**	6	5	0	126.08	92.26	4.1	-5.47	0	0
**9b**	7	6	0	132.57	101.49	4.18	-5.54	0	0
**9c**	6	5	0	131.09	92.26	4.64	-6.07	0	0
**9d**	8	6	0	131.97	101.49	4.43	-5.83	0	0
**9e**	9	7	0	138.46	110.72	4.44	-5.91	0	0
**9f**	8	6	0	136.98	101.49	4.99	-6.43	0	0
**lapatinib**	11	8	2	153.88	114.73	5.19	-7.27	1	0

Table S2: Predicted *in silico* ADME properties for target compounds.

Compound No.	HIA	BPB	BBB	Skin P
**3a**	97.284	88.037	0.0429	-3.0845
**3b**	97.856	87.451	0.08	-3.2976
**3c**	96.954	89.75	0.0593	-3.1369
**6a**	97.567	93.43	2.7355	-2.3729
**6b**	97.598	92.803	2.3387	-2.3461
**6c**	97.569	91.82	1.4974	-2.6339
**6d**	97.767	91.79	1.2769	-2.402
**6e**	97.623	92.213	2.8912	-2.5218
**6f**	97.616	91.775	2.8309	-2.507
**6g**	97.621	91.299	2.057	-2.8202
**6h**	97.7	90.979	1.9193	-2.5594
**6i**	97.767	91.954	1.7181	-2.402
**6j**	97.819	91.629	1.2075	-2.3803
**6k**	97.771	91.83	0.7849	-2.6709

**6l**	98.012	93.086	0.6125	-2.3609
**9a**	99.011	90.105	1.023	-3.2079
**9b**	99.547	89.727	0.8536	-3.4273
**9c**	98.186	91.34	1.3662	-3.2681
**9d**	99.547	90.086	1.118	-3.1375
**9e**	99.845	89.995	0.88	-3.3557
**9f**	98.786	90.223	1.2438	-3.2
**Lapatinib**	96.867	97.564	0.03139	-2.324

Table S3: Toxicity prediction using Osiris server.

Compound no.	Mutagenic	Irritant	Reproductive effect	Drug-score
**3a**	no	no	yes	0.46
**3b**	no	no	yes	0.45
**3c**	no	no	yes	0.39
**6a**	no	no	no	0.19
**6b**	no	no	no	0.17
**6c**	no	no	no	0.17
**6d**	no	no	no	0.15
**6e**	no	no	no	0.18
**6f**	no	no	no	0.16
**6g**	no	no	no	0.16
**6h**	no	no	no	0.14
**6i**	no	no	no	0.15
**6j**	no	no	no	0.14
**6k**	no	no	no	0.14
**6l**	no	no	no	0.13
**9a**	no	no	no	0.27
**9b**	no	no	no	0.26
**9c**	no	no	no	0.2
**9d**	no	no	no	0.25
**9e**	no	no	no	0.24
**9f**	no	no	no	0.19
**lapatinib**	no	no	no	0.13
